# Regulation and remodeling of microbial symbiosis in insect metamorphosis

**DOI:** 10.1073/pnas.2304879120

**Published:** 2023-09-28

**Authors:** Sayumi Oishi, Minoru Moriyama, Masaki Mizutani, Ryo Futahashi, Takema Fukatsu

**Affiliations:** ^a^Department of Biological Sciences, Graduate School of Science, The University of Tokyo, 113-0033 Tokyo, Japan; ^b^Bioproduction Research Institute, National Institute of Advanced Industrial Science and Technology, 305-8566 Tsukuba, Japan; ^c^Graduate School of Life and Environmental Sciences, University of Tsukuba, 305-8572 Tsukuba, Japan

**Keywords:** bacterial symbiont, symbiotic organ, metamorphosis, *Plautia stali*, stinkbug

## Abstract

Many insects are dependent on microbial mutualists, which are often harbored in specialized symbiotic organs. Upon metamorphosis, insect organs are drastically reorganized. What mechanism regulates the remodeling of the symbiotic organ upon metamorphosis? How does it affect the microbial symbiont therein? Here, we addressed these fundamental issues of symbiosis by experimentally manipulating insect metamorphosis. The stinkbug *Plautia stali* possesses a midgut symbiotic organ wherein an essential bacterial symbiont resides. By RNAi of master regulator genes for metamorphosis, *Kr-h1* over nymphal traits and *E93* over adult traits, we generated precocious adults and supernumerary nymphs of *P. stali*, thereby disentangling the effects of metamorphosis, growth level, developmental stage, and other factors on the symbiotic system. Upon metamorphosis, the symbiotic organ of *P. stali* was transformed from nymph type to adult type. The supernumerary nymphs and the precocious adults, respectively, developed nymph-type and adult-type symbiotic organs not only morphologically but also transcriptomically, uncovering that metamorphic remodeling of the symbiotic organ is under the control of the MEKRE93 pathway. Transcriptomic, cytological, and biochemical analyses unveiled that the structural and transcriptomic remodeling of the symbiotic organ toward adult emergence underpins its functional extension to food digestion in addition to the original role of symbiont retention for essential nutrient production. Notably, we found that the symbiotic bacteria in the adult-type symbiotic organ up-regulated genes for production of sulfur-containing essential amino acids, methionine and cysteine, that are rich in eggs and sperm, uncovering adult-specific symbiont functioning for host reproduction and highlighting intricate host–symbiont interactions associated with insect metamorphosis.

## Significance

Microbial symbioses are ubiquitous among diverse insects. Most insects experience metamorphosis. Metamorphosis entails remodeling of external morphology and internal organs, which include symbiotic organs for hosting indispensable microbial partners. How does metamorphosis involve the symbiotic organ and the microbial symbiont therein? Here, we demonstrate that in a stinkbug *Plautia stali Kr-h1* and *E93*, the master transcriptional regulators of insect metamorphosis, govern the changes in morphology, gene expression, and biological roles of both the host symbiotic organ and the bacterial symbiont from nymphal type to adult type. These changes reflect the functional switching from symbiont retention for nymphal growth to massive food digestion for vigorous adult reproduction upon metamorphosis. Our finding highlights an intricate insect–microbe developmental integration for ensuring sustainable mutualism.

**Fig. 1.**
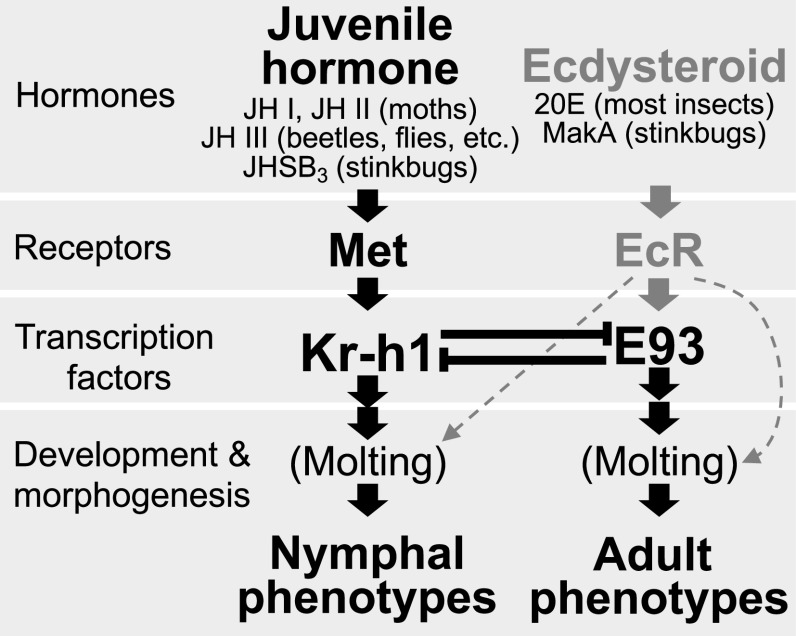
Hormonal and molecular regulatory mechanisms governing insect metamorphosis. The MEKRE93 pathway governing switching from larva/nymph to adult is indicated in black, whereas the ecdysteroid action mainly to trigger molting is shown in gray. Abbreviations: 20E, 20-hydroxyecdysone; EcR, ecdysone receptor; JH I, juvenile hormone I; JH II, juvenile hormone II; JH III, juvenile hormone III; JHSB_3_, juvenile hormone III skipped bisepoxide; MakA, makisterone A; Met, methoprene-tolerant.

Insects represent the majority of the biodiversity in the terrestrial ecosystem (1). One of the factors that have driven their diversification and prosperity is metamorphosis, by which the insects drastically change their morphology, physiology, behavior, and ecology from larvae/nymphs to adults. Some insects metamorphose from larvae through pupae to adults, the so-called complete metamorphosis or holometaboly, whereas other insects directly metamorphose from nymphs to adults, the so-called incomplete metamorphosis or hemimetaboly (2). While juvenile hormone (JH) and molting hormone (ecdysteroid) are the principal endocrinological regulators of insect metamorphosis (3), the JH receptor gene *methoprene-tolerant* (*Met*), the master regulator gene of larval/nymphal traits *Krüppel homolog 1* (*Kr-h1*), and the master regulator gene of adult traits *ecdysone-induced protein 93* (*E93*) comprise the gene regulatory network governing the insect metamorphosis, known as the MEKRE93 pathway (Fig. 1) (4). Under a high level of JH, *Kr-h1* is up-regulated via the JH receptor Met, which activates downstream genes for larval/nymphal development and maintains larval/nymphal molting in response to an ecdysteroid pulse. Under a reduced level of JH, Kr-h1 is down-regulated, whereby E93 is released from Kr-h1-mediated suppression, and the up-regulated E93 activates downstream genes for adult development and results in adult molting in response to an ecdysteroid pulse (Fig. 1). Notably, the MEKRE93 pathway is highly conserved across holometabolous and hemimetabolous insect groups, reflecting the ancient evolutionary origin of insect metamorphosis (4, 5).

Besides metamorphosis, symbiotic association with microorganisms is also among the factors that have significantly contributed to the diversification and prosperity of insects by helping food digestion (6), provisioning essential nutrients (7), facilitating resistance against parasites and pathogens (8), assisting tolerance to abiotic stressors (9), etc. In such mutualistic host–symbiont associations, the host insects often develop specialized organs for harboring their specific microbial symbionts, the so-called symbiotic organs (10, 11). In some insects like aphids, ants, and weevils, their essential microbial partners are endocellularly harbored in specialized cells and organs for symbiosis, the so-called bacteriocytes and the bacteriomes, respectively (12–14). In other insects like stinkbugs and leaf beetles, their indispensable microbial associates are extracellularly harbored in symbiotic organs consisting of sac- or pouch-like evaginations of their alimentary tract, the so-called gastric caeca or crypts (15, 16).

The molecular mechanisms underpinning the development of the symbiotic organs are of fundamental interest in understanding the evolution of symbiosis (17, 18). In early embryonic development, the differentiation of the symbiotic cells and organs proceeds with intricate host–symbiont interactions, where co-opted Hox transcription factors play pivotal roles (19–21). In postembryonic development, the most remarkable reorganization of the symbiotic organs is observed upon metamorphosis. Particularly in holometabolous insects, since larval tissues and organs are destructively reconstructed into adult tissues and organs during the pupal period, metamorphosis imposes a profound impact on the symbiotic organs and the microbial symbionts therein (22). For example, in *Sitophilus* grain weevils, the larval bacteriocytes dissociate, migrate, deform, and disintegrate during the pupal stage in a dynamic manner, the endosymbiotic bacteria therein are conveyed to a new intestinal site and finally transferred to newly formed adult-type transient bacteriocytes, and an array of host and symbiont genes, which are presumably involved in the host morphogenesis and the symbiont migration, are expressed during the developmental process (14, 23). How such complex developmental events entailing the dynamic host–symbiont interactions are regulated and coordinated in the context of metamorphosis is still to be established.

In hemimetabolous insects, in the absence of the pupal stage, the morphogenetic changes upon metamorphosis from nymph to adult are generally less drastic in comparison with holometabolous insects (2). In aphids, for example, the structural organization of the bacteriocytes looks almost constant throughout the postembryonic development (12, 19, 24). On the other hand, some hemimetabolous insects such as lice, stinkbugs, and others were reported to exhibit remodeling of their symbiotic organs during the postembryonic development and metamorphosis (10). Of these, the brown-winged green stinkbug *Plautia stali* (Hemiptera: Pentatomidae) represents a model system in which the developmental process of the symbiotic organ has been described in detail (25).

In this study, we successfully generated precocious adult insects (=5^th^ instar adult insects) and supernumerary nymphal insects (=6^th^ instar nymphal insects) of *P. stali* by knockdown of *Kr-h1* and *E93*, the master transcriptional regulators governing the nymphal and adult traits, respectively, thereby experimentally disentangling the effects of metamorphosis, growth level, developmental stage, and other factors on the development of the symbiotic system. Using these insects, we demonstrated that the remodeling of the symbiotic organ is under the control of the MEKRE93 pathway that governs the insect metamorphosis. Transcriptomic, cytological, and functional analyses of the symbiotic organ of these insects unveiled what molecular mechanisms underpin the morphogenesis and functioning of the nymphal and adult symbiotic organs, respectively. Furthermore, we uncovered that the morphology and functioning of the symbiotic bacteria are also regulated in response to the host insect metamorphosis, highlighting intricate host–symbiont interactions in the development and morphogenesis of the host symbiotic organ.

## Results and Discussion

### Remodeling of the Midgut Symbiotic Organ upon Metamorphosis of *P. stali*.

First, we observed the development of the midgut symbiotic organ, particularly focusing on the period of final (=5^th^) nymphal instar to adulthood. The midgut of *P. stali* consists of structurally distinct regions, M1, M2, M3, M4b, and M4 from the oral side to the anal side, of which the most posterior M4 region is specialized as a symbiotic organ with numerous crypts arranged in four rows (*SI Appendix*, Fig. S1*A*). The inner cavity of the crypts hosts *Pantoea*-allied symbiotic bacteria that are vertically transmitted via egg surface contamination and essential for nymphal growth (26, 27). After initial colonization of the symbiotic bacteria to the posterior midgut region, the M3-M4b junction closes, thereby isolating the posterior M4b and M4 regions as an exclusive space for the symbiotic bacteria without food flow (*SI Appendix*, Fig. S1 *B–D*). During the 5^th^ (=final) nymphal instar, notably, the structural configuration of the posterior midgut regions showed characteristic changes. In the early 5^th^ instar, the basal region of each crypt started to constrict. By the late 5^th^ instar to adult, the crypts were constricted off, by which the symbiotic bacteria were confined in the crypt cavities and isolated from the midgut main tract. Concurrently, the M3-M4b junction became thick and open, by which the food flow from the anterior midgut to the posterior midgut restored (*SI Appendix*, Fig. S1 *E–G*). These observations confirmed our previous histological descriptions on the formation process of the midgut symbiotic organ in *P. stali* (25). Whether and how is the remodeling of the symbiotic organ related to the insect metamorphosis? What regulatory and molecular mechanisms underlie these developmental processes?

### RNAi Knockdown of *E93* and *Kr-h1* Induces Nymph-like Adults and Adult-like Nymphs.

**Fig. 2.**
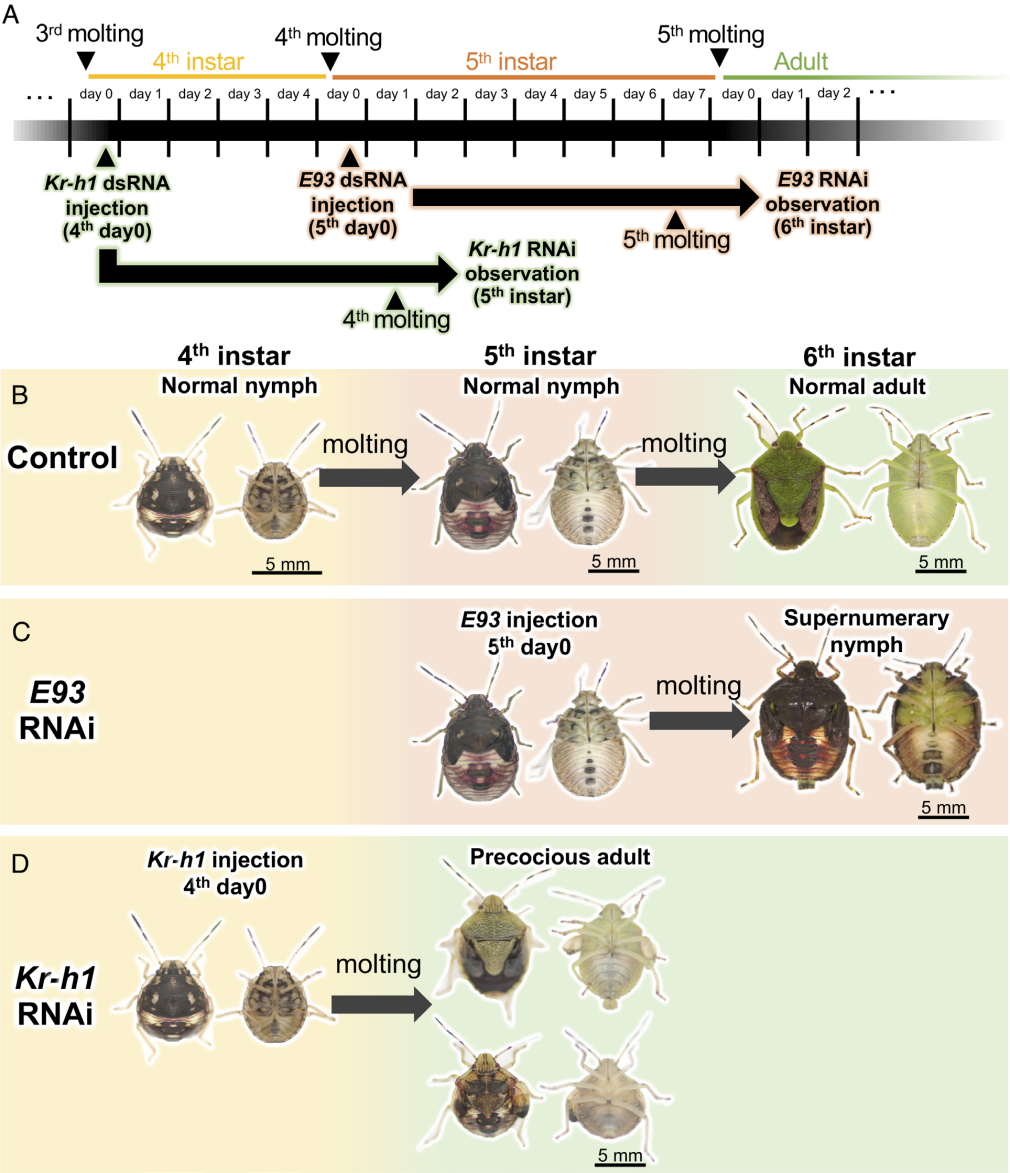
Morphological traits of *P. stali* subjected to RNAi knockdown of *E93* and *Kr-h1*. (*A*) Timing of dsRNA injection and phenotypic inspection for the RNAi experiments. (*B*) External appearance of control normal insects. (*C*) External appearance of *E93* RNAi insects. Even after adult molting, the insects retain nymph-like morphological traits, becoming “supernumerary nymphs.” (*D*) External appearance of *Kr-h1* RNAi insects. Upon molting to the 5^th^ instar, some insects show adult-like phenotypes and develop into small “precocious adults,” whereas other insects exhibit nymph-like phenotypes with partially developed wings. Also, see *SI Appendix*, Tables S1 and S2. As for the insect images in (*B–D*), the left images are dorsal view, whereas the right images are ventral view.

Previous studies reported that in a variety of hemipteran bugs including *P. stali*, RNA interference (RNAi) by double-stranded RNA (dsRNA) injection efficiently knockdown the target gene expression (20, 27, 28), and RNAi knockdown of the metamorphosis control genes induces precocious or suppressed adult-like traits upon subsequent molting in the firebug *Pyrrhocoris apterus*, the kissing bug *Rhodnius prolixus,* and the bedbug *Cimex lectularius* (29, 30). We performed RNAi knockdown experiments targeting *Kr-h1* and *E93*, the master transcriptional regulators governing the development of nymphal and adult traits, respectively (4), of *P. stali* (Fig. 2). When 5^th^ instar nymphs were injected with *E93* dsRNA, almost all insects molted to unwinged and dark nymph-like insects, the so-called supernumerary nymphs, rather than to winged and green normal adults (Fig. 2*C* and *SI Appendix*, Table S1). When 4^th^ instar nymphs were injected with *Kr-h1* dsRNA, some insects molted to small but green adult-like insects, the so-called precocious adults, some insects molted to nymph-like insects with partially developed wings, and other insects failed to molt and died (Fig. 2*D* and *SI Appendix*, Table S2). In this way, we obtained the following insect groups: normal nymphs (5^th^ instar), normal adults (6^th^ instar), supernumerary nymphs (6^th^ instar), and precocious adults (5^th^ instar). Using these insects, we were able to disentangle the effect of metamorphosis (nymph vs. adult) from the effects of growth level (relatively smaller vs. larger in size), developmental stage (5^th^ instar vs. 6^th^ instar), and other factors and to investigate what factor is relevant to the morphogenesis and structural remodeling of the symbiotic organ in *P. stali*.

### Effects of RNAi Knockdown of *E93* and *Kr-h1* on Morphogenesis of the Symbiotic Organ: Crypt Closure.

**Fig. 3.**
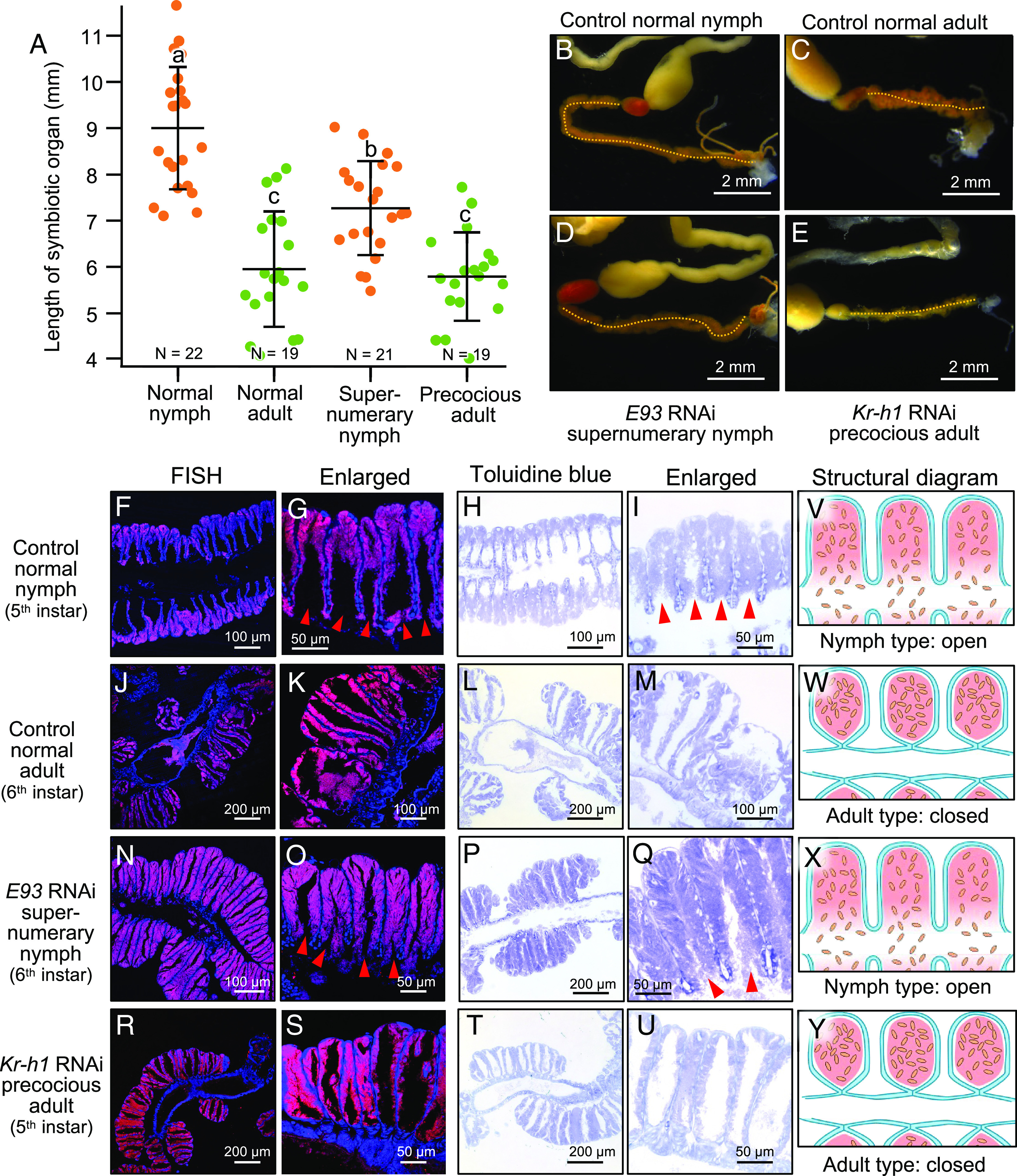
Effects of RNAi knockdown of *E93* and *Kr-h1* on the symbiotic organ of *P. stali*. (*A*) Effects on the length of the symbiotic organ. Different alphabetical letters (*a*–*c*) indicate statistically significant differences (Steel–Dwass test, *P* < 0.05). (*B–E*) Dissected alimentary tracts from a control normal nymph (*B*), a control normal adult (*C*), a *E93* RNAi supernumerary nymph (*D*), and a *Kr-h1* RNAi precocious adult (*E*). The symbiotic organ, or the midgut M4 region, is highlighted by a yellow dotted line. (*F–V*) Morphology of the symbiont-harboring crypts of the symbiotic organ. (*F–I*) Control normal nymphs. (*J–M*) Control normal adults. (*N–Q*) *E93* RNAi supernumerary nymphs. (*R–U*) *Kr-h1* RNAi precocious adults. Of the tissue section images, the two left columns are FISH images in which the symbiotic bacteria and the host nuclear DNA are visualized in red and blue, respectively, whereas the two right columns are the general tissue images stained with toluidine blue. Red arrowheads indicate the crypt openings to the midgut main tract of the symbiotic organ. (*V–Y*) Schematic diagrams of the morphological configuration of the crypts in the symbiotic organ. Note that the *E93* RNAi supernumerary nymphs exhibited the nymph-like crypt structure (*X*), whereas the *Kr-h1* RNAi precocious adults developed the adult-like crypt structure (*Y*).

In the normal development of the symbiotic organ during the 5^th^ instar of *P. stali*, constriction and closure of the basal region of numerous crypts proceeded (*SI Appendix*, Fig. S1 *D* and *G*), which resulted in morphological twisting and significant shortening of the symbiotic organ (Fig. 3 *A*–*C*) as previously reported (25). By RNAi knockdown of *E93*, the resultant supernumerary nymphs developed the symbiotic organ significantly longer than the control normal adults (Fig. 2 *A*, *C*, and *D*). By RNAi knockdown of *Kr-h1*, the resultant precocious adults exhibited the symbiotic organ significantly shorter than the control normal nymphs (Fig. 3 *A*, *B*, and *E*). Histological inspection of their symbiotic organs demonstrated that i) in the control normal nymphs, the crypts were open to the midgut main tract (Fig. 3 *F*–*I*), ii) in the control normal adults, the crypts were constricted off at the basal region and isolated from the midgut main tract (Fig. 3 *J*–*M*), iii) in the *E93* RNAi supernumerary nymphs, the crypts were open to the midgut main tract like the normal nymphs (Fig. 3 *N*–*Q*), and iv) in the *Kr-h1* RNAi precocious adults, the crypts were constricted off at the basal region and isolated from the midgut main tract like the normal adults (Fig. 3 *R*–*U*). Hence, whether the crypts are open or closed was linked to whether the insects develop into nymph (-like) or adult (-like) (Fig. 3 *V*–*Y*).

### Effects of RNAi Knockdown of *E93* and *Kr-h1* on Morphogenesis of Symbiotic Organ: Opening of M3-M4b Junction.

**Fig. 4.**
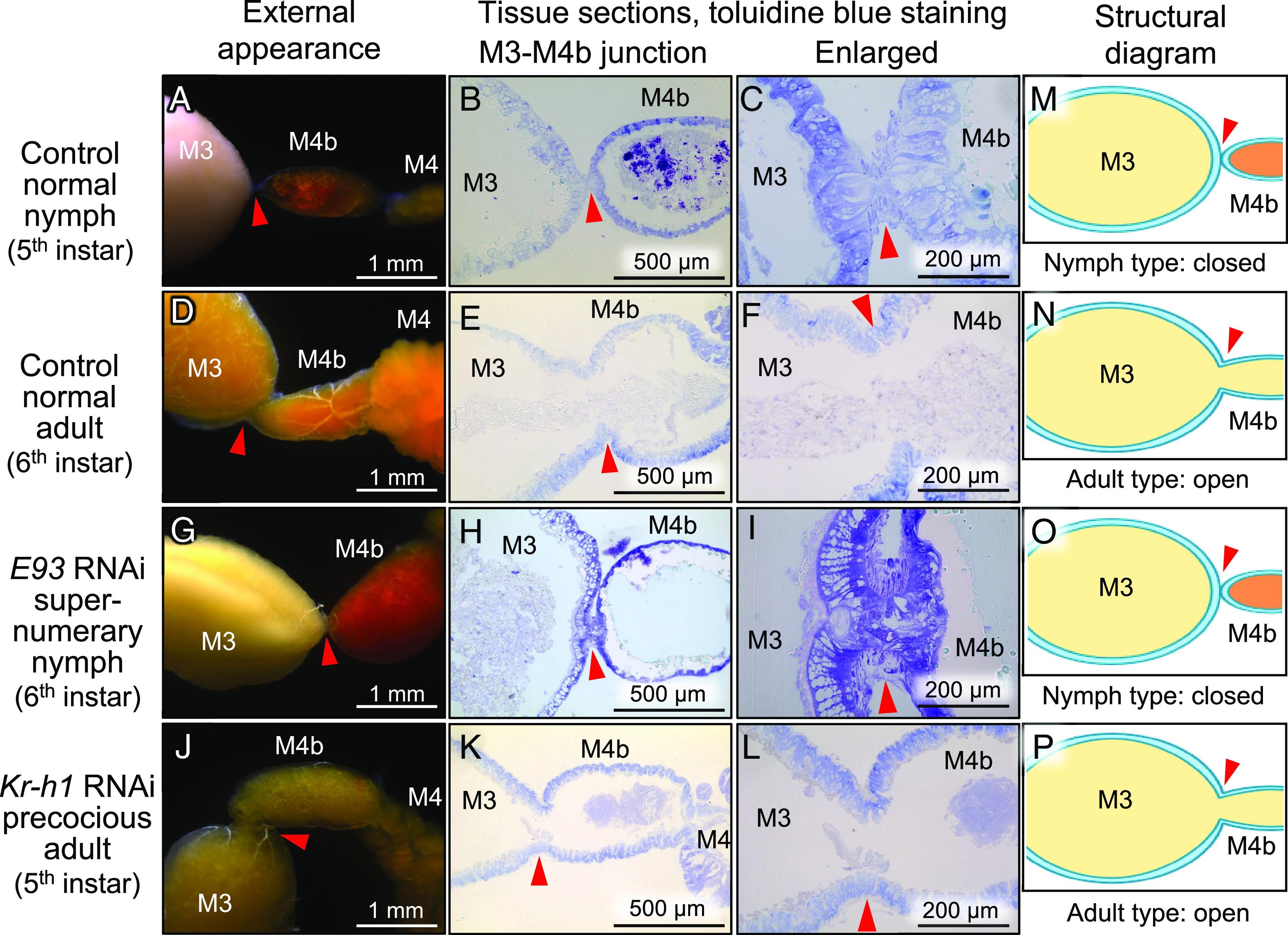
Effects of RNAi knockdown of *E93* and *Kr-h1* on the M3-M4b junction in the symbiotic organ of *P. stali*. (*A–C*) Control normal nymphs. (*D–F*) Control normal adults. (*G–I*) *E93* RNAi supernumerary nymphs. (*J–L*) *Kr-h1* RNAi precocious adults. (*A, D, G*, and *J*) External appearance of the M3-M4b junction of dissected alimentary tracts. (*B, E, H*, and *K*) Sectioned tissue images of the M3-M4b junction. (*C, F, I*, and *L*) Enlarged images of the M3-M4b junction. (*M–P*) Schematic diagrams of the morphological configuration of the M3-M4b junction in the symbiotic organ. Note that the *E93* RNAi supernumerary nymphs exhibited the nymph-like closed junction (*O*), whereas the *Kr-h1* RNAi precocious adults developed the adult-like open junction (*P*). Red arrowheads indicate M3-M4b junctions.

In the normal development of the symbiotic organ of *P. stali,* the closed nymphal M3-M4b junction opened during the late 5^th^ instar stage (*SI Appendix*, Fig. S1 *D* and *G*), which restored normal food flow through the posterior midgut region in adult insects, as observed in the control normal nymphs (Fig. 4 *A*–*C*) and the control normal adults (Fig. 4 *D*–*F*) (25). By RNAi knockdown of *E93*, the resultant supernumerary nymphs exhibited a closed M3-M4b junction like the normal nymphs (Fig. 4 *G*–*I*), whereas by RNAi knockdown of *Kr-h1*, the resultant precocious adults showed an open M3-M4b junction like the normal adults (Fig. 4 *J*–*L*). Hence, whether the M3-M4b junction is closed or open was linked to whether the insects develop into nymph (-like) or adult (-like) (Fig. 4 *M*–*P*).

### Remodeling of Symbiotic Organ Mediated by the MEKRE93 Pathway upon Metamorphosis.

On the basis of these results, we conclude that the structural remodeling of the symbiotic organ toward adult molting, namely the crypt closure and the M3-M4b opening, is regulated under the MEKRE93 pathway upon metamorphosis. Notably, the structural remodeling of the symbiotic organ is tightly linked to metamorphosis regardless of developmental stages or body sizes of the insects, highlighting an impressive aspect of the host-symbiont association interwoven into the process of insect metamorphosis.

### Gene Expression Patterns Characteristic of Nymph-type and Adult-type Symbiotic Organs.

**Fig. 5.**
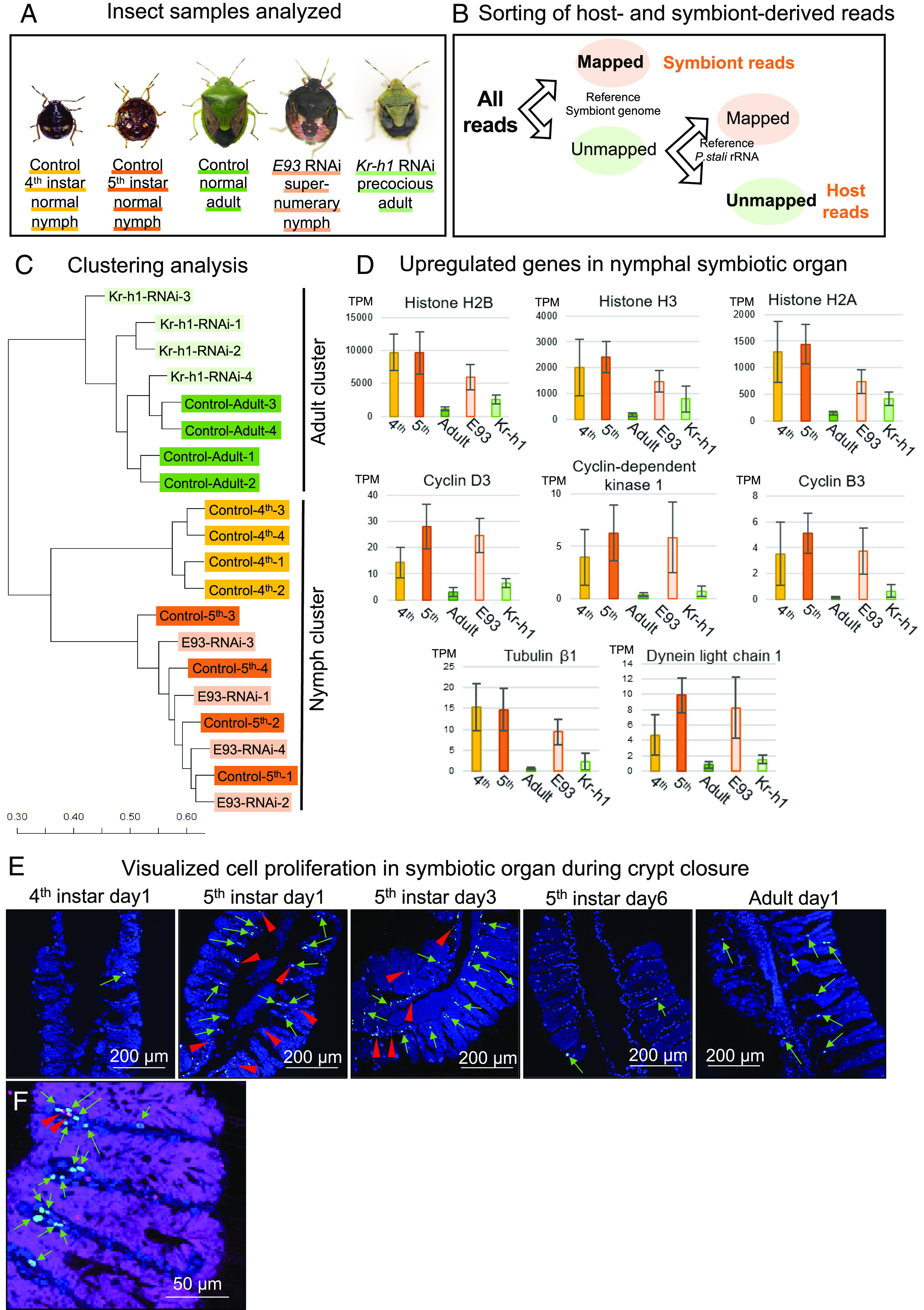
Gene expression patterns of the symbiotic organ in the developmental course of *P. stali*, and effects of RNAi knockdown of *E93* and *Kr-h1.* (*A*) Insect categories subjected to transcriptomic analysis of their midgut symbiotic organ. For each category, four insects were analyzed. (*B*) Procedure for extraction of host- and symbiont-derived RNA sequencing reads. (*C*) Hierarchical clustering analysis using the host-derived reads. Note that the *E93* RNAi supernumerary nymphs clustered with the control normal nymphs, forming the “nymph cluster,” whereas the *Kr-h1* RNAi precocious adults clustered with the control normal adults, forming the “adult cluster.” (*D*) Representative up-regulated genes in the nymphal symbiotic organs. Also see *SI Appendix*, Table S3. Mean transcripts per million (TPM) of four individuals and SD are shown for each category. Note that these genes are also up-regulated in the *E93* RNAi supernumerary nymphs. (*E*) Visualization of cell proliferation in the symbiotic organ during crypt closure. The images of control normal insects at 4^th^ instar day-1, 5^th^ instar day-1, 5^th^ instar day-3, 5^th^ instar day-6, and adult day-1 are displayed from left to right. Green, red, and blue show EdU labeling signals of DNA-synthesizing S phase cells, anti-phosphorylated histone 3 antibody signals of mitotic M phase cells, and DAPI signals of DNA, respectively. (*F*) An enlarged image of the symbiont-harboring crypts at 5^th^ instar day-1. Magenta shows FISH signals of the symbiotic bacteria, whereas green, red, and blue are the same as in (*E*). In (*E* and *F*), green arrows and red arrowheads highlight the green signals of DNA-synthesizing cells and the red signals of mitotic cells, respectively.

What molecular mechanisms underlie the morphogenesis and functioning of the nymph-type and adult-type symbiotic organs? In order to address this question, we dissected the symbiotic organs from control 4^th^ instar normal nymphs, control 5^th^ instar normal nymphs, control normal adults, *E93* RNAi supernumerary nymphs, and *Kr-h1* RNAi precocious adults (Fig. 5*A*), which were subjected to RNA sequencing to comprehensively identify host genes and symbiont genes expressed in the symbiotic organs (Fig. 5*B*). De novo assembly of the host-derived reads constructed 109,522 contigs. It was confirmed that the expression levels of *Kr-h1* and *E93* were suppressed by RNAi targeting the respective genes (*SI Appendix*, Fig. S2). Hierarchical clustering analysis of the host-derived contigs revealed that the *E93* RNAi supernumerary nymphs clustered with the control normal nymphs whereas the *Kr-h1* RNAi precocious adults grouped with the control normal adults (Fig. 5*C*), verifying that the supernumerary nymphs and the precocious adults are nymph-like and adult-like, respectively, not only morphologically but also transcriptomically.

### Differentially Expressed Genes between Nymphal and Adult Symbiotic Organs.

From the host-derived transcriptomic data, we extracted 146 differentially expressed genes between the control 5^th^ instar normal nymphs and the control normal adults (FDR *q* < 0.01), of which 46 genes were up-regulated in the normal nymphs (*SI Appendix*, Table S3) and 100 genes were up-regulated in the normal adults (*SI Appendix*, Table S4). Of 46 nymphal up-regulated genes, 15 genes exhibited significantly higher expression in the normal nymphs than in the *Kr-h1* RNAi precocious adults, and 18 genes showed significantly higher expression in the *E93* RNAi supernumerary nymphs than in the normal adults (FDR *q* < 0.01), of which 7 genes were shared between them (*SI Appendix*, Fig. S3*A*). Of 100 adult up-regulated genes, 32 genes exhibited significantly higher expression in the *Kr-h1* RNAi precocious adults than in the normal nymphs and 34 genes showed significantly higher expression in the normal adults than in the *E93* RNAi supernumerary nymphs (FDR *q* < 0.01), of which 11 genes were shared between them (*SI Appendix*, Fig. S3*B*). These patterns may favor the notion that the supernumerary nymphs and the precocious adults are nymph-like and adult-like, respectively, in the light of gene expression profiles.

### Up-regulated Genes Related to Mitotic Cell Cycle and Division in Nymph-type Symbiotic Organ.

Gene ontology (GO) analysis of the nymphal up-regulated genes identified a predominant representation of such GO terms as mitotic cell cycle process (1903047), protein heterodimerization activity (0046982), tubulin and microtubule binding (0015632 and 0008017), and others (*SI Appendix*, Fig. S4*A*). When the nymphal up-regulated genes were ranked in the order of expression levels in the 5^th^ instar nymphs, the high-ranked annotated genes were represented by genes encoding histones (rank 1, 2, and 3), cyclins and related kinase (rank 13, 27, and 31), tubulin (rank 16), dynein (rank 21), and others (*SI Appendix*, Table S3), which largely reflected the represented GO terms (*SI Appendix*, Fig. S4*A*). Notably, upregulation of these genes was also observed in the *E93* RNAi supernumerary nymphs (Fig. 5*D* and *SI Appendix*, Table S3).

### Possible Relevance of Activated Cell Division to Structural Remodeling of Symbiotic Organ upon Metamorphosis.

These gene expression patterns suggested the possibility that genes related to mitotic cell cycle and division are up-regulated in the nymphal symbiotic organ prior to metamorphosis. If so, activated cell proliferation and/or division may be preferentially observed in the symbiotic organ of 5^th^ instar nymphs. When we visualized DNA synthesizing cells by 5-ethynil-2′-deoxyuridine (EdU) incorporation and dividing cells by using anti-phospho-histone H3 antibody on tissue sections, such patterns were certainly observed: many DNA synthesizing cells and dividing cells were preferentially detected in the symbiotic organ of early and mid 5^th^ instar nymphs (Fig. 5*E*), in which the cell proliferation signals were particularly concentrated at the basal region of the crypts (Fig. 5*F*). All these transcriptomic and cytological data were supportive of the notion that in the symbiotic organ of the metamorphosing nymphs, cell division is activated at the basal region of the crypts for remodeling of the structural configuration of the symbiotic organ into crypt closure, which is under the control of the MEKRE93 pathway governing insect metamorphosis.

### Up-regulated Genes Related to Proteolysis and Digestion in Adult-type Symbiotic Organ.

**Fig. 6.**
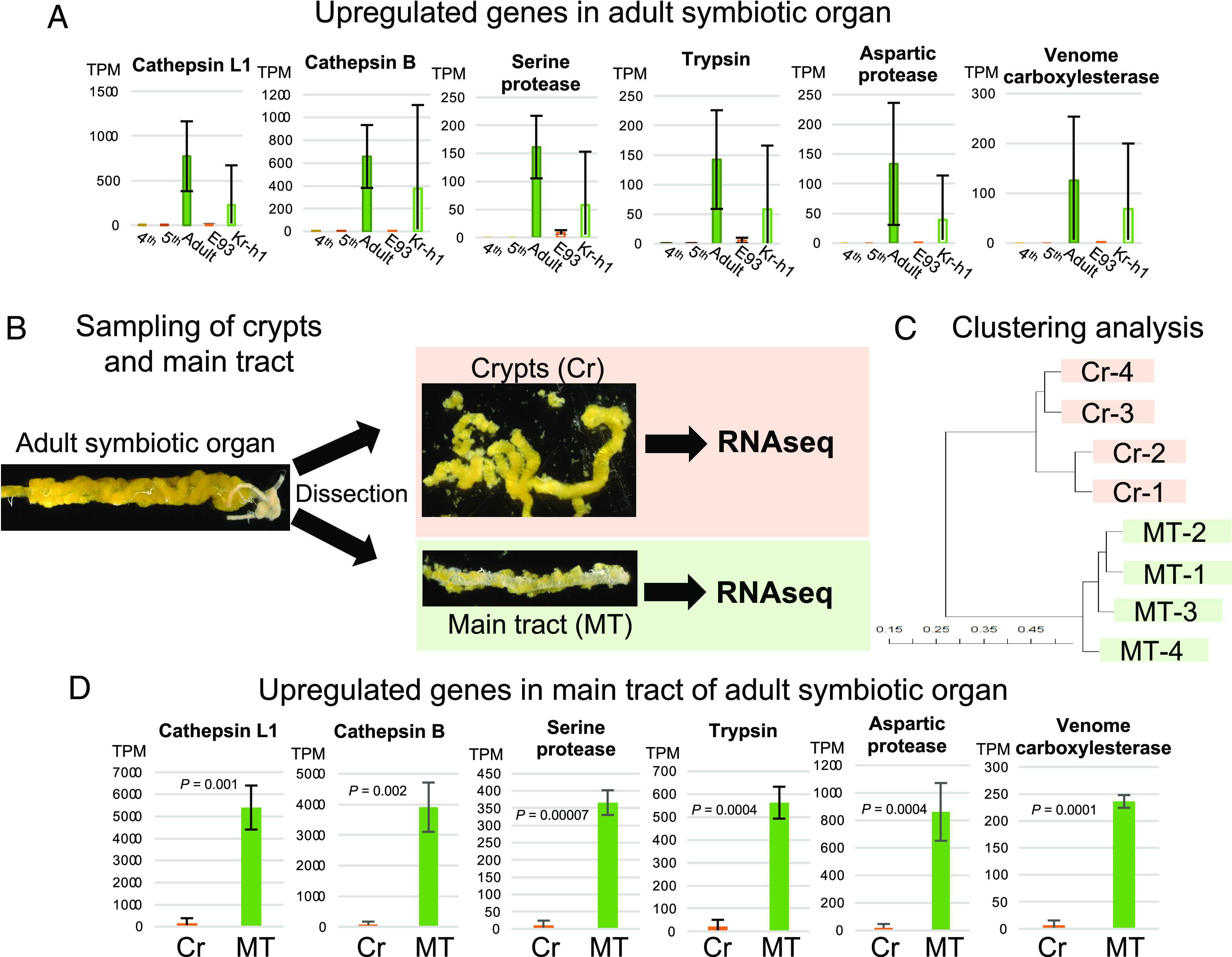
Gene expression analysis in the adult symbiotic organ of *P. stali.* (*A*) Representative up-regulated genes in the adult symbiotic organs. Also, see *SI Appendix*, Table S4. Note that these genes are also up-regulated in the *Kr-h1* RNAi precocious adults. Mean TPM of four individuals and SD are shown for each category. (*B*) Experimental procedures for dissection and transcriptomic analysis of the crypts and the main tract of the adult symbiotic organ. (*C*) Hierarchical clustering analysis based on the RNA sequencing reads derived from the crypts and the main tract of four individuals. The same numbers indicate the same individuals. (*D*) Expression levels of the representative up-regulated genes depicted in (*A*) in the crypts and the main tract. Mean TPM of four samples and SD are shown. Statistically significant differences are indicated with *P* values (*t* test).

GO analysis of the adult up-regulated genes identified a predominant representation of such GO terms as homeostatic process (0042592), feeding behavior (0007631), peptidase activity (0070011, 0008233), and others (*SI Appendix*, Fig. S4*B*). When the adult up-regulated genes were ranked in the order of expression levels in the adult insects, the high-ranked annotated genes were represented by genes encoding cathepsins (rank 4, 5, 26, and 28), serine protease (rank 18), trypsin (rank 20), aspartic protease (rank 21), carboxylesterase (rank 23), and others (*SI Appendix*, Table S4), which largely accounted for the represented GO terms (*SI Appendix*, Fig. S4*B*). Notably, upregulation of these genes was also observed in the *Kr-h1* RNAi precocious adults (Fig. 6*A* and *SI Appendix*, Table S4).

### Possible Relevance of Up-regulated Digestive Enzymes to Restored Intestinal Food Flow, Massive Feeding, and Vigorous Reproduction in Adult Insects.

These gene expression patterns suggested the possibility that genes related to proteolysis and digestion are up-regulated in the adult symbiotic organ upon metamorphosis. It should be noted that during metamorphosis, the midgut symbiotic organ exhibits morphological changes entailing the main tract formation and the food flow restoration in adult insects of *P. stali* (Figs. 3 and 4 and *SI Appendix*, Fig. S1) (25). Hence, we suspected that the up-regulated gene expression related to proteolysis and digestion may be ascribed to presumable digestive function of the main tract of the adult symbiotic organ. To test this hypothesis, we carefully dissected the symbiotic organ of each adult insect into the crypt region and the main tract region, and these samples were subjected to RNA sequencing (Fig. 6*B*). Hierarchical clustering analysis revealed that the crypt samples and the main tract samples exhibited their specific gene expression profiles (Fig. 6*C*). Strikingly, most of the up-regulated genes in the adult symbiotic organ (e.g., cathepsins, serine protease, trypsin, aspartic protease, and venome carboxylesterase; Fig. 6*A*) exhibited significantly higher, or almost specific, expression in the main tract than in the crypts (Fig. 6*D*). After molting, adult insects of *P. stali* actively feed and reproduce: under our rearing condition, young adult insects feed on raw peanuts, mate, and start laying eggs about a week after emergence, producing around 14 eggs every few days for several weeks thereafter (*SI Appendix*, Fig. S5). In this context, these transcriptomic and biochemical data were in favor of the notion that in the adult-type symbiotic organ wherein the food passage is formed by the structural remodeling upon metamorphosis, proteases and other digestive enzymes are up-regulated preferentially in the main tract region for processing the massive food flow due to active feeding of reproducing adult insects.

### Transcriptomic and Functional Differentiation within the Adult Symbiotic Organ: Crypt vs. Main Tract.

Furthermore, we scrutinized the gene expression patterns of the crypts and the main tract of the adult symbiotic organ. From the host-derived transcriptomic data, we extracted 2,004 differentially expressed genes between the crypts and the main tract (FDR *q* < 0.01), of which 515 genes were up-regulated in the crypts (*SI Appendix*, Table S5), and 1,489 genes were up-regulated in the main tract (*SI Appendix*, Table S6). GO analysis of the up-regulated genes in the crypts identified a predominant representation of GO terms related to transmembrane transport (0055085, 0006820, 0006811, and others) and transporter activity (0046943, 0005342, 0008514, and others) (*SI Appendix*, Fig. S6*A*). On the other hand, GO analysis of the up-regulated genes in the main tract uncovered a predominant representation of such GO terms as transmembrane transport (0055085, 0006812, 0005261, and 0022857), actin filament organization and related process (0007015, 0051017, 0003779, and 0051015), and peptidase activity (0006508 and 0004806) (*SI Appendix*, Fig. S6*B*). Notably, of 100 genes significantly up-regulated in the adult symbiotic organ (*SI Appendix*, Fig. S3*B* and Table S4), as many as 68 genes were up-regulated in the main tract whereas only 2 genes were up-regulated in the crypts (*SI Appendix*, Table S7). On the basis of these patterns, we propose the following perspective as to how the crypts and the main tract are functionally differentiated in the adult symbiotic organ. i) In the crypts and the main tract, different gene sets for transmembrane transport are up-regulated, which are probably involved in nutrient absorption and host-symbiont metabolite exchange in the midgut symbiotic organ. ii) In the main tract, actin and related genes are up-regulated, which are presumably for construction of visceral muscles for active peristaltic movements of the adult gut tube for digestion. In fact, when the adult symbiotic organ was observed by phalloidin staining, a strong actin signal was detected in the main tract of the symbiotic organ. (*SI Appendix*, Fig. S7). iii) In the main tract, peptidase genes are up-regulated, which are likely for the resumed food digestion in the adult symbiotic organ. iv) The majority of the up-regulated genes in the adult symbiotic organ are expressed not in the crypts but in the main tract, which may reflect the developmental process that the nymphal symbiotic organ consisting of the crypts for symbiont retention is transformed into the adult symbiotic organ in which the main tract for food digestion is newly formed in addition to the original crypts. In this way, the structural remodeling of the symbiotic organ entails the molecular, physiological, and functional differentiation into the specialized regions for symbiont retention and food digestion upon metamorphosis, which is under the control of the MEKRE93 pathway.

### Effects of RNAi Knockdown of *E93* and *Kr-h1* on Morphology of Symbiotic Bacteria.

Finally, we investigated how the structural and functional remodeling of the symbiotic organ upon metamorphosis affects the symbiotic bacteria therein. In the control untreated insects, we observed that the cell size of the symbiotic bacteria increased upon metamorphosis: the average length of symbiont cells was consistently shorter than 3 µm in 4^th^ and 5^th^ instar nymphs but became longer than 3 µm in adults (*SI Appendix*, Fig. S8*A*). In the *E93* RNAi supernumerary nymphs, the symbiotic bacteria were shorter in size, which was similar to the normal nymphs rather than to the normal adults (*SI Appendix*, Fig. S8 *B* and *C*). In the *Kr-h1* RNAi precocious adults, the symbiotic bacteria were longer in size, which was similar to the normal adults rather than to the normal nymphs (*SI Appendix*, Fig. S8 *B* and *C*). These observations suggested the possibility that intriguingly, some biological conditions of the symbiotic bacteria may be affected by the transformation of the symbiotic organ upon host insect metamorphosis.

### Different Gene Expression Patterns of Symbiotic Bacteria in Nymph-type and Adult-type Symbiotic Organs.

**Fig. 7.**
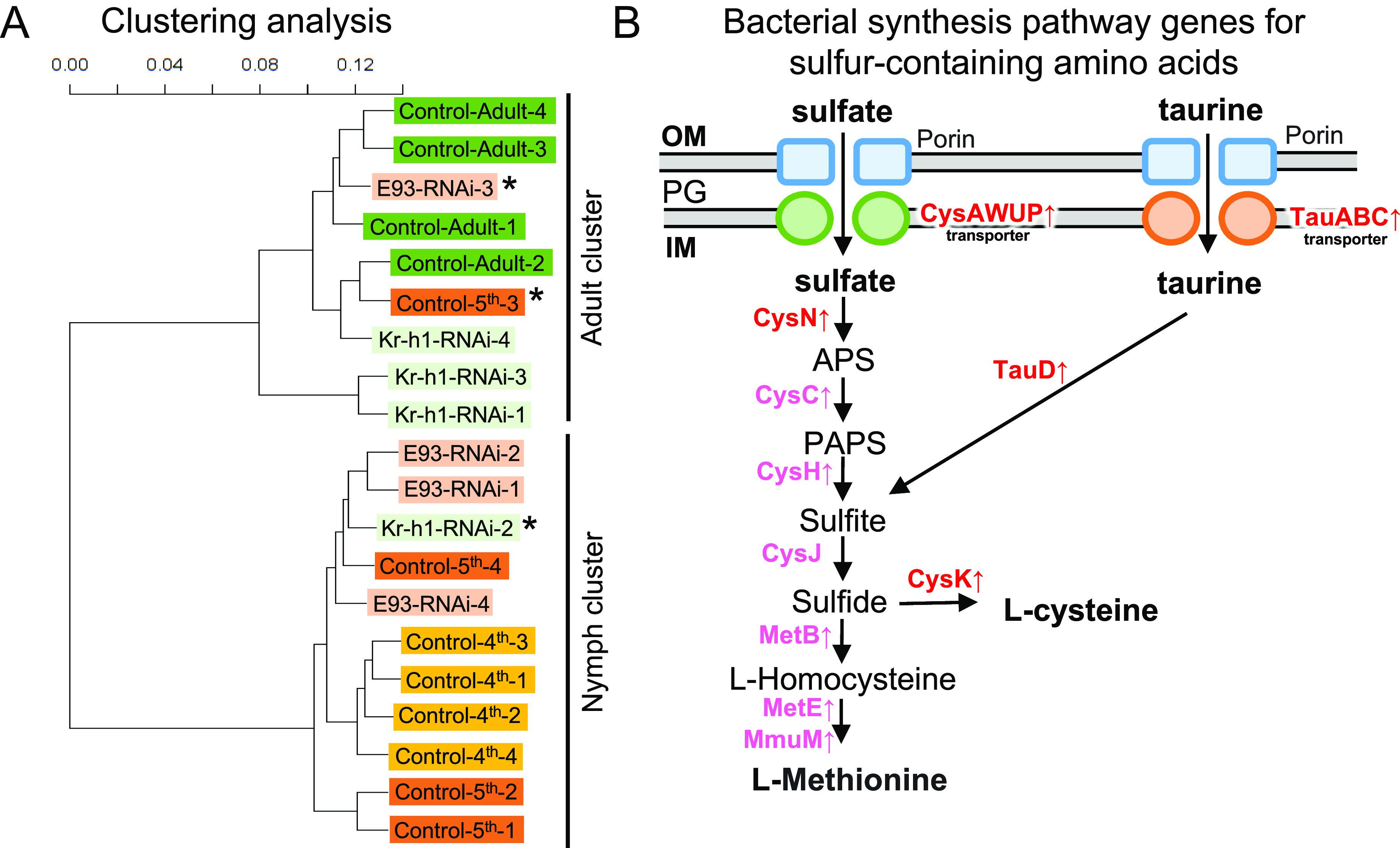
Gene expression patterns of the symbiotic bacteria in the developmental course of *P. stali*, and effects of RNAi knockdown of *E93* and *Kr-h1.* (*A*) Hierarchical clustering analysis using the symbiont-derived RNA sequencing reads (Fig. 5*B*). Note that the symbiotic bacteria of the *E93* RNAi supernumerary nymphs tend to cluster with the symbiotic bacteria of the control normal nymphs, forming the nymph cluster, whereas the symbiotic bacteria of the *Kr-h1* RNAi precocious adults tend to cluster with the symbiotic bacteria of the control normal adults, constituting the adult cluster, although there are some exceptions (highlighted by asterisks). (*B*) Synthetic pathways of sulfur-containing amino acids up-regulated in the symbiotic bacteria of the control normal adults. Red shows the genes whose expression level was significantly higher in adults than in 5^th^ instar nymphs. Pink shows the genes whose average expression level was higher in adults than in 5^th^ instar nymphs, although the difference was statistically not significant. Abbreviations: APS, adenylyl-sulfate/adenosine-5′-phosphosulfate; IM, inner membrane; PAPS, 3′-phosphoadenosine-5′-phosphosulfate; PG, peptidoglycan; OM, outer membrane. Also see SI *Appendix*, Fig. S10 and Table S9.

In an attempt to test the hypothesis that the symbiotic bacteria may be also under the regulation of host metamorphosis, the RNA sequencing reads of the symbiotic bacteria were extracted and analyzed from the control 4^th^ instar nymphs, the control 5^th^ instar nymphs, the control adults, the *E93* RNAi supernumerary nymphs, and the *Kr-h1* RNAi precocious adults (Fig. 5 *A* and *B*). The symbiont-derived reads were mapped to 4,352 genes predicted for the genome of the *Pantoea* sp. A symbiont (26). Hierarchical clustering analysis of the expressed symbiont genes revealed that the symbiotic bacteria of the *E93* RNAi supernumerary nymphs tended to cluster with those of the control normal nymphs, whereas the symbiotic bacteria of the *Kr-h1* RNAi precocious adults tended to group with the control normal adults, although there were exceptional cases: a control 5^th^ instar nymph and an *E93* RNAi supernumerary nymph were placed in the Adult cluster, and a *Kr-h1* RNAi precocious adult was in the Nymph cluster (Fig. 7*A*). These results seemed to support the hypothesis that the symbiotic bacteria of the supernumerary nymphs and the precocious adults are nymph-like and adult-like, respectively, in physiological/metabolic conditions.

### Up-regulated Symbiont Genes for Sulfur-containing Amino Acids in the Adult-type Symbiotic Organ.

From the symbiont-derived transcriptomic data, we extracted 49 differentially expressed genes between the control 5^th^ instar nymphs and the control adults (FDR *q* < 0.01), of which 4 symbiont genes were up-regulated in the nymphs (*SI Appendix*, Table S8), and 45 symbiont genes were up-regulated in the adults (*SI Appendix*, Table S9). While the nymph-up-regulated symbiont genes were inconspicuous both in number and expression levels, the adult-up-regulated symbiont genes exhibited a conspicuous pattern dominated by genes related to the synthesis of sulfur-containing amino acids. GO analysis of the adult-up-regulated symbiont genes identified a predominant representation of such GO terms as sulfur compound transport (0034728 and 1901682) and sulfate transport (0008272, 0015116, and 0015419) (*SI Appendix*, Fig. S9). The high-ranked annotated adult-up-regulated symbiont genes were represented by sulfate transporter components (*cysP*, *cysA*, *cysU*, and *cysW*; rank 10, 11, 25, and 27), sulfate adenylyltransferase components that convert imported sulfate to adenosine 5′-phosphosulfate (*cysD* and *cysN*; rank 9 and 16), taurine transporter components (*tauA*, *tauB*, and *tauC*; rank 5, 17, and 18), taurine dioxygenase that catalyzes oxygenolytic release of sulfite from taurine (*tauD*; rank 6), cysteine synthase that introduces sulfide into O-acetyl-L-serine to form L-cysteine (*cysK*, rank 3), and others (Fig. 7*B* and *SI Appendix*, Fig. S10 and Table S9). Most of the other synthetic pathway genes for the sulfur-containing amino acids L-cysteine and L-methionine, namely *cysC*, *cysH*, *cysJ*, *metB*, *metE*, and *mmuM*, exhibited higher expression levels in the adults than in the nymphs, although the differences were not significant statistically (FDR *q* > 0.01) (Fig. 7*B* and *SI Appendix*, Fig. S10). Notably, upregulation of these genes tended to be observed in the *Kr-h1* RNAi precocious adults as well as in the *E93* RNAi supernumerary nymphs (*SI Appendix*, Fig. S10 and Table S9).

### Possible Biological Relevance of Up-regulated Symbiont Genes for Sulfur-containing Amino Acids in Adult Symbiotic Organ.

**Fig. 8.**
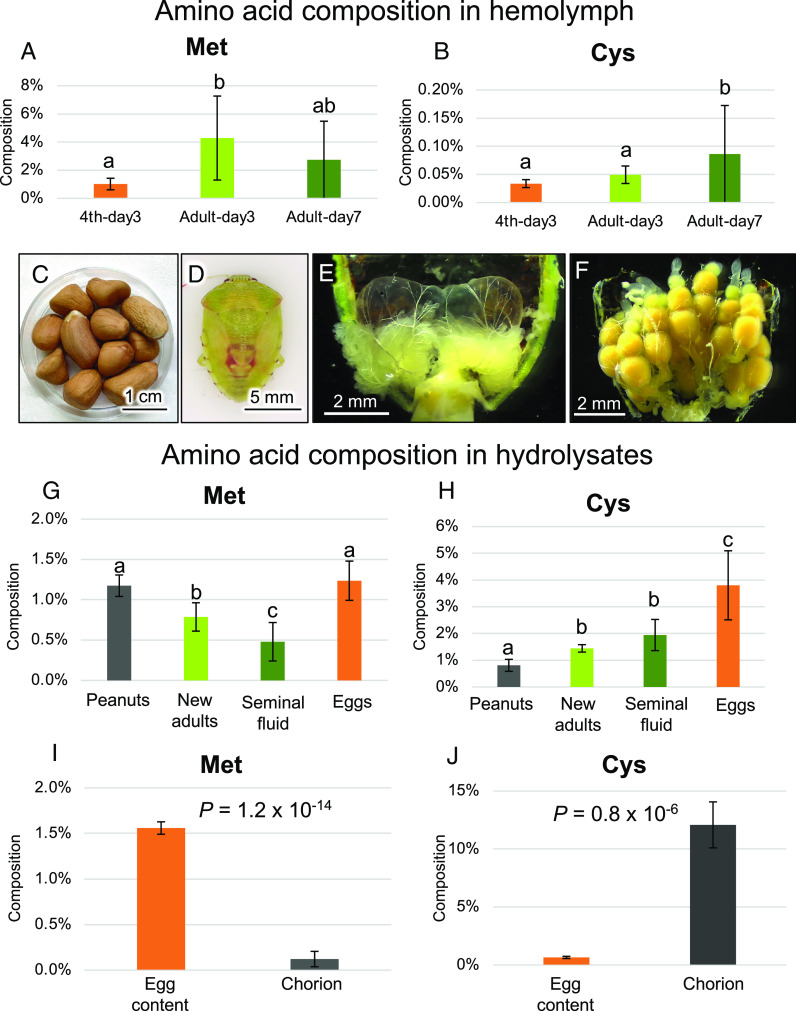
Abundance of sulfur-containing amino acids in the hemolymph, dissected tissues, and eggs of *P. stali*. (*A*) Comparison of the percentage of methionine in the hemolymph to total amino acids between 4^th^ instar nymphs 3 d after molting, adults 3 d after metamorphosis, and adults 7 d after metamorphosis. (*B*) Comparison of the percentage of cysteine in the hemolymph to total amino acids between 4^th^ instar nymphs 3 d after molting, adults 3 d after metamorphosis, and adults 7 d after metamorphosis. (*C*) Peanuts. (*D*) New adult insect just after metamorphosis. (*E*) Seminal vesicle in the male abdomen. (*F*) Eggs in the female abdomen. (*G*) Comparison of the percentage of methionine in the hydrolysate to total amino acids between peanuts, whole new adults, seminal fluids, and eggs. (*H*) Comparison of the percentage of cysteine in the hydrolysate to total amino acids between peanuts, whole new adults, seminal fluids, and eggs. In (*A, B, G*, and *H*), the samples were compared by Welch’s *t* test with Bonferroni’s correction (*P <* 0.05). (*I*) Comparison of the percentage of methionine in the hydrolysate to total amino acids between the egg content and chorion. (*J*) Comparison of the percentage of cysteine in the hydrolysate to total amino acids between the egg content and chorion. In (*I* and *J*), the samples were compared by Welch’s *t* test.

These gene expression patterns suggested the possibility that symbiont genes related to the synthesis of sulfur-containing amino acids are up-regulated for functioning in the adult symbiotic organ of *P. stali*. Hemolymph analysis revealed that i) the sulfur-containing amino acids, methionine and cysteine, were among the most scarce amino acids, and ii) adult hemolymph tended to contain more methionine and cysteine than nymphal hemolymph (Fig. 8 *A* and *B* and *SI Appendix*, Fig. S11*A*). These results are in favor of the notion that the symbiotic bacteria synthesize the limited sulfur-containing essential amino acids, methionine and cysteine, more actively in adults than in nymphs. Then, why preferentially in adults? Considering that adult insects generally invest much in mating and reproduction (31), we suspected that the sulfur-containing amino acids may be important for reproductive activities of the adult insects. To test this hypothesis, we analyzed the hydrolyzed amino acid composition of seminal fluid and eggs dissected from adult insects in comparison with the hydrolyzed amino acid composition of food peanuts and new adults (Fig. 8 *C*–*F*). While methionine contents were relatively higher in food peanuts and eggs (Fig. 8*G* and *SI Appendix*, Fig. S11*B*), notably, cysteine contents were significantly higher in seminal fluid and, in particular, eggs than in food peanuts (Fig. 8*H* and *SI Appendix*, Fig. S11*B*). Considering the relative abundance of cysteine compared to methionine in the hydrolysates (*SI Appendix*, Fig. S11*B*), it was suggested that the up-regulated cysteine synthesis by the symbiotic bacteria at the adult stage may be involved in adult reproduction, especially egg production. When egg content and chorion were separated, hydrolyzed, and analyzed for amino acid compositions, strikingly, cysteine was highly enriched in the chorion, accounting for as high as 12% of total amino acids, whereas cysteine occupied less than 1% of total amino acids of egg content (Fig. 8 *I* and *J* and *SI Appendix*, Fig. S11*C*). These results uncovered that the conspicuously higher cysteine content in eggs is attributable to their chorion, which strongly suggested that the upregulation of the symbiont genes related to sulfur-containing amino acids in response to metamorphosis is mainly for egg production by vigorously reproducing adult females of *P. stali*. In a previous study on the bean bug *Riptortus pedestris*, it was reported that the *Burkholderia* gut symbiont increases the expression levels of genes related to sulfur metabolism within the host compared to in culture (32). It was also reported that the aphid endosymbiont *Buchnera* converts inorganic sulfate from phloem sap to cysteine for the host aphid (33, 34). Taken together, it is suggested that the up-regulated sulfur metabolism leading to the synthesis of cysteine and methionine may be generally important for the hemipteran insects and their symbiotic bacteria.

### Conclusion and Perspective.

**Fig. 9.**
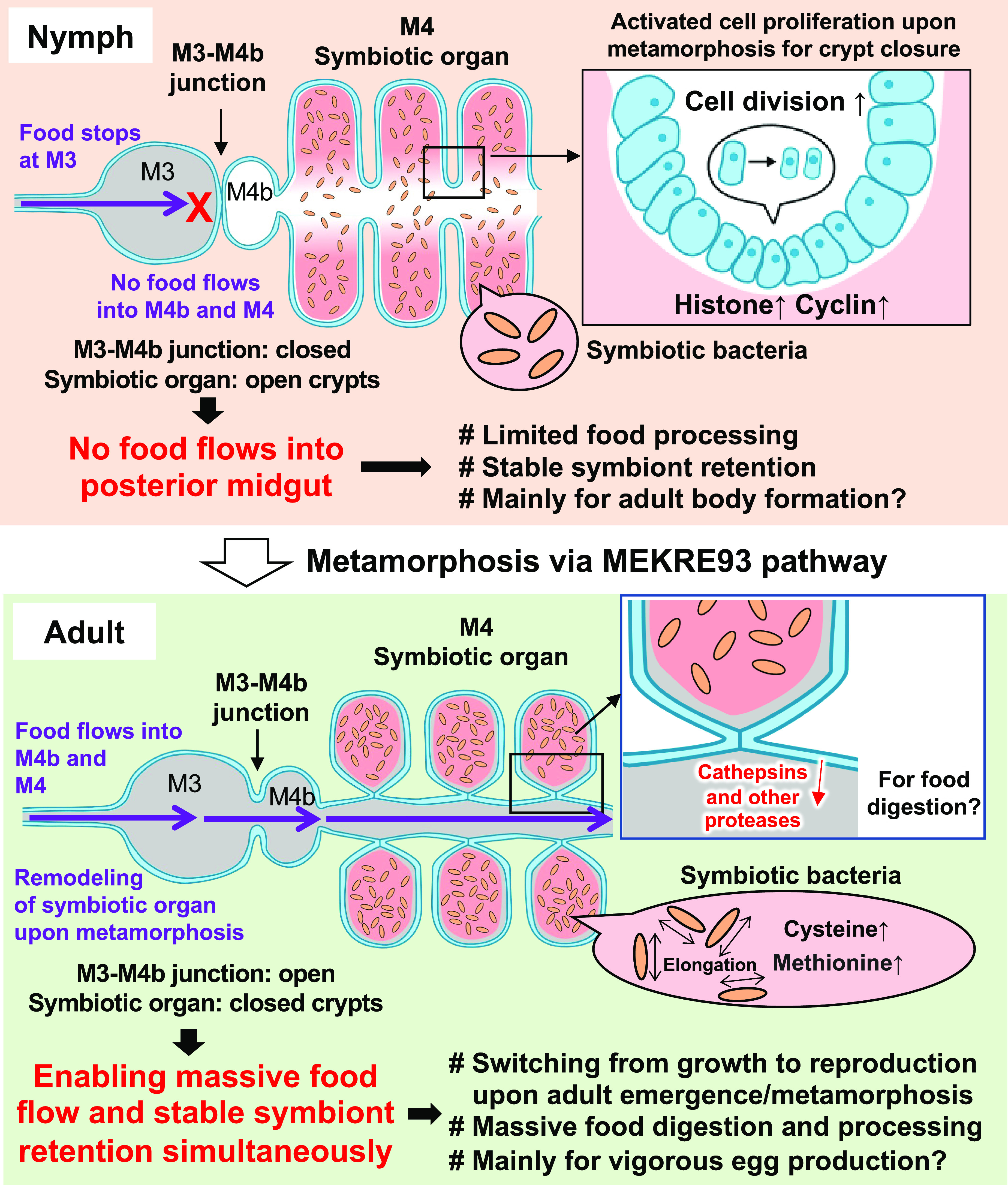
Schematic diagram of the summary of this study. Remodeling of the symbiotic organ occurs upon metamorphosis via the MEKRE93 pathway, which entails altered food passage, reorganized symbiont retention machineries, up-regulated host and symbiont genes, and possible functional and biological consequences.

In conclusion, we demonstrated that morphogenesis and functioning of the symbiotic organ are controlled in accordance with insect metamorphosis by the MEKRE93 pathway in *P. stali*, which highlight how intricately the microbial symbiosis is integrated into development, physiology, reproduction, and adaptation of the host insect. Furthermore, we found that morphology and functioning of the symbiotic bacteria within the symbiotic organ are also regulated in accordance with insect metamorphosis, which is probably under an indirect control of the MEKRE93 pathway via altered conditions of the symbiotic organ upon metamorphosis. Fig. 9 graphically summarizes the important findings uncovered in the present study. Morphologically, closed M3-M4b junction and open crypts in nymphs are transformed into open M3-M4b junction and closed crypts in adults, which enable functional extension of the posterior midgut region from symbiosis only to food digestion, processing, and absorption in addition to symbiosis. Transcriptomically, the nymphal symbiotic organ consisting exclusively of the open crypts preferentially expresses the genes related to cell cycle and division, which presumably underpin the growth of the symbiotic organ during the nymphal period. Meanwhile, the adult symbiotic organ differentiating into the closed crypts and the main tract highly expresses the genes of proteolytic enzymes specifically in the main tract, which must reflect the massive feeding and vigorous reproduction of the adult insects of *P. stali*. Furthermore, in the closed crypts in the adult-type symbiotic organ, the symbiotic bacteria up-regulate the genes for synthesis of sulfur-containing amino acids, which may be pivotal for massive egg production because much cysteine is needed for production of eggshell.

Future studies should focus on several important directions. The gene regulatory network that connects the master regulator genes *E93* and *Kr-h1* to the downstream-regulated genes responsible for the symbiosis-related phenotypes is to be identified. Being upstream of *E93* and *Kr-h1* via the receptors Met and EcR, it is of interest how the principal insect hormones, JH and ecdysteroid, control the symbiotic organ and the symbiotic bacteria therein. It is also of great interest what factors in the adult-type symbiotic organ affect the symbiotic bacteria to regulate the bacterial metabolism toward the production of sulfur-containing amino acids, methionine, and cysteine.

## Materials and Methods

In this study, we used a mass-reared laboratory strain of *P. stali*, which had been established from adult insects collected at Tsukuba, Ibaraki, Japan. The insects were fed with raw peanuts and distilled water supplemented with 0.05% ascorbic acid. We inspected the hatching or molting of the insects every day between 12:00 and 17:00 and renewed food and drinking water once a week. The insects were kept in climatic chambers at 25 ± 1 °C under a long-day regime of 16 h light and 8 h dark. For RNA sequencing, the midgut symbiotic organs dissected from these insects were individually subjected to RNA extraction, cDNA library construction, and DNA sequencing using HiSeq4000 (Illumina, USA). Raw Fastq data have been deposited in the DNA Data Bank of Japan Read Archive under the accession numbers DRR446133–DRR446184 (*SI Appendix*, Table S10). Complete details on the materials and methods are available in *SI Appendix*.

## Supplementary Material

Appendix 01 (PDF)

## Data Availability

Raw FASTQ data have been deposited in DNA Data Bank of Japan with an accession number PRJDB15321 (35) (including DRR446133–DRR446184; see *SI Appendix*, Table S10 for detail).
