# Supplementary material for: Regulation and remodeling of microbial symbiosis in insect metamorphosis
Source: Proc Natl Acad Sci U S A. 2023 Sep 28;120(40):e2304879120. doi: 10.1073/pnas.2304879120 (PMC10556603; doi:10.1073/pnas.2304879120)
Supplement: Supplementary file 1 — Appendix 01 (PDF) [file pnas.2304879120.sapp.pdf]

PNAS

Supporting Information for

Regulation and remodeling of microbial symbiosis in insect metamorphosis

Sayumi Oishi, Minoru Moriyama, Masaki Mizutani, Ryo Futahashi, Takema Fukatsu

Corresponding authors Minoru Moriyama and Takema Fukatsu
Email: m-moriyama@aist.go.jp, t-fukatsu@aist.go.jp

This PDF file includes:

SI Materials and Methods
SI References
Figures S1 to S11
Tables S1 to S10

SI Materials and Methods

Insect Material. In this study, we used a mass-reared laboratory strain of *P. stali*, which had been established from adult insects collected at Tsukuba, Ibaraki, Japan. *P. stali* populations living in the mainland Japan are obligatorily associated with an uncultivable γ-proteobacterium, which is closely related to *Pantoea dispersa* and called *Pantoea* sp. A or symbiont A, in the midgut symbiotic organ (1). Laboratory rearing of the insects was conducted as described previously (2). The insects were fed with raw peanuts and distilled water supplemented with 0.05% ascorbic acid. We inspected hatching or molting of the insects everyday between 12:00 and 17:00 and renewed food and drinking water once a week. The insects were kept in climatic chambers at 25 ± 1°C under a long-day regime of 16 h light and 8 h dark.

Obtaining *E93* and *Kr-h1* Gene Sequences and dsRNA Synthesis. *E93* and *Kr-h1* gene sequences of *P. stali* were obtained from previous RNA sequencing data (3). The ORF sequences were predicted using the ORF finder (https://www.ncbi.nlm.nih.gov/orffinder/) from the contig sequences. The primers for RNAi and quantitative PCR were designed using primer3 (https://bioinfo.ut.ee/primer3-0.4.0/). For RNA extraction, the dissected insect tissues were crushed in RNAiso Plus (Takara, Japan) and subjected to chloroform extraction. The supernatant was mixed with an equal amount of 70% ethanol and subjected to RNA purification using RNeasy Mini Kit (Qiagen, Germany). After ribosomal RNAs were removed from the obtained RNA samples using RiboPOOL (siTOOLs Biotech, Germany), cDNA libraries were constructed using TruSeq RNA Sample Preparation Kits V2 (Illumina, USA). The target gene sequences were amplified by PCR using TaKaRa Ex Taq (Takara, Japan) with the following primers designed for RNAi: for *Kr-h1*, Ps_krh1_dsRNA_F [5'-TCT ACC GGT GGT GTT TCA GC-3'] and Ps_krh1_dsRNA_R [5'-CTG TGT TCA AAG GCA CGC TC-3']; for *E93*, pstali_e93_F [5'-CAG AAG AAG AAC GGG AGA CG-3'] and pstali_e93_R [5'-CTT GCC TCT TTT CGG TCT TG-3']. The amplified products were cloned using T-Vector pMD20 (Takara, Japan) and *Escherichia coli* DH5a competent cells. Colony PCR amplification was performed using a primer in which the T7 promoter region sequence was bound to the 5' end to a sequence complementary to both ends of the vector. The obtained products were purified using QIAquick PCR Purification Kit (QIAGEN, Germany), from which dsRNAs were synthesized using MEGAscript RNAi Kit (Invitrogen, USA). The concentration of dsRNA was measured using Nano drop.

Injection of dsRNA. Synthesized dsRNA was injected into nymphs of *P. stali* on the day of 4th instar molting or on the day of 5th instar molting (see Fig. 2*A*). Injection was performed using pulled glass needles made of 1-5 μl graduated glass capillary tubes (Drummond, USA). The intersegmental membrane between head and thorax was pierced and injected with 200 ng/μL of dsRNA solution, which was 0.25 μL per insect for 4th instar nymphs and 0.5 μL per insect for 5th instar nymphs. As a control, dsRNA targeting β-lactamase was prepared and injected in the same manner.

Morphometry of Symbiotic Organ. The insects were dissected under a stereomicroscope (SZ61, Olympus, Japan) in a phosphate buffered saline (PBS: 137 mM NaCl, 8.1 mM Na_2HPO_4, 2.7 mM KCl, 1.5 mM KH_2PO_4 [pH 7.4]) to isolate their alimentary tract. The dissected alimentary tract was unfolded and photographed with a microscope camera (WRAYCAM-NOA630B, WRAYMER, Japan), and the length of the symbiotic organ was determined using ImageJ v1.53 (https://imagej.nih.gov/ij/).

Histological Observation of Symbiotic Organ and Symbiotic Bacteria. The dissected

symbiotic organs were fixed with 4% paraformaldehyde in PBS, soaked in 6.8% sucrose solution, and embedded in Technovit 8100 methacrylate resin (Heraeus Kulzer, Germany). The embedded samples were trimmed with an electric cutter and processed into 2 µm sections using a microtome (RM2255, Leica, Germany). The tissue sections were mounted on glass slides and subjected to fluorescence in situ hybridization (FISH) or toluidine blue staining. FISH was conducted with a fluorescent oligonucleotide probe SymAC89R (5'-Alexa555- GCA AGC TCT TCT GTG CTG CC -3') specifically targeting 16S rRNA of the symbiotic bacteria (2, 4). The tissue sections were incubated with a hybridization buffer (20 mM Tris-HCl [pH 8.0], 0.9 M sodium chloride, 0.01% sodium lauryl sulfate, 30% formamide, 1 µg/mL 4', 6-diamidino-2-phenylindole dihydrochloride [DAPI]) supplemented with 100 nM probe for 2 h in a humidified dark box. After washing with PBST (PBS containing 0.1% Tween 20), the tissue sections were sealed with 80% glycerol and observed under a fluorescence microscope (LSM700, Zeiss, Germany).

Cytometry of Symbiotic Bacteria. Each symbiotic organ dissected from an insect was homogenized in 100 µL of PBS. The homogenate was observed under a phase-contrast microscope (IX71, Olympus, Japan) equipped with a charge-coupled device camera (DMK33UP5000.WG; The Imaging Source, Germany). The bacterial cell images were captured and analyzed using ImageJ v1.53 (https://imagej.nih.gov/ij/). For each treatment group, 50 bacterial cells from each of four symbiotic organs dissected from four insects were subjected to the analysis.

RNA Sequencing Analysis. The following insects were subjected to a series of transcriptomic analyses of the symbiotic organ and the symbiotic bacteria therein: four control 4th instar nymphs 3 days after molting (sexing difficult); four control 5th instar nymphs 3 days after molting (two females and two males); four control 6th instar adults 3 days after molting (two females and two males); four *E93* RNAi 6th instar supernumerary nymphs 3 days after molting (two females and two males); and four *Kr-h1* RNAi 5th instar precocious adults 3 days after molting (one female and three males) (see Fig. 5*A*). The midgut symbiotic organs dissected from these insects were individually subjected to RNA extraction, cDNA library construction, and DNA sequencing using HiSeq4000 (Illumina, USA). After pre-processing of the sequences by Trimmomatic v0.36 (5), we extracted host-derived cDNA reads and symbiont-derived cDNA reads by serial mapping to the symbiont genome and then to host rRNA sequences (see Fig. 5*B*). Then, de novo assembling was performed using Trinity v2.8.5 (https://github.com/trinityrnaseq/trinityrnaseq/releases/tag/Trinity-v2.5.1). The obtained contig sequences were annotated using Refseq of the stinkbug *Halymorpha halys* (NCBI). Expression levels of the contigs were estimated using Salmon v0.14.1. Gene ontology (GO) analysis was performed using the functional annotation tool DAVID (http://david.abcc.ncifcrf.gov69). Differentially expressed genes (DEGs) were identified using edgeR v.3.16.5 (6, 7) in RStudio v.1.2.5019 (https://posit.co/products/open-source/rstudio) under the criteria of FDR < 0.01 and higher TPM value being no less than 2.

Cytological Visualization of Proliferating and Dividing Cells. We performed cell proliferation analysis of the symbiotic organ using 5-ethynyl-2'-deoxyuridine (EdU), a thymidine analog that is incorporated into newly synthesized DNA, and phosphorylated Histone H3 (H3P) antibody that binds to dividing cells specifically. The insects were injected with 50 nL of 10 µM EdU solution, and 1 h after injection, the midgut symbiotic organs were dissected and fixed with PFA buffer for 1 h. The fixed tissue samples were subjected to florescence labeling of EdU using Click-iT EdU Imaging Kits Alexa Flour 647 (Invitrogen, USA) for 30 min. Subsequently, the tissue samples were treated with blocking buffer (PBS

supplemented with 1% bovine serum albumin), incubated with anti-Histone H3S10ph antibody (rabbit, polyclonal, GeneTex, USA) for 30 min, and incubated with anti-rabbit florescent antibody (goat, monoclonal, Alexa Flour 555) for 30 min. After thorough washing with PBS, the tissue samples were counter-stained with PBS supplemented with 1 μg/mL DAPI, and observed under a laser confocal microscope (LSM700, Zeiss, Germany).

Amino Acid Quantification. Hemolymph was collected from the neck of day-3 4th instar nymphs, day-3 adults and day-7 adults. After piercing the intersegment membrane using a fine needle, 1 μL of hemolymph was collected and combined with 499 μL of 80% methanol using a glass capillary (Microcap, Drummond, USA). The samples were stored at -80°C until analysis. For peanuts, newly emerged adults, sperm and eggs, we measured amino acids after protein hydrolysis. For peanuts, approximately 100 mg of crushed peanuts were deoiled with 500 μL hexane. After that, the peanuts were pulverized using a mortar and pestle, and around 30 mg was weighed. For sperm, one seminal vesicle dissected from an adult male was collected per sample. For eggs, 10 eggs were collected from ovarioles of an adult female per sample. All the samples were weighed and homogenized using a bead homogenizer (μT-12, TAITEC, Japan). The samples were resuspended to 500 μL of water. For each sample, 50 μL was transferred to a new tube, combined with 450 μL of acetone, and cooled at -20°C for 30 min to precipitate proteins. After that, the samples were centrifuged at 12,000 rpm for 5 min at 4°C, from which the supernatant was discarded. After the pellet was dried, 200 μL of hydrolysis solution (4N methanesulfonic acid containing 0.2% tryptamine) was added and the air was replaced by N_2. The samples were hydrolyzed at 110°C for 24 h. After hydrolysis, the samples were neutralized by adding 400 μL of 2N NaOH and centrifuged at 12,000 rpm for 5 min at 4°C. For each sample, 10μL of the supernatant was transferred to a new tube, dried by a micro centrifugal vacuum concentrator (MV-100, TOMY, Japan), and kept at -80°C until analysis. Amino acid composition of the samples was analyzed by mass spectrometry after chemical derivatization as previously described (3). Each of the hemolymph samples and the acid-hydrolyzed samples was mixed with an arbitrary amount of homoarginine and homophenylalanine as internal standards, and subjected to an alkylation procedure using propylchlorformate (8). The derivatized amino acids were extracted with chloroform, dried under a nitrogen stream, and resuspended to 50% methanol. The samples were analyzed with an ultra-high performance liquid chromatograph (Acquity UPLC H-class, Waters, USA) and a quadrupole time-of-flight mass spectrometer (Xevo G2-XS, Waters, USA) equipped with an electrospray ionization source. An octadecylsilyl column (100 mm x 2 mm i.d., CORTECS T3, Waters, USA) was used with a gradient-elution program of mobile phase A (water containing 0.05% formic acid and 2.5 mM ammonium formate) and B (methanol). The concentration of B was increased from 40% to 95% in 6 min at a flow rate of 0.3 mL/min. Protonated ions [M + H]+ of derivatized amino acids were monitored in a positive ESI mode, and quantification process was operated using Masslynx software (Waters, USA).

SI References

1. Hosokawa T, Ishii Y, Nikoh N, Fujie M, Satoh N, Fukatsu T (2016) Obligate bacterial mutualists evolving from environmental bacteria in natural insect populations. Nature Microbiology 1, 15011. https://doi.org/10.1038/nmicrobiol.2015.11
2. Oishi S, Moriyama M, Koga R, Fukatsu T (2019) Morphogenesis and development of midgut symbiotic organ of the stinkbug *Plautia stali* (Hemiptera: Pentatomidae). Zoological Letters 5, 16. https://doi.org/10.1186/s40851-019-0134-2
3. Moriyama M, Hayashi T, Fukatsu T (2022) A mucin protein predominantly expressed in the female-specific symbiotic organ of the stinkbug *Plautia stali*. Scientific Reports 12, 7782. https://doi.org/10.1038/s41598-022-11895-1
4. Koga R, Tsuchida T, Fukatsu T (2009) Quenching autofluorescence of insect tissues for *in situ* detection of endosymbionts. Applied Entomology and Zoology 44(2), 281–291. https://doi.org/10.1303/aez.2009.281
5. Bolger AM, Lohse M, Usadel B (2014) Trimmomatic: a flexible trimmer for Illumina sequence data. Bioinformatics 30(15), 2114-2120. http://doi.org/10.1093/bioinformatics/btu170
6. Robinson MD, McCarthy DJ, Smyth GK (2010) edgeR: a Bioconductor package for differential expression analysis of digital gene expression data. Bioinformatics 26(1), 139–140. https://doi.org/10.1093/bioinformatics/btp616
7. McCarthy DJ, Chen Y, Smyth GK (2012) Differential expression analysis of multifactor RNA-Seq experiments with respect to biological variation. Nucleic Acids Research 40(10), 4288–4297. https://doi.org/10.1093/nar/gks042
8. Uutela P, Reinilä R, Harju K, Piepponen P, Ketola RA, Kostiainen R (2009) Analysis of intact glucuronides and sulfates of serotonin, dopamine, and their phase I metabolites in rat brain microdialysates by liquid chromatography-tandem mass spectrometry. Analytical Chemistry 81(20), 8417–8425. https://doi.org/10.1021/ac901320z

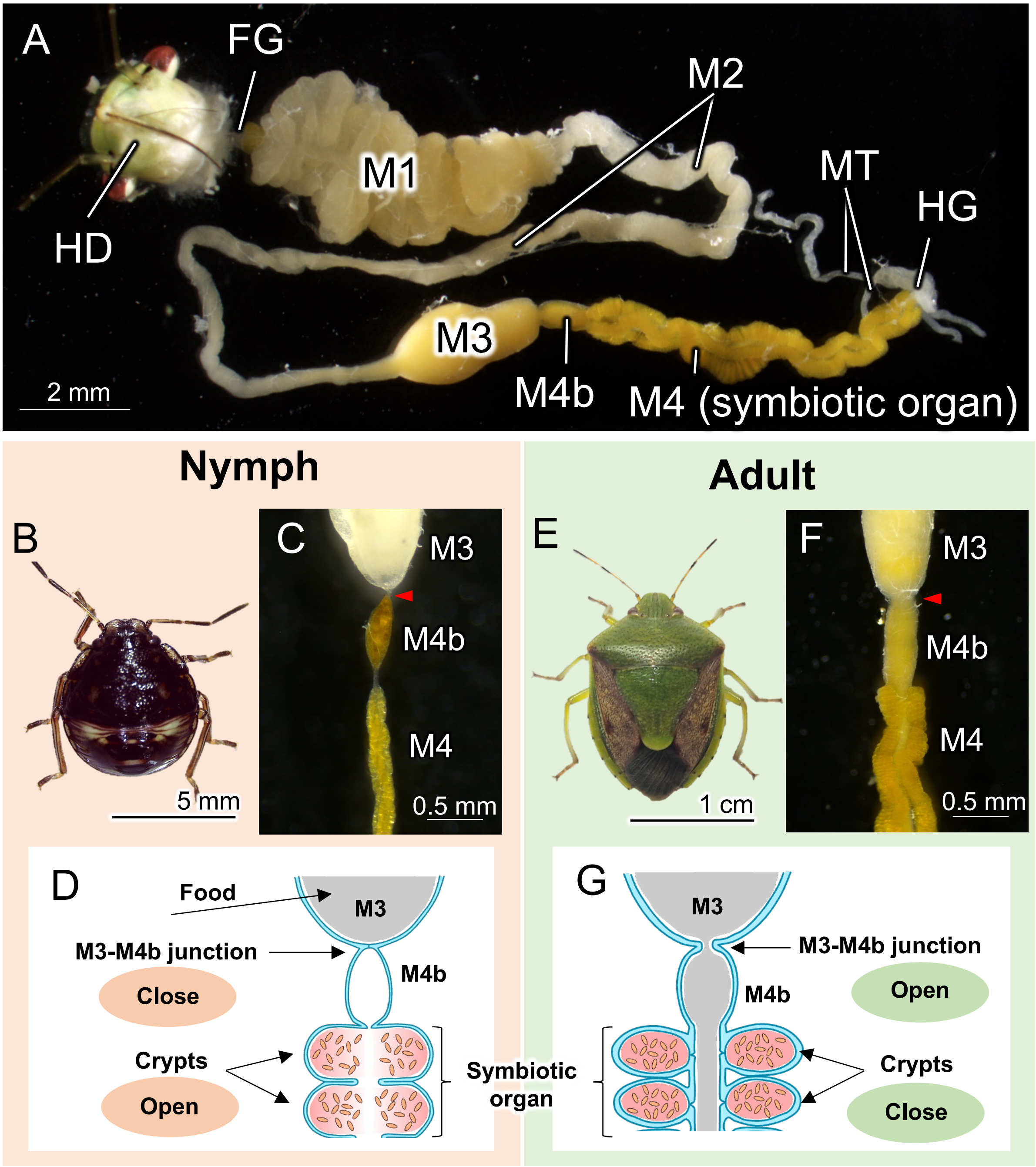

Fig. S1. Morphological changes of the symbiotic organ during metamorphosis of *P. stali*. (*A*) Dissected alimentary tract. Abbreviations: HD, head; FG, foregut; M1, M2 and M3, midgut M1, M2 and M3 regions; M4b, bulb-like midgut region anterior to M4; M4, symbiotic midgut M4 region with crypts arranged in four rows; HG, hindgut; MT, Malpighian tubule. (*B-D*) Fourth instar nymph. (*E-G*) Sixth instar adult. (*B, E*) External morphology. (*C, F*) M3-M4b junction of dissected alimentary tract. (*D, G*) Schematic illustration of M3, M4b and M4 regions of alimentary tract. In nymphs, the M3-M4b junction is closed, and thus the midgut M4b-M4 regions harboring the symbiotic bacteria are structurally isolated from the anterior midgut regions without food flow. In adults, by contrast, the M3-M4b junction is open, the base of each crypt is closed, and thus the symbiotic bacteria are isolated in the crypt cavities from the midgut main tract where food flows (2).

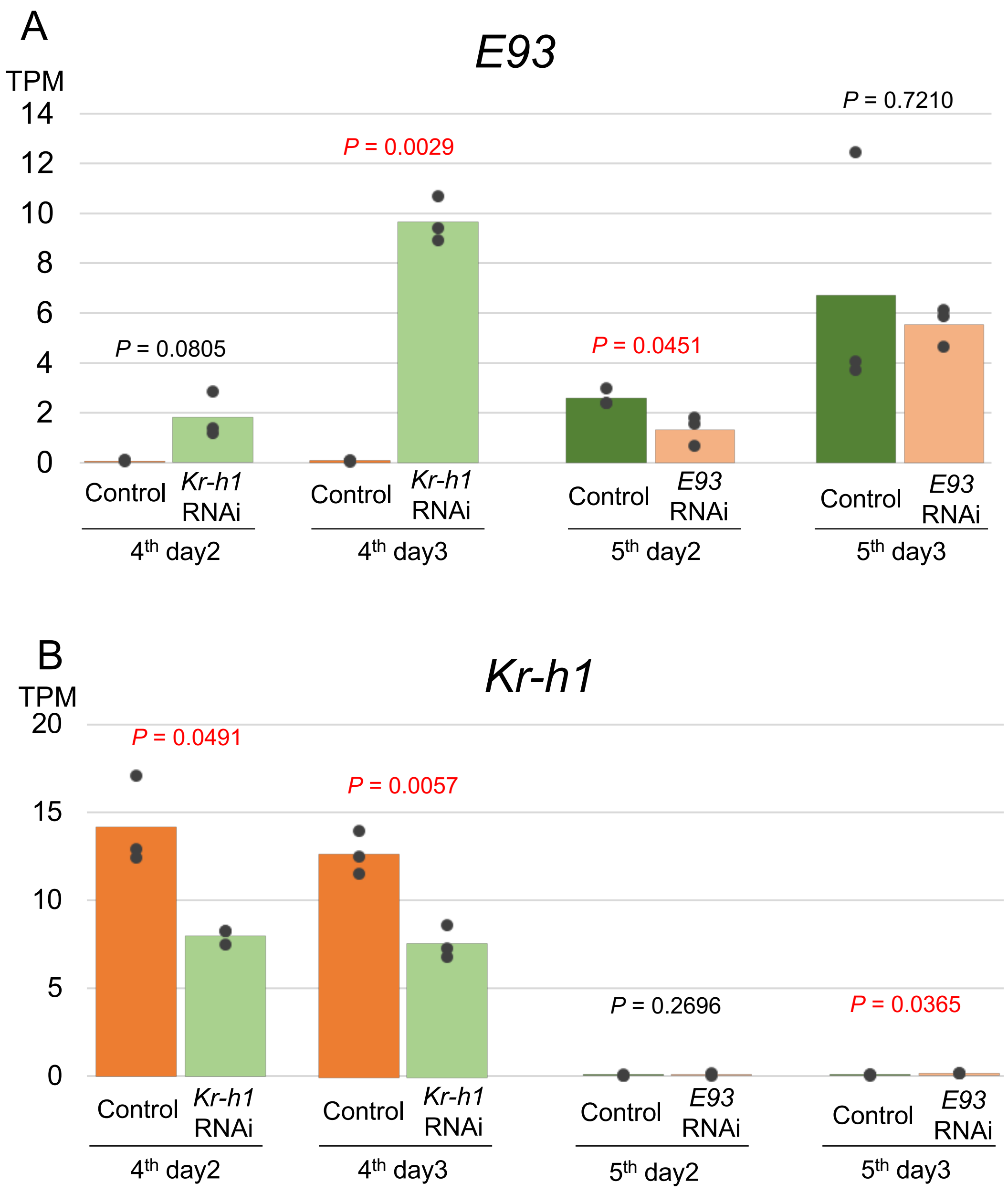

Fig. S2. Effect of RNAi knockdown of *E93* and *Kr-h1* in *P. stali*. (*A*) Expression levels of *E93* when subjected to *E93* or *Kr-h1* RNAi. (*B*) Expression levels of *Kr-h1* when subjected to *E93* or *Kr-h1* RNAi. The black dots represent the transcripts per million (TPM) of each individual, and the bar graph shows the average value of them. *P*-values of *t*-test are shown on the graphs.

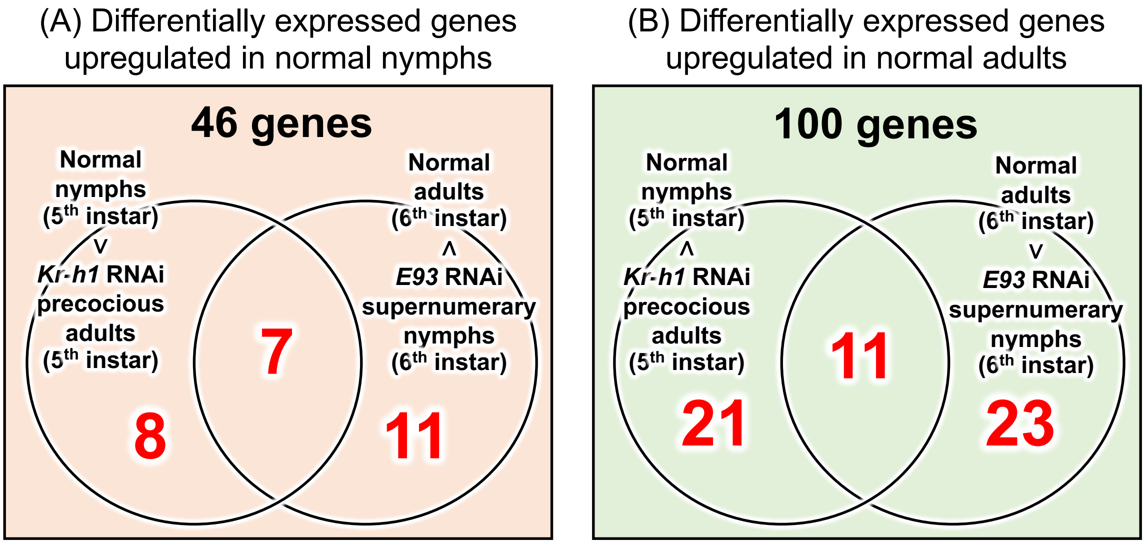

Fig. S3. Effects of RNAi knockdown of *Kr-h1* and *E93* on differentially expressed genes between nymphal and adult symbiotic organs of *P. stali*. (*A*) Of the 46 genes significantly upregulated in the normal 5th instar nymphs than in the normal adults (FDR $q < 0.01$), 15 genes exhibited significantly higher expression in the normal nymphs than in the *Kr-h1* RNAi precocious adults and 18 genes showed significantly higher expression in the *E93* RNAi supernumerary nymphs than in the normal adults (FDR $q < 0.01$), of which 7 genes were shared between them. (*B*) Of the 100 genes significantly upregulated in the normal adults than in the normal 5th instar nymphs (FDR $q < 0.01$), 32 genes exhibited significantly higher expression in the *Kr-h1* RNAi precocious adults than in the normal nymphs and 34 genes showed significantly higher expression in the normal adults than in the *E93* RNAi supernumerary nymphs (FDR $q < 0.01$), of which 11 genes were shared between them.

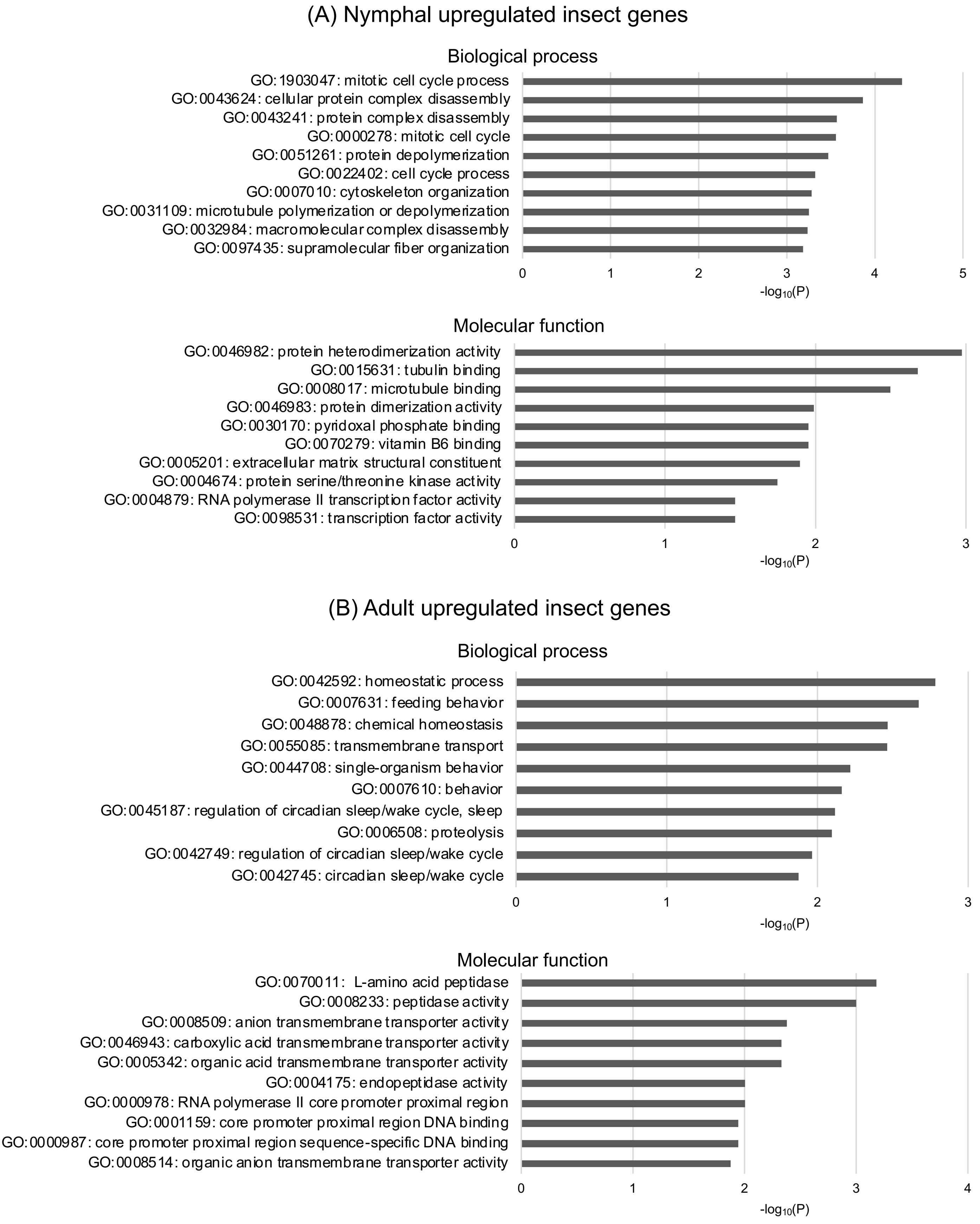

Fig. S4. GO analysis of differentially expressed genes between nymphs and adults of *P. stali*. (*A*) Nymph-upregulated insect genes. (*B*) Adult-upregulated insect genes.

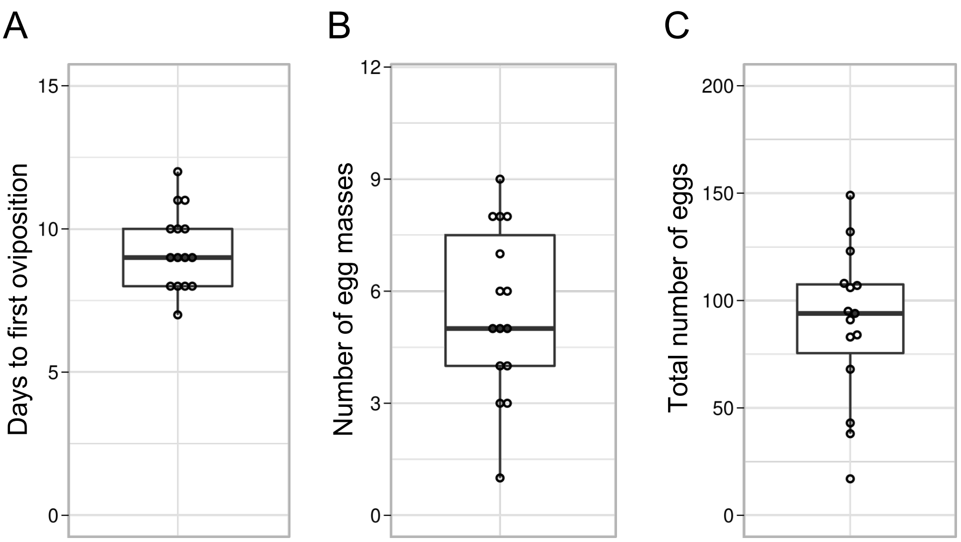

Fig. S5. Reproductive activity of *P. stali*. (*A*) Days to first oviposition after adult emergence. (*B*) Number of egg masses per female during 15 days after adult emergence. (*C*) Number of eggs per female during 15 days after adult emergence. In total 15 newly emerged adult pairs were fully fed and monitored for 15 days. Usually, this level of reproductive activity continues for over a month.

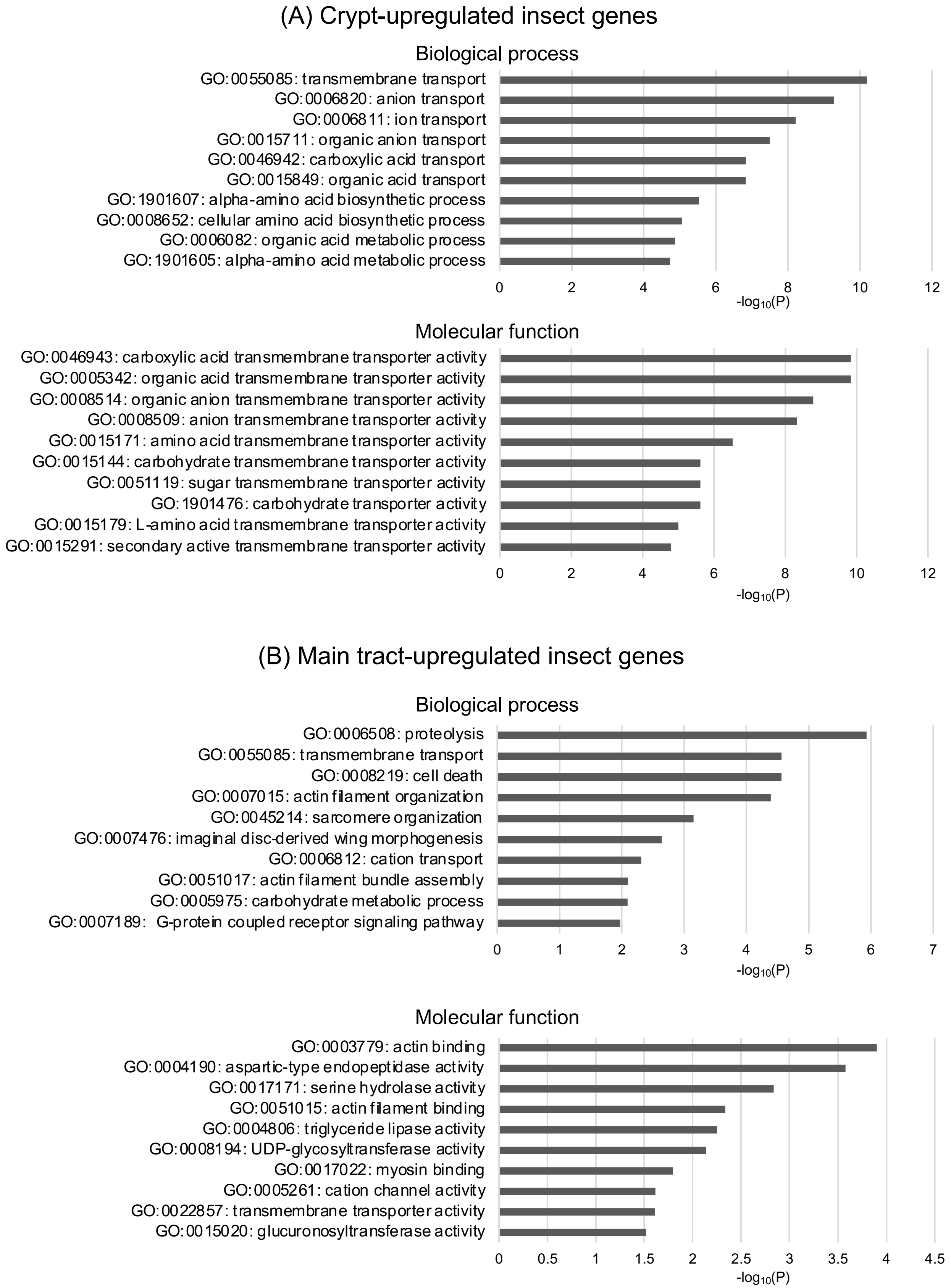

Fig. S6. GO analysis of differentially expressed genes between crypts and main tracts in the adult symbiotic organ of *P. stali*. (*A*) Crypt-upregulated insect genes. (*B*) Main tract-upregulated insect genes.

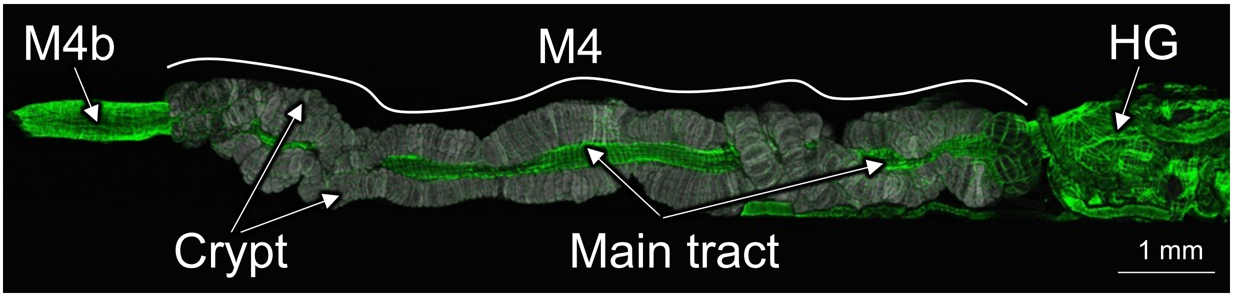

Fig. S7. Phalloidin staining of adult symbiotic organ of *P. stali*. Symbiotic bacteria (white) and actin fibers (green) are visualized by FISH and phalloidin staining, respectively. Abbreviations: M4b, bulb-like midgut region anterior to M4; M4, symbiotic midgut M4 region with crypts; HG, hindgut.

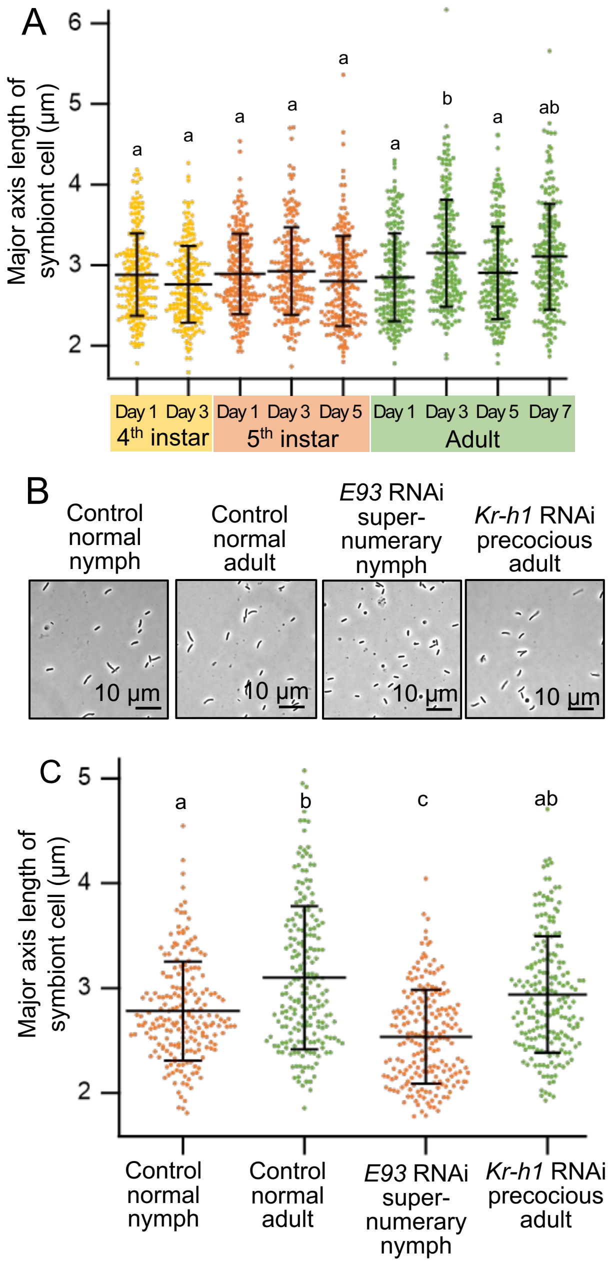

Fig. S8. Effects of RNAi knockdown of *E93* and *Kr-h1* on the symbiont morphology in the symbiotic organ of *P. stali*. (*A*) Length of the symbiotic bacteria in the symbiotic organ during the developmental course (from 4th instar through 5th instar to adult) of the control normal insects. (*B*) Phase-contrast microscopic images of the symbiotic bacteria in the symbiotic organ of a control nymph, a control adult, an *E93* RNAi supernumerary nymph, and a *Kr-h1* RNAi precocious adult. (*C*) Length of the symbiotic bacteria in the symbiotic organ of control nymphs, control adults, *E93* RNAi supernumerary nymphs, and *Kr-h1* RNAi precocious adults. In (*A*) and (*C*), length values of 50 bacterial cells from 4 insects are plotted with mean and standard deviation for each experimental group. Different alphabetical letters (a-c) indicate statistically significant differences (Steel-Dwass test, $P < 0.05$).

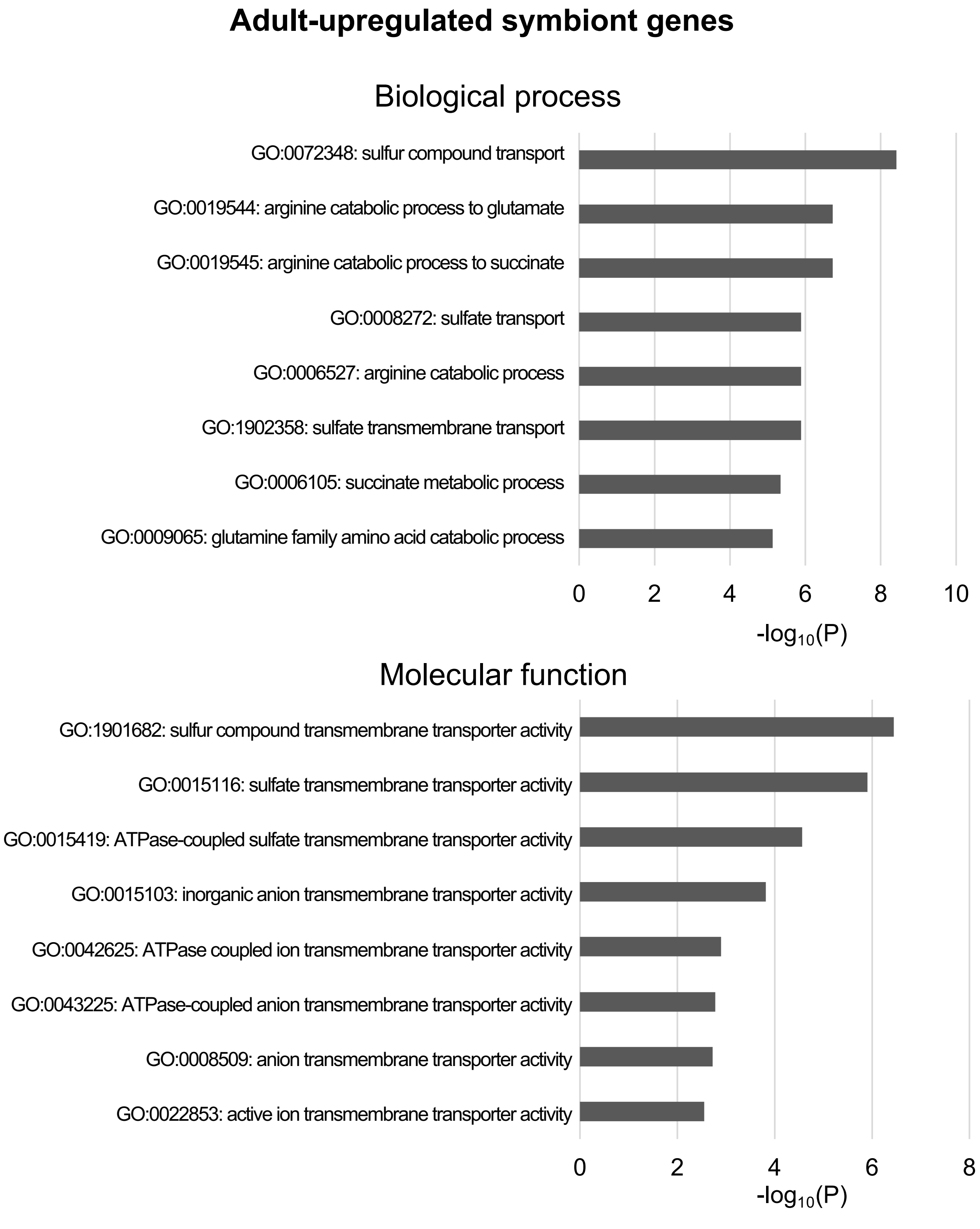

Fig. S9. GO analysis of adult-upregulated symbiont genes in the symbiotic organ of *P. stali*.

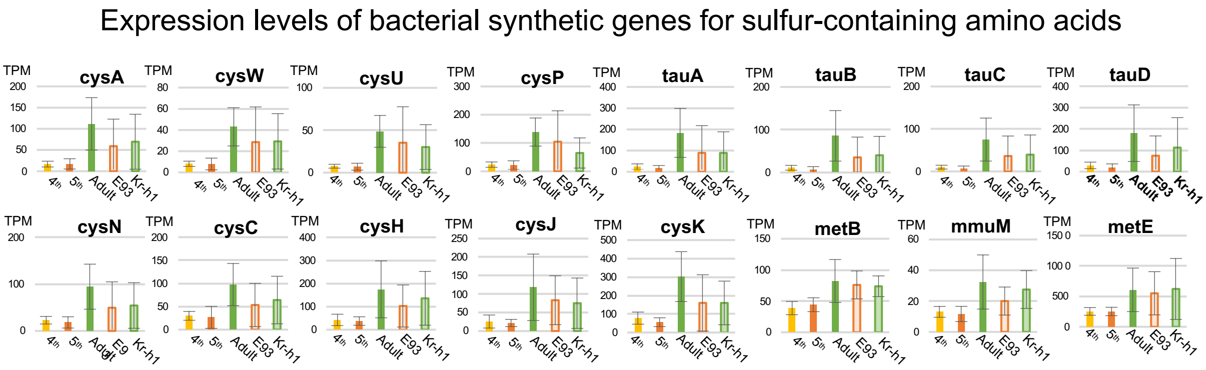

Fig. S10. Expression levels of the synthetic pathway genes of the symbiotic bacteria for sulfur-containing amino acids. Note that these genes are upregulated not only in the control normal adults but also in the *Kr-h1* RNAi precocious adults and also in the *E93* RNAi-induced supernumerary nymphs. Mean and standard deviation of 4 replicates are shown. Also see Fig. 7*B* and *SI Appendix*, Table S9.

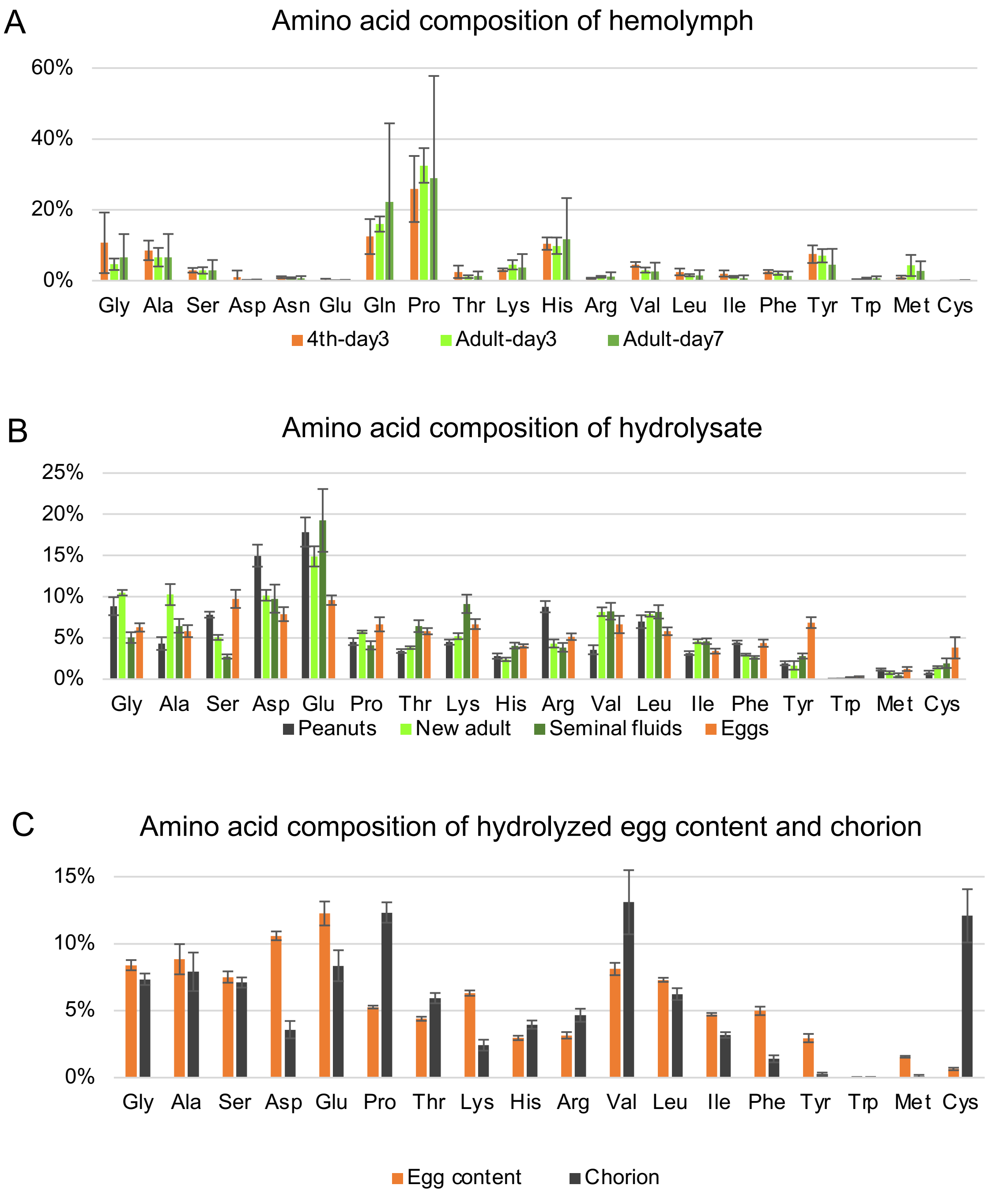

Fig. S11. Amino acid compositions of hemolymph, dissected tissues, and eggs of *P. stali*. (*A*) Comparison of the composition of amino acids in the hemolymph to total amino acids between 4th instar nymphs 3 days after molting, adults 3 days after metamorphosis and adults 7 days after metamorphosis. (*B*) Comparison of the percentage of the composition of amino acids in the hydrolysate to total amino acids between peanuts, whole new adults, seminal fluids and eggs. (*C*) Comparison of the percentage of the composition of amino acids in the hydrolysate to total amino acids between egg content and chorion.

Table S1. Effect of RNAi knockdown of *E93* in *P. stali*

dsRNA[1]	N	Death	Normal adult	Supernumerary nymph
E93	30	1	0	29
Control	20	0	20	0

[1]Double-stranded RNA preparations targeting *E93* gene (E93) and β-lactamase gene (Control) were injected into 5th instar nymphs, and their phenotypes were inspected upon adult (= 6th instar) molt.

Table S2. Effect of RNAi knockdown of *Kr-h1* in *P. stali*

dsRNA[1]	N	Death	Normal nymph	Precocious adult	Nymph with tiny wings[2]
Kr-h1	38	13	0	12	13
Control	19	0	19	0	0

[1]Double-stranded RNA preparations targeting *Kr-h1* gene (Kr-h1) and β-lactamase gene (Control) were injected into 4th instar nymphs, and their phenotypes were inspected upon 5th instar molt.
[2]These insects were not used for further analyses.

Table S3. Differentially expressed insect genes upregulated in the nymphal symbiotic organ of *P. stali.*

Rank [1]	Contig ID	Description	TPM [2]				
			4th instar	5th instar [3]	Adult	*E93* RNAi	*Kr-h1* RNAi
1	TRINITY_DN871_c0_g1_i1	Histone H2B	9728.9	9647.3	1110.7	5923.7	2544.3
2	TRINITY_DN4503_c0_g1_i1	Histone H3.3	1998.7	2401.8	189.8	1476.7	797.2
3	TRINITY_DN25610_c0_g2_i1	Histone H2A	1291.8	1440.4	149.1	735.3	411.5
4	TRINITY_DN4246_c1_g2_i2	No hit	2.2	886.0	1.7	1229.3	1708.7
5	TRINITY_DN7703_c0_g1_i2	Uncharacterized protein LOC106681859	635.1	254.6	22.5	622.6	186.6
6	TRINITY_DN6101_c0_g1_i27	Farnesol dehydrogenase-like	75.3	132.7	6.1	57.9	10.8
7	TRINITY_DN4640_c0_g1_i2	No hit	72.3	85.2	4.8	74.3	17.0
8	TRINITY_DN11919_c0_g3_i1	No hit	228.2	82.6	4.8	137.8	21.4
9	TRINITY_DN983_c0_g1_i1	Uncharacterized protein LOC106683306 isoform X1	5.4	75.1	0.2	17.0	0.5
10	TRINITY_DN11659_c0_g1_i2	Uncharacterized protein LOC106685697 isoform X2	15.5	49.8	0.1	46.6	0.3
11	TRINITY_DN1733_c1_g3_i1	Chitooligosaccharidolytic beta-N-acetylglucosaminidase	1.8	37.0	1.2	43.8	0.3
12	TRINITY_DN53902_c0_g1_i1	Uncharacterized protein LOC106684062	11.9	33.6	0.1	35.0	1.9
13	TRINITY_DN344_c0_g1_i2	G1/S-specific cyclin-D3-like, partial	14.3	28.0	2.9	24.6	6.4
14	TRINITY_DN2414_c1_g1_i7	Sodium-independent sulfate anion transporter isoform X2	30.8	23.0	0.1	32.9	0.1
15	TRINITY_DN42806_c0_g1_i1	Uncharacterized protein LOC106681789	91.4	16.1	0.6	49.1	8.4
16	TRINITY_DN1025_c0_g3_i1	Tubulin beta-1 chain isoform X1	15.4	14.7	0.7	9.4	2.2
17	TRINITY_DN1613_c0_g2_i2	No hit	4.2	12.2	0.4	6.4	0.4
18	TRINITY_DN2645_c0_g4_i1	No hit	2.9	10.6	0.1	3.6	0.4
19	TRINITY_DN27973_c0_g1_i8	Alpha-N-acetylgalactosaminidase	38.3	10.4	0.2	3.1	1.3
20	TRINITY_DN3439_c0_g1_i3	Angiomotin-like 2a isoform X4	4.7	10.3	0.5	7.6	1.0
21	TRINITY_DN3378_c0_g1_i1	Dynein light chain 1, cytoplasmic	4.7	9.9	0.8	8.2	1.5
22	TRINITY_DN12680_c0_g1_i2	Trafficking protein particle complex subunit 2	0.0	8.9	0.7	6.9	5.7
23	TRINITY_DN56_c0_g1_i29	No hit	2.1	7.2	0.0	3.1	3.6
24	TRINITY_DN4282_c0_g1_i12	No hit	7.0	7.1	0.0	15.6	0.1
25	TRINITY_DN221_c0_g1_i2	Exportin-1	2.7	6.9	0.0	7.7	6.0
26	TRINITY_DN4282_c0_g1_i13	No hit	4.2	6.7	0.2	9.5	0.2
27	TRINITY_DN13207_c0_g1_i1	Cyclin-dependent kinase 1	3.9	6.3	0.3	5.8	0.7
28	TRINITY_DN1431_c0_g2_i17	Tropomodulin-1 isoform X6	5.6	5.8	0.0	3.5	1.7
29	TRINITY_DN7305_c0_g1_i2	No hit	0.8	5.7	0.0	5.6	0.0
30	TRINITY_DN34895_c0_g1_i1	Serine/threonine-protein kinase polo	2.8	5.4	0.1	3.9	0.7
31	TRINITY_DN1858_c1_g1_i1	G2/mitotic-specific cyclin-B3	3.5	5.1	0.1	3.7	0.6
32	TRINITY_DN1521_c0_g1_i8	CGMP-dependent protein kinase, isozyme 2 forms cD5/T2 isoform X7	4.2	4.7	0.0	6.2	0.0
33	TRINITY_DN42411_c0_g1_i1	Maternal embryonic leucine zipper kinase-like isoform X1	2.6	4.6	0.3	4.1	0.8
34	TRINITY_DN50347_c0_g1_i1	No hit	1.3	4.3	0.0	2.4	0.0
35	TRINITY_DN179_c0_g1_i22	Hormone receptor 4 isoform X5	1.6	4.0	0.0	2.1	0.0
36	TRINITY_DN54297_c0_g1_i1	Pachytene checkpoint protein 2 homolog isoform X1	2.7	3.9	0.1	2.7	0.6
37	TRINITY_DN20373_c0_g1_i1	Ecdysone-induced protein 78C	0.7	3.8	0.0	2.2	0.3
38	TRINITY_DN31648_c0_g1_i1	No hit	8.9	3.7	0.0	1.9	0.0
39	TRINITY_DN1219_c0_g1_i18	Probable phosphoserine aminotransferase isoform X1	0.6	3.7	0.1	0.6	0.1
40	TRINITY_DN4583_c0_g1_i6	Rho-related BTB domain-containing protein 1	1.1	3.5	0.0	2.6	0.1
41	TRINITY_DN42593_c0_g1_i1	Venom carboxylesterase-6-like	1.7	3.4	0.1	3.1	0.4
42	TRINITY_DN3411_c0_g1_i1	No hit	2.2	3.2	0.0	1.8	0.0
43	TRINITY_DN11962_c0_g1_i4	Collagen alpha-5(IV) chain-like isoform X1	6.4	3.1	0.0	2.8	1.5
44	TRINITY_DN179_c0_g1_i20	Hormone receptor 4 isoform X5	0.9	2.9	0.0	1.7	0.0
45	TRINITY_DN38481_c0_g1_i1	N-acetyltransferase ESCO2	1.7	2.6	0.1	1.9	0.3
46	TRINITY_DN107_c0_g2_i1	No hit	1.4	2.5	0.0	1.0	0.0

[1] In the order of average expression levels in 5th instar nymphs.

[2] Average expression levels of four individuals.

[3] Significantly higher expression in 5th instar nymphs than in adults (FDR q < 0.01).

Table S4. Differentially expressed insect genes upregulated in the adult symbiotic organ of *P. stali.*

Rank [1]	Contig ID	Description	TPM [2]				
			4th instar	5th instar	Adult [3]	*E93* RNAi	*Kr-h1* RNAi
1	TRINITY_DN2568_c0_g1_i1	No hit	2.2	1.7	6513.0	99.4	3276.6
2	TRINITY_DN441_c1_g2_i11	No hit	4.4	24.3	2224.2	343.8	2432.4
3	TRINITY_DN179_c1_g1_i1	Uncharacterized protein	77.9	115.1	1010.8	196.3	882.4
4	TRINITY_DN2125_c0_g1_i1	Cathepsin L1-like	0.2	0.2	773.0	6.7	227.0
5	TRINITY_DN14573_c0_g1_i1	Cathepsin B-like	0.3	0.1	657.4	4.3	377.3
6	TRINITY_DN44596_c0_g1_i1	Probable salivary secreted peptide	0.7	2.3	459.8	1.5	52.2
7	TRINITY_DN223_c0_g3_i2	Endochitinase	260.5	28.7	416.5	184.1	284.7
8	TRINITY_DN1345_c0_g1_i6	Uncharacterized protein	0.2	2.9	304.8	74.6	148.9
9	TRINITY_DN7901_c0_g1_i1	No hit	0.2	1.2	262.9	131.4	271.2
10	TRINITY_DN5589_c0_g1_i4	Glutamine synthetase isoform X1	286.2	25.1	261.0	50.8	142.8
11	TRINITY_DN32999_c0_g1_i1	Uncharacterized protein	0.3	4.9	250.4	75.5	194.8
12	TRINITY_DN5750_c0_g1_i1	No hit	0.7	3.8	227.7	29.1	501.9
13	TRINITY_DN8710_c0_g2_i2	Laccase-4	0.4	0.3	223.0	3.7	88.8
14	TRINITY_DN14573_c0_g2_i1	Uncharacterized protein	0.5	0.6	206.6	10.8	155.3
15	TRINITY_DN7917_c0_g1_i2	No hit	7.3	10.3	171.3	28.1	47.4
16	TRINITY_DN36827_c0_g1_i1	No hit	1.0	4.3	164.9	54.1	231.8
17	TRINITY_DN50111_c0_g1_i1	Probable salivary secreted peptide	0.3	0.9	162.8	2.8	184.9
18	TRINITY_DN38775_c0_g1_i1	Putative serine protease	0.4	0.6	161.2	8.4	57.9
19	TRINITY_DN4566_c0_g1_i1	Uncharacterized protein	3.1	5.4	151.7	33.6	350.6
20	TRINITY_DN3330_c0_g1_i2	Trypsin-1-like	0.2	0.1	142.2	5.2	58.0
21	TRINITY_DN15747_c0_g1_i1	Aspartic proteinase A3-like	0.1	0.1	133.3	1.1	39.3
22	TRINITY_DN2734_c0_g1_i1	No hit	0.1	0.1	130.6	6.7	143.8
23	TRINITY_DN42448_c0_g1_i1	Venom carboxylesterase-6-like	0.1	0.1	125.7	2.0	68.4
24	TRINITY_DN46418_c0_g1_i1	Probable salivary secreted peptide	0.5	1.4	110.3	15.7	67.8
25	TRINITY_DN3347_c0_g1_i2	Uncharacterized protein	0.0	1.8	108.6	12.3	198.0
26	TRINITY_DN1554_c1_g1_i1	Cathepsin L1	10.2	7.3	103.2	13.6	42.3
27	TRINITY_DN55185_c0_g1_i1	Uncharacterized protein	0.6	1.1	92.8	2.3	40.8
28	TRINITY_DN42841_c0_g1_i1	Cathepsin L1-like	0.1	0.0	90.3	0.0	24.5
29	TRINITY_DN25545_c0_g1_i1	Uncharacterized protein	0.7	2.4	77.4	7.8	57.1
30	TRINITY_DN29861_c0_g1_i1	Uncharacterized protein	0.4	4.4	66.1	8.9	69.9
31	TRINITY_DN15137_c0_g1_i1	Uncharacterized protein	0.1	1.9	65.3	7.2	31.4
32	TRINITY_DN10486_c0_g1_i1	Uncharacterized protein	11.8	1.8	54.4	12.1	30.7
33	TRINITY_DN2329_c0_g1_i6	Procardosin-A	0.1	2.0	52.0	21.9	22.6
34	TRINITY_DN223_c0_g1_i6	Endochitinase	157.3	0.0	46.2	69.8	47.0
35	TRINITY_DN2122_c0_g1_i1	2-acylglycerol O-acyltransferase 1-like	0.8	3.0	39.7	8.9	34.3
36	TRINITY_DN287_c0_g1_i1	Alpha-glucosidase-like	0.2	0.4	36.6	0.7	14.7
37	TRINITY_DN2908_c3_g4_i1	Probable cytochrome P450	0.5	2.4	36.6	14.1	22.3
38	TRINITY_DN3347_c0_g1_i1	Uncharacterized protein	0.5	0.5	35.0	7.8	52.4
39	TRINITY_DN3695_c0_g1_i1	No hit	0.0	0.1	33.0	0.4	25.6
40	TRINITY_DN4459_c0_g1_i1	Uncharacterized protein	0.3	0.2	30.6	2.5	11.0
41	TRINITY_DN7823_c0_g1_i1	No hit	0.0	0.3	24.8	1.5	10.6
42	TRINITY_DN49824_c0_g1_i1	Legumain-like	0.0	0.0	24.1	0.1	15.9
43	TRINITY_DN223_c0_g1_i5	Endochitinase	37.8	0.0	24.1	18.4	18.4
44	TRINITY_DN5257_c0_g2_i1	Uncharacterized protein	0.0	0.0	24.0	0.1	5.4
45	TRINITY_DN13228_c0_g1_i1	No hit	0.2	0.3	22.0	0.9	7.8
46	TRINITY_DN362_c0_g1_i1	Uncharacterized protein	6.3	0.4	21.9	7.2	15.9
47	TRINITY_DN38752_c0_g1_i1	Uncharacterized protein	26.7	0.2	21.7	9.7	15.2
48	TRINITY_DN5203_c0_g1_i1	No hit	0.0	0.1	21.0	3.4	14.3
49	TRINITY_DN12648_c1_g2_i1	No hit	14.3	0.0	19.9	50.8	36.8
50	TRINITY_DN5968_c0_g1_i1	Uncharacterized protein	0.5	0.2	18.5	0.1	18.6
51	TRINITY_DN5727_c0_g1_i3	No hit	16.0	0.0	17.5	8.6	7.2
52	TRINITY_DN5736_c0_g1_i2	Alpha-tocopherol transfer protein-like	0.0	0.2	15.9	1.3	12.3
53	TRINITY_DN10039_c0_g1_i1	Uncharacterized protein	0.1	0.2	15.8	0.9	11.9
54	TRINITY_DN26236_c0_g1_i2	Uncharacterized protein	0.0	0.1	15.5	0.6	6.6
55	TRINITY_DN24582_c0_g1_i1	No hit	0.0	0.5	15.4	4.1	10.2
56	TRINITY_DN14751_c0_g1_i1	No hit	0.0	0.0	14.6	0.0	5.6
57	TRINITY_DN35973_c0_g1_i1	No hit	0.0	0.1	14.5	1.8	26.8
58	TRINITY_DN4696_c0_g1_i1	Small nuclear ribonucleoprotein	26.1	0.0	14.3	19.0	14.7
59	TRINITY_DN47660_c0_g1_i1	Uncharacterized protein	0.0	0.2	13.8	0.1	6.9
60	TRINITY_DN9852_c0_g3_i3	Cardioactive peptide	0.0	0.9	13.2	1.0	20.6
61	TRINITY_DN5057_c0_g1_i1	No hit	4.0	0.0	13.0	4.9	7.8
62	TRINITY_DN50805_c0_g1_i1	Protein takeout-like	0.0	0.0	13.0	2.6	9.2
63	TRINITY_DN13149_c0_g1_i1	Phospholipase A1-like isoform X1	0.0	0.2	12.6	5.6	6.3
64	TRINITY_DN55140_c0_g1_i1	No hit	0.2	0.2	11.9	1.5	19.9
65	TRINITY_DN1496_c0_g1_i5	No hit	0.7	0.6	11.6	10.0	5.0
66	TRINITY_DN927_c0_g1_i1	Protein MEMO1	12.5	0.0	10.8	8.2	6.0
67	TRINITY_DN3330_c0_g1_i3	Trypsin-1-like	0.1	0.0	10.5	0.3	4.5

68	TRINITY_DN1599_c0_g1_i6	Acylamino-acid-releasing enzyme-like isoform X1	0.0	0.0	10.4	0.0	2.3
69	TRINITY_DN8946_c0_g1_i1	No hit	0.3	0.4	9.3	0.2	2.0
70	TRINITY_DN12680_c0_g1_i1	Trafficking protein particle complex subunit 2	13.8	0.0	9.2	7.6	4.8
71	TRINITY_DN1654_c0_g1_i1	Endochitinase	23.5	0.0	8.0	33.4	10.1
72	TRINITY_DN2106_c0_g1_i8	Aspartic proteinase A3 isoform X2	0.0	0.1	8.0	0.3	4.5
73	TRINITY_DN5460_c0_g1_i3	ADP-ribose pyrophosphatase, mitochondrial isoform X1	7.4	0.0	7.6	7.0	6.1
74	TRINITY_DN12648_c1_g1_i9	No hit	37.7	0.0	7.1	23.3	5.3
75	TRINITY_DN15934_c0_g1_i2	Alanine aminotransferase 1-like	0.4	0.1	7.1	1.0	2.4
76	TRINITY_DN11243_c1_g2_i1	UDP-glucuronosyltransferase	1.5	0.7	7.0	3.1	5.9
77	TRINITY_DN3332_c0_g1_i2	Carboxypeptidase E	2.0	0.1	6.5	3.8	3.7
78	TRINITY_DN42414_c0_g1_i1	Maltase A1-like	0.0	0.0	6.4	0.7	6.5
79	TRINITY_DN54035_c0_g1_i1	Cathepsin L1-like	0.0	0.1	6.4	0.0	1.3
80	TRINITY_DN8634_c0_g2_i2	Sodium/potassium/calcium exchanger 4-like isoform X2	1.1	0.4	5.9	2.1	4.0
81	TRINITY_DN2088_c0_g1_i12	Copper-transporting ATPase 1 isoform X2	0.1	0.1	5.8	0.2	5.1
82	TRINITY_DN15322_c0_g1_i1	Cathepsin L1 isoform X1	0.0	0.0	5.7	0.0	4.1
83	TRINITY_DN2160_c0_g1_i9	CAAX prenyl protease 1 homolog	16.5	0.0	5.7	10.2	4.8
84	TRINITY_DN32400_c0_g1_i1	Cathepsin L1-like	0.0	0.1	5.6	0.0	0.2
85	TRINITY_DN19240_c0_g1_i3	Uncharacterized protein	0.0	0.0	5.4	0.3	2.3
86	TRINITY_DN468_c1_g1_i14	Alanine aminotransferase 1-like	0.4	0.2	5.1	0.5	2.2
87	TRINITY_DN7379_c0_g1_i16	Serine hydroxymethyltransferase isoform X1	2.3	0.0	4.9	1.4	2.3
88	TRINITY_DN362_c0_g1_i2	Uncharacterized protein	1.1	0.1	4.9	1.0	4.1
89	TRINITY_DN4215_c0_g1_i1	Uncharacterized protein	0.0	0.0	4.9	0.1	1.4
90	TRINITY_DN46250_c0_g1_i1	Uncharacterized protein	0.0	0.0	4.8	0.0	4.7
91	TRINITY_DN39051_c0_g1_i1	Venom serine carboxypeptidase	0.1	0.1	4.7	0.4	2.1
92	TRINITY_DN2208_c0_g1_i7	Uncharacterized protein	0.0	0.0	4.7	0.4	1.0
93	TRINITY_DN5269_c0_g1_i12	Uncharacterized protein	2.1	0.0	4.7	3.2	3.8
94	TRINITY_DN3854_c0_g1_i6	No hit	2.1	0.0	4.5	1.9	2.8
95	TRINITY_DN1496_c0_g1_i25	Retinal dehydrogenase 1-like	0.6	0.0	4.3	5.2	0.3
96	TRINITY_DN25496_c0_g1_i1	Spondin-1	0.2	0.2	3.9	0.9	4.4
97	TRINITY_DN27227_c0_g1_i1	Monocarboxylate transporter 14 isoform X2	0.0	0.1	3.8	0.5	4.7
98	TRINITY_DN36239_c0_g1_i1	No hit	0.2	0.0	3.4	0.1	2.4
99	TRINITY_DN8614_c0_g1_i17	Monocarboxylate transporter 13	0.1	0.0	3.3	0.4	0.4
100	TRINITY_DN49863_c0_g1_i1	Elongation of very long chain fatty acids protein	0.1	0.1	3.1	1.0	1.7

[1] In the order of average expression levels in adults.

[2] Average expression levels of four individuals.

[3] Significantly higher expression in adults than 5th instar nymphs (FDR q < 0.01).

Table S5. Differentially expressed insect genes upregulated in the adult crypts of *P. stali* .

Rank [1]	Contig ID	Description	TPM [2]	
			Crypts [3]	Main tract
1	TRINITY_DN1704_c0_g2_i2	No hit	72224.7	17509.7
2	TRINITY_DN6587_c0_g1_i1	No hit	25625.8	5597.1
3	TRINITY_DN4246_c1_g1_i5	No hit	24373.1	6768.5
4	TRINITY_DN8018_c3_g1_i1	No hit	21892.5	5591.9
5	TRINITY_DN1813_c0_g1_i10	No hit	15105.1	4574.4
6	TRINITY_DN846_c0_g1_i2	No hit	14319.4	3851.4
7	TRINITY_DN3178_c0_g1_i2	No hit	12256.2	3077.4
8	TRINITY_DN6178_c0_g3_i18	Gamma-glutamyl hydrolase-like	10835.4	2305.3
9	TRINITY_DN1545_c1_g1_i28	No hit	9970.7	2719.1
10	TRINITY_DN2941_c0_g2_i1	No hit	9400.4	3129.9
11	TRINITY_DN1545_c1_g1_i9	No hit	8531.4	2276.4
12	TRINITY_DN1509_c1_g1_i6	No hit	8195.7	2487.6
13	TRINITY_DN2374_c1_g3_i1	No hit	8156.7	2604.6
14	TRINITY_DN129_c0_g1_i1	No hit	7186.6	2053.9
15	TRINITY_DN749_c0_g1_i1	No hit	5902.3	1535.7
16	TRINITY_DN6250_c0_g1_i27	Uncharacterized protein LOC106692977 isoform X2	5194.6	1571.4
17	TRINITY_DN5378_c0_g1_i1	No hit	5076.8	1413.1
18	TRINITY_DN1545_c1_g1_i1	No hit	4927.4	1052.7
19	TRINITY_DN4246_c1_g2_i2	No hit	3874.1	1102.7
20	TRINITY_DN5436_c0_g1_i1	No hit	3721.9	1219.9
21	TRINITY_DN297_c0_g1_i2	No hit	3487.9	1074.8
22	TRINITY_DN362_c1_g4_i1	No hit	3244.5	940.8
23	TRINITY_DN2571_c0_g1_i12	No hit	3226.7	701.2
24	TRINITY_DN4416_c1_g1_i5	No hit	3087.4	755.1
25	TRINITY_DN3606_c0_g1_i1	No hit	2610.4	707.0
26	TRINITY_DN2172_c0_g1_i2	No hit	2413.5	771.3
27	TRINITY_DN1686_c2_g3_i5	No hit	2204.6	661.2
28	TRINITY_DN433_c0_g1_i4	Activating transcription factor of chaperone	2176.4	534.8
29	TRINITY_DN4384_c1_g2_i2	No hit	2149.6	370.8
30	TRINITY_DN4448_c0_g1_i1	No hit	2130.3	658.4
31	TRINITY_DN23_c0_g2_i3	Transketolase	2102.8	440.5
32	TRINITY_DN5092_c0_g1_i1	No hit	1690.2	474.9
33	TRINITY_DN1577_c0_g1_i3	Aldehyde dehydrogenase, mitochondrial-like	1587.5	522.7
34	TRINITY_DN2253_c0_g1_i10	No hit	1568.3	380.5
35	TRINITY_DN385_c0_g1_i3	No hit	1464.7	329.2
36	TRINITY_DN396_c0_g2_i4	No hit	1430.6	63.9
37	TRINITY_DN362_c1_g3_i1	No hit	1104.9	359.4
38	TRINITY_DN3692_c0_g1_i12	Lysosomal aspartic protease-like	1089.9	297.7
39	TRINITY_DN147_c6_g1_i2	No hit	1084.2	186.3
40	TRINITY_DN396_c0_g3_i1	No hit	1035.1	36.8
41	TRINITY_DN56954_c0_g1_i1	No hit	1001.4	132.9
42	TRINITY_DN49_c0_g1_i4	Facilitated trehalose transporter Tret1-like	984.3	233.7
43	TRINITY_DN3692_c0_g1_i35	Lysosomal aspartic protease-like	966.4	292.4
44	TRINITY_DN1103_c0_g1_i1	No hit	959.3	212.6
45	TRINITY_DN1496_c0_g1_i22	Retinal dehydrogenase 1-like	941.2	242.7
46	TRINITY_DN2586_c0_g1_i3	No hit	908.3	234.7
47	TRINITY_DN657_c0_g1_i11	Ammonium transporter Rh type C-like 2	792.3	200.8
48	TRINITY_DN9473_c0_g1_i1	No hit	760.4	173.5
49	TRINITY_DN3105_c0_g1_i7	No hit	675.5	192.0
50	TRINITY_DN706_c0_g1_i12	No hit	669.0	145.6
51	TRINITY_DN700_c0_g2_i5	No hit	650.9	188.8
52	TRINITY_DN35_c0_g1_i19	No hit	633.6	155.7
53	TRINITY_DN4390_c0_g1_i10	No hit	628.2	197.3
54	TRINITY_DN6178_c0_g3_i15	No hit	590.2	114.0
55	TRINITY_DN1199_c1_g1_i1	Cathepsin L1-like isoform X1	580.2	113.7
56	TRINITY_DN396_c0_g1_i2	No hit	568.1	88.2
57	TRINITY_DN530_c0_g1_i1	No hit	564.8	132.9
58	TRINITY_DN1220_c0_g1_i24	Facilitated glucose transporter member 5-like isoform X1	559.5	133.0
59	TRINITY_DN951_c0_g1_i5	Cathepsin L1-like	540.2	136.0
60	TRINITY_DN2192_c0_g1_i1	Ribonuclease T2 isoform X1	533.3	130.3
61	TRINITY_DN6178_c0_g3_i19	Gamma-glutamyl hydrolase-like	523.3	142.8
62	TRINITY_DN2679_c0_g1_i24	Ribokinase-like isoform X1	518.3	120.6
63	TRINITY_DN6178_c0_g3_i31	No hit	518.1	132.2
64	TRINITY_DN2738_c0_g1_i98	Phosphoenolpyruvate carboxykinase [GTP]-like isoform X1	512.2	144.6
65	TRINITY_DN3287_c0_g1_i6	Facilitated trehalose transporter Tret1-like isoform X1	511.8	125.1
66	TRINITY_DN6857_c0_g1_i1	Aminopeptidase N isoform X1	497.9	151.8
67	TRINITY_DN964_c0_g2_i3	No hit	496.2	163.1
68	TRINITY_DN396_c0_g2_i9	No hit	482.4	2.0
69	TRINITY_DN3093_c0_g1_i13	No hit	459.3	113.6

70	TRINITY_DN2571_c0_g1_i3	No hit	443.2	110.9
71	TRINITY_DN12088_c0_g1_i2	No hit	431.2	98.2
72	TRINITY_DN2346_c0_g1_i36	Solute carrier family 26 member 10-like isoform X2	403.9	68.2
73	TRINITY_DN4065_c0_g1_i1	No hit	402.2	100.5
74	TRINITY_DN45_c0_g1_i18	Monocarboxylate transporter 10	398.9	86.9
75	TRINITY_DN2490_c1_g1_i9	No hit	397.8	75.0
76	TRINITY_DN3466_c1_g1_i1	Serine/threonine-protein phosphatase 6 regulatory ankyrin repeat subunit A	395.5	88.4
77	TRINITY_DN1854_c0_g1_i1	No hit	393.6	104.3
78	TRINITY_DN3354_c0_g1_i8	Facilitated trehalose transporter Tret1-like	392.4	98.3
79	TRINITY_DN530_c0_g1_i7	No hit	389.4	89.4
80	TRINITY_DN3063_c0_g1_i15	Cathepsin B-like	381.6	79.2
81	TRINITY_DN4535_c0_g1_i7	Pyridoxine-5'-phosphate oxidase-like	375.7	105.9
82	TRINITY_DN1590_c8_g1_i1	No hit	367.4	112.3
83	TRINITY_DN108_c0_g1_i18	Probable acid phosphatase DDB G0284755	367.2	84.9
84	TRINITY_DN4246_c1_g2_i3	No hit	357.5	91.7
85	TRINITY_DN167_c1_g5_i1	Purine nucleoside phosphorylase-like	343.9	77.3
86	TRINITY_DN530_c0_g1_i5	No hit	337.8	95.0
87	TRINITY_DN15911_c1_g1_i1	No hit	336.0	94.5
88	TRINITY_DN1761_c0_g1_i9	No hit	334.8	90.6
89	TRINITY_DN3217_c0_g1_i2	Kynurenine oxoglutarate transaminase 3 isoform X1	322.7	80.9
90	TRINITY_DN1065_c0_g1_i11	6-phosphofructo-2-kinase/fructose-2,6-bisphosphatase	319.7	84.1
91	TRINITY_DN2674_c3_g1_i1	No hit	315.8	85.1
92	TRINITY_DN731_c0_g4_i1	Acid phosphatase type 7-like	308.8	83.1
93	TRINITY_DN147_c2_g1_i23	Proton-coupled amino acid transporter-like protein CG1139	308.5	19.6
94	TRINITY_DN13003_c0_g1_i1	No hit	300.7	36.3
95	TRINITY_DN1275_c2_g1_i3	No hit	296.5	72.7
96	TRINITY_DN2326_c0_g1_i1	D-3-phosphoglycerate dehydrogenase	284.1	64.1
97	TRINITY_DN468_c1_g1_i8	Alanine aminotransferase 1-like	273.3	67.1
98	TRINITY_DN8728_c0_g1_i3	Monocarboxylate transporter 13 isoform X2	270.8	71.2
99	TRINITY_DN54_c1_g1_i4	Sodium-independent sulfate anion transporter-like	267.9	46.8
100	TRINITY_DN1445_c0_g2_i1	Phosphotriesterase-related protein	253.5	54.9
101	TRINITY_DN764_c0_g1_i7	Glutathione hydrolase 1 proenzyme isoform X1	250.0	55.2
102	TRINITY_DN6269_c0_g1_i2	No hit	241.9	43.9
103	TRINITY_DN4685_c1_g2_i1	Cystathionine beta-synthase	232.8	63.1
104	TRINITY_DN2829_c0_g1_i3	Branched-chain-amino-acid aminotransferase	231.9	57.1
105	TRINITY_DN25144_c0_g1_i1	No hit	230.7	60.8
106	TRINITY_DN1051_c2_g1_i1	No hit	229.0	69.3
107	TRINITY_DN52_c4_g1_i3	No hit	209.0	35.3
108	TRINITY_DN810_c0_g2_i4	No hit	206.7	57.5
109	TRINITY_DN3186_c0_g1_i5	No hit	203.1	48.4
110	TRINITY_DN4409_c0_g2_i3	No hit	201.9	44.4
111	TRINITY_DN2175_c0_g1_i5	Solute carrier family 25 member 35	200.8	54.7
112	TRINITY_DN1704_c0_g1_i2	No hit	200.5	62.9
113	TRINITY_DN204_c2_g1_i2	Aquaporin AQPcic isoform X2	200.5	51.0
114	TRINITY_DN756_c1_g1_i4	No hit	200.5	37.6
115	TRINITY_DN2346_c0_g1_i48	Solute carrier family 26 member 10-like isoform X3	195.3	33.0
116	TRINITY_DN1341_c0_g3_i3	No hit	194.1	34.5
117	TRINITY_DN2738_c0_g1_i222	Phosphoenolpyruvate carboxykinase [GTP]-like isoform X2	193.2	43.7
118	TRINITY_DN6250_c0_g1_i15	Uncharacterized protein LOC106692977 isoform X2	191.9	52.2
119	TRINITY_DN507_c0_g4_i4	Probable low-specificity L-threonine aldolase 1	191.8	37.8
120	TRINITY_DN2791_c4_g1_i1	No hit	187.2	55.6
121	TRINITY_DN2571_c0_g1_i7	No hit	186.5	35.6
122	TRINITY_DN578_c0_g1_i4	Putative inorganic phosphate cotransporter isoform X1	184.4	55.2
123	TRINITY_DN1763_c0_g1_i1	Facilitated trehalose transporter Tret1-like	180.0	45.4
124	TRINITY_DN6210_c0_g3_i2	No hit	176.8	5.6
125	TRINITY_DN17011_c0_g1_i1	No hit	172.7	50.6
126	TRINITY_DN716_c0_g3_i7	Succinate-semialdehyde dehydrogenase, mitochondrial isoform X1	171.8	45.7
127	TRINITY_DN378_c0_g1_i3	No hit	171.7	45.3
128	TRINITY_DN4469_c0_g1_i1	No hit	170.4	49.8
129	TRINITY_DN1982_c0_g1_i5	Esterase E4-like	169.8	35.9
130	TRINITY_DN2034_c0_g1_i7	Phosphoserine phosphatase isoform X1	161.6	40.7
131	TRINITY_DN355_c1_g2_i1	No hit	157.1	33.0
132	TRINITY_DN1509_c1_g1_i1	No hit	156.3	48.2
133	TRINITY_DN4065_c0_g1_i3	No hit	154.4	40.6
134	TRINITY_DN530_c0_g1_i11	No hit	152.1	26.6
135	TRINITY_DN931_c0_g2_i1	Uncharacterized protein LOC112211292	152.0	40.7
136	TRINITY_DN2386_c0_g1_i1	No hit	149.1	43.7
137	TRINITY_DN197_c0_g1_i1	No hit	146.2	1.7
138	TRINITY_DN45_c0_g1_i2	Monocarboxylate transporter 10	143.9	42.7
139	TRINITY_DN810_c0_g1_i20	No hit	143.5	30.7
140	TRINITY_DN1715_c0_g1_i5	No hit	143.0	45.5
141	TRINITY_DN322_c0_g1_i39	Uncharacterized protein LOC106688030	142.3	42.5

142	TRINITY_DN810_c0_g1_i9	No hit	140.8	29.0
143	TRINITY_DN3186_c0_g1_i2	No hit	140.4	42.3
144	TRINITY_DN1448_c1_g4_i4	Cathepsin B-like isoform X1	136.5	23.4
145	TRINITY_DN322_c0_g1_i53	Uncharacterized protein LOC106688030	132.4	10.5
146	TRINITY_DN2556_c0_g1_i1	Reticulon-1-like isoform X2	127.7	28.1
147	TRINITY_DN16333_c1_g1_i1	No hit	127.0	33.4
148	TRINITY_DN44_c0_g1_i6	No hit	126.7	32.0
149	TRINITY_DN329_c0_g1_i3	Sideroflexin-3	123.6	27.4
150	TRINITY_DN1885_c0_g1_i17	Fructose-bisphosphate aldolase	121.9	31.6
151	TRINITY_DN2783_c0_g1_i112	Probable cation-transporting ATPase 13A3	121.0	32.2
152	TRINITY_DN3217_c0_g1_i10	Kynurenine--oxoglutarate transaminase 3 isoform X1	119.7	36.5
153	TRINITY_DN2571_c0_g1_i9	No hit	118.5	26.3
154	TRINITY_DN3466_c1_g1_i2	Serine/threonine-protein phosphatase 6 regulatory ankyrin repeat subunit A	117.8	27.9
155	TRINITY_DN5915_c0_g3_i1	Aquaporin AQPAe.a-like isoform X4	116.5	32.9
156	TRINITY_DN468_c1_g1_i2	Alanine aminotransferase 1-like	110.5	23.3
157	TRINITY_DN669_c0_g3_i2	Uncharacterized protein LOC106681193 isoform X7	106.3	25.6
158	TRINITY_DN706_c0_g1_i7	Esterase FE4-like	104.9	17.5
159	TRINITY_DN2763_c0_g1_i16	Protein SERAC1 isoform X4	103.2	31.0
160	TRINITY_DN8651_c0_g1_i3	Protein FAM107B	100.9	31.5
161	TRINITY_DN1733_c1_g3_i2	No hit	99.5	14.1
162	TRINITY_DN2495_c1_g2_i1	EF-hand domain-containing protein D2 homolog	98.5	27.9
163	TRINITY_DN1448_c1_g4_i2	Cathepsin B-like isoform X2	92.0	20.1
164	TRINITY_DN3443_c0_g1_i2	Probable cation-transporting ATPase 13A3	91.0	24.3
165	TRINITY_DN6101_c2_g1_i1	No hit	90.7	17.9
166	TRINITY_DN1635_c3_g2_i9	Uncharacterized protein LOC106681799 isoform X2	90.6	17.6
167	TRINITY_DN3462_c0_g1_i1	No hit	90.3	27.5
168	TRINITY_DN2453_c0_g1_i1	No hit	90.2	26.5
169	TRINITY_DN1502_c0_g1_i5	No hit	89.0	18.9
170	TRINITY_DN1051_c1_g2_i3	No hit	87.9	26.5
171	TRINITY_DN1554_c0_g3_i1	No hit	87.9	19.7
172	TRINITY_DN1913_c0_g1_i6	No hit	87.8	23.1
173	TRINITY_DN530_c0_g1_i8	No hit	87.6	20.6
174	TRINITY_DN2679_c0_g1_i33	Ribokinase-like isoform X1	85.4	17.3
175	TRINITY_DN3093_c0_g1_i14	Uncharacterized protein LOC106692980	84.4	24.5
176	TRINITY_DN314_c0_g1_i31	Cystathionine gamma-lyase-like	83.8	20.5
177	TRINITY_DN10988_c0_g1_i10	Serine/arginine repetitive matrix protein 4	83.5	22.1
178	TRINITY_DN147_c2_g3_i1	Proton-coupled amino acid transporter-like protein CG1139	81.2	22.3
179	TRINITY_DN3093_c0_g1_i2	Uncharacterized protein LOC106692980	79.5	21.1
180	TRINITY_DN223_c0_g1_i10	Endochitinase At2g43620-like	77.9	21.6
181	TRINITY_DN1763_c0_g1_i3	Facilitated trehalose transporter Tret1-like	77.4	17.8
182	TRINITY_DN18322_c2_g2_i2	No hit	77.2	21.7
183	TRINITY_DN2330_c0_g1_i1	No hit	77.1	14.4
184	TRINITY_DN1065_c0_g1_i2	6-phosphofructo-2-kinase/fructose-2,6-bisphosphatase	76.0	17.9
185	TRINITY_DN1731_c0_g2_i4	Enolase-phosphatase E1	72.6	16.1
186	TRINITY_DN8728_c0_g2_i4	Uncharacterized protein LOC106678977	71.9	15.8
187	TRINITY_DN1831_c0_g1_i4	Tubulin beta chain-like isoform X1	70.5	15.2
188	TRINITY_DN8401_c0_g1_i5	Glutathione S-transferase	69.8	20.1
189	TRINITY_DN2962_c0_g2_i18	Uncharacterized protein LOC106684164	68.9	15.4
190	TRINITY_DN223_c0_g3_i2	Endochitinase At2g43620-like	68.5	17.5
191	TRINITY_DN3063_c0_g1_i5	Cathepsin B-like	68.2	21.9
192	TRINITY_DN3283_c0_g1_i2	Prostatic acid phosphatase-like	67.0	17.7
193	TRINITY_DN322_c0_g1_i84	Uncharacterized protein LOC106688030	66.0	17.6
194	TRINITY_DN30268_c0_g1_i1	Zinc finger protein Noc	65.4	10.1
195	TRINITY_DN2034_c1_g1_i16	No hit	65.3	18.9
196	TRINITY_DN1200_c0_g3_i1	No hit	65.1	10.9
197	TRINITY_DN2648_c0_g1_i2	No hit	65.0	19.8
198	TRINITY_DN2609_c1_g1_i5	No hit	65.0	19.1
199	TRINITY_DN706_c0_g1_i5	Esterase FE4-like	63.9	19.3
200	TRINITY_DN726_c0_g1_i13	Puratrophin-1 isoform X3	62.5	12.4
201	TRINITY_DN4501_c0_g1_i16	MAGUK p55 subfamily member 5 isoform X2	61.2	16.1
202	TRINITY_DN4025_c1_g1_i4	No hit	60.0	13.4
203	TRINITY_DN1839_c0_g1_i2	No hit	59.9	17.2
204	TRINITY_DN147_c2_g1_i24	Proton-coupled amino acid transporter-like protein CG1139	59.7	0.0
205	TRINITY_DN2763_c0_g1_i38	Protein SERAC1 isoform X3	59.5	12.4
206	TRINITY_DN213_c0_g1_i39	Solute carrier organic anion transporter family member 5A1	58.9	16.4
207	TRINITY_DN3692_c0_g1_i33	Lysosomal aspartic protease	56.6	12.2
208	TRINITY_DN7075_c0_g1_i1	No hit	56.5	1.9
209	TRINITY_DN4280_c0_g1_i12	DP-N-acetylglucosamine--peptide N-acetylglucosaminyltransferase 110 kDa subu	56.4	16.6
210	TRINITY_DN17067_c0_g1_i3	FGGY carbohydrate kinase domain-containing protein	55.8	16.7
211	TRINITY_DN700_c0_g2_i3	No hit	55.6	10.3
212	TRINITY_DN5414_c0_g1_i1	No hit	55.4	12.5
213	TRINITY_DN8_c0_g1_i41	Secretin receptor-like	54.7	10.5

214	TRINITY_DN3560_c0_g1_i4	Down syndrome cell adhesion molecule-like protein Dscam2 isoform X3	54.4	11.9
215	TRINITY_DN1509_c1_g1_i2	No hit	53.6	14.8
216	TRINITY_DN49_c0_g1_i8	Facilitated trehalose transporter Tret1-like	52.9	14.0
217	TRINITY_DN1444_c1_g1_i1	No hit	52.9	12.8
218	TRINITY_DN11752_c0_g1_i1	Alpha-tocopherol transfer protein-like isoform X2	51.9	15.4
219	TRINITY_DN2783_c0_g1_i81	No hit	50.9	11.5
220	TRINITY_DN8332_c0_g1_i9	No hit	49.9	13.0
221	TRINITY_DN2847_c1_g1_i11	Alpha,alpha-trehalose-phosphate synthase [UDP-forming] isoform X2	49.6	12.3
222	TRINITY_DN8614_c0_g1_i61	Monocarboxylate transporter 13 isoform X2	48.9	13.8
223	TRINITY_DN3093_c0_g1_i6	No hit	47.0	14.3
224	TRINITY_DN7799_c0_g1_i1	Cytochrome P450 4C1	46.8	12.8
225	TRINITY_DN14925_c0_g1_i1	Adenosylhomocysteinase	46.7	14.6
226	TRINITY_DN5378_c0_g1_i2	No hit	46.6	12.4
227	TRINITY_DN1460_c0_g1_i9	Cationic amino acid transporter 2 isoform X1	46.3	15.3
228	TRINITY_DN2374_c1_g1_i1	No hit	45.8	11.7
229	TRINITY_DN13651_c0_g1_i2	Insulin-like growth factor-binding protein complex acid labile subunit	45.8	14.4
230	TRINITY_DN3178_c0_g1_i1	No hit	45.6	13.0
231	TRINITY_DN2763_c0_g1_i26	Protein SERAC1 isoform X1	45.6	9.3
232	TRINITY_DN3186_c0_g1_i1	No hit	45.5	13.9
233	TRINITY_DN393_c0_g1_i59	Uncharacterized protein LOC106686172, partial	45.4	10.0
234	TRINITY_DN739_c1_g1_i7	No hit	44.1	4.2
235	TRINITY_DN12648_c1_g1_i12	No hit	44.0	4.6
236	TRINITY_DN706_c0_g1_i9	No hit	43.7	9.2
237	TRINITY_DN111_c1_g1_i6	Sedoheptulokinase-like	43.1	10.5
238	TRINITY_DN739_c1_g2_i5	Large neutral amino acids transporter small subunit 2	42.8	11.2
239	TRINITY_DN755_c0_g1_i8	No hit	42.7	0.8
240	TRINITY_DN106_c0_g1_i4	RNA-binding protein Musashi homolog 2 isoform X3	42.0	10.5
241	TRINITY_DN8_c0_g1_i51	Secretin receptor-like	41.6	7.5
242	TRINITY_DN884_c0_g2_i1	Regucalcin-like	41.0	7.0
243	TRINITY_DN2243_c0_g1_i16	Y+L amino acid transporter 2	39.9	9.2
244	TRINITY_DN223_c0_g3_i4	Endochitinase At2g43620-like	39.5	9.1
245	TRINITY_DN3945_c0_g1_i1	No hit	39.3	8.0
246	TRINITY_DN111_c1_g1_i4	Sedoheptulokinase-like	39.3	9.5
247	TRINITY_DN2015_c1_g1_i8	Bifunctional 3'-phosphoadenosine 5'-phosphosulfate synthase isoform X1	39.2	11.8
248	TRINITY_DN1593_c0_g1_i52	Down syndrome cell adhesion molecule-like protein Dscam2 isoform X3	39.1	8.1
249	TRINITY_DN25598_c0_g1_i3	Uncharacterized protein LOC106686567	38.5	10.2
250	TRINITY_DN4312_c0_g1_i15	D-2-hydroxyglutarate dehydrogenase, mitochondrial isoform X1	38.2	10.9
251	TRINITY_DN767_c0_g2_i8	No hit	38.2	9.1
252	TRINITY_DN2046_c1_g1_i1	Protein 5NUC-like	38.0	8.3
253	TRINITY_DN4436_c0_g1_i2	Arylsulfatase B-like	37.6	9.7
254	TRINITY_DN3603_c0_g1_i1	Equilibrative nucleoside transporter 1 isoform X1	37.6	7.1
255	TRINITY_DN15386_c0_g1_i1	No hit	37.6	10.3
256	TRINITY_DN6210_c0_g3_i1	No hit	36.6	0.4
257	TRINITY_DN26441_c0_g1_i6	Hexokinase-2-like isoform X1	36.4	8.9
258	TRINITY_DN3539_c0_g1_i13	Angiotensin-converting enzyme-like isoform X1	36.2	1.0
259	TRINITY_DN147_c1_g2_i1	No hit	36.1	10.2
260	TRINITY_DN876_c0_g1_i2	Facilitated trehalose transporter Tret1	35.4	8.1
261	TRINITY_DN3109_c0_g1_i3	No hit	35.4	8.3
262	TRINITY_DN297_c0_g1_i1	No hit	35.1	9.8
263	TRINITY_DN4519_c0_g1_i9	Neuropeptide Y receptor type 5	34.8	8.7
264	TRINITY_DN6423_c0_g1_i2	No hit	33.8	9.5
265	TRINITY_DN1686_c2_g3_i10	No hit	33.7	4.4
266	TRINITY_DN3093_c0_g1_i4	No hit	33.6	8.1
267	TRINITY_DN20683_c0_g1_i1	No hit	33.4	9.6
268	TRINITY_DN4965_c0_g2_i1	No hit	32.8	6.8
269	TRINITY_DN1509_c1_g1_i4	No hit	32.6	9.3
270	TRINITY_DN5952_c0_g1_i22	Sodium-dependent nutrient amino acid transporter 1-like isoform X3	32.4	7.3
271	TRINITY_DN2352_c0_g2_i7	No hit	31.7	6.2
272	TRINITY_DN716_c0_g3_i15	Succinate-semialdehyde dehydrogenase, mitochondrial isoform X1	31.7	8.0
273	TRINITY_DN1496_c0_g1_i19	Retinal dehydrogenase 1-like	31.5	1.7
274	TRINITY_DN11095_c0_g1_i2	No hit	31.3	3.0
275	TRINITY_DN5871_c0_g1_i1	No hit	31.1	9.0
276	TRINITY_DN1977_c0_g1_i6	No hit	30.8	6.1
277	TRINITY_DN4274_c0_g1_i1	No hit	30.5	7.8
278	TRINITY_DN1545_c1_g1_i15	No hit	30.3	7.3
279	TRINITY_DN810_c0_g1_i28	No hit	30.0	7.1
280	TRINITY_DN846_c0_g1_i7	No hit	29.9	9.4
281	TRINITY_DN23_c0_g2_i2	Transketolase	29.7	2.2
282	TRINITY_DN315_c1_g1_i9	SRSF protein kinase 3-like	29.4	7.0
283	TRINITY_DN1895_c0_g2_i8	No hit	29.0	7.0
284	TRINITY_DN3111_c2_g1_i1	No hit	28.7	5.5
285	TRINITY_DN739_c1_g2_i4	Large neutral amino acids transporter small subunit 2	28.4	5.3

286	TRINITY_DN54_c0_g1_i5	B(0,+)-type amino acid transporter 1-like	28.2	4.4
287	TRINITY_DN1113_c1_g2_i12	No hit	28.1	5.1
288	TRINITY_DN207_c0_g1_i29	Putative inorganic phosphate cotransporter isoform X1	28.0	6.9
289	TRINITY_DN4162_c0_g1_i18	No hit	27.9	3.4
290	TRINITY_DN4166_c0_g1_i1	No hit	27.9	1.9
291	TRINITY_DN7309_c0_g1_i14	Phospholipase D3-like isoform X3	27.9	5.8
292	TRINITY_DN4469_c0_g2_i6	No hit	27.7	1.6
293	TRINITY_DN8601_c0_g1_i25	Selenium-binding protein 1 isoform X3	27.6	7.3
294	TRINITY_DN2735_c0_g1_i7	Growth/differentiation factor 8	26.6	6.5
295	TRINITY_DN237_c0_g1_i2	Rap guanine nucleotide exchange factor 4 isoform X3	26.1	6.6
296	TRINITY_DN2140_c0_g1_i3	No hit	26.0	5.5
297	TRINITY_DN14747_c0_g2_i8	No hit	25.7	4.9
298	TRINITY_DN26889_c0_g1_i1	No hit	25.3	2.3
299	TRINITY_DN716_c0_g3_i17	Succinate-semialdehyde dehydrogenase, mitochondrial isoform X1	25.2	1.0
300	TRINITY_DN4469_c0_g2_i5	No hit	25.1	5.0
301	TRINITY_DN11241_c0_g1_i2	No hit	24.7	5.5
302	TRINITY_DN846_c0_g1_i12	No hit	24.6	7.7
303	TRINITY_DN756_c2_g1_i4	No hit	24.4	7.3
304	TRINITY_DN1678_c0_g3_i2	Ubiquitin-conjugating enzyme E2Q-like protein 1 isoform X4	24.4	6.0
305	TRINITY_DN2738_c0_g1_i188	Phosphoenolpyruvate carboxykinase [GTP]-like isoform X2	23.9	0.0
306	TRINITY_DN2181_c0_g3_i2	Facilitated trehalose transporter Tret1-like isoform X2	23.4	6.2
307	TRINITY_DN53645_c0_g1_i1	Venom serine carboxypeptidase-like	23.2	4.1
308	TRINITY_DN2399_c0_g1_i1	Plexin domain-containing protein 1	22.9	1.4
309	TRINITY_DN4166_c0_g1_i2	No hit	22.7	1.8
310	TRINITY_DN3297_c0_g2_i6	No hit	22.2	6.0
311	TRINITY_DN5196_c3_g1_i1	No hit	22.1	3.8
312	TRINITY_DN3949_c0_g1_i34	Protein-tyrosine sulfotransferase	21.8	4.4
313	TRINITY_DN49_c0_g1_i2	Facilitated trehalose transporter Tret1-like	21.5	4.7
314	TRINITY_DN3222_c0_g2_i2	Uncharacterized protein LOC106687487	21.3	7.1
315	TRINITY_DN4638_c0_g2_i3	Mannosyl-oligosaccharide alpha-1,2-mannosidase IA isoform X2	21.2	6.1
316	TRINITY_DN14747_c0_g2_i15	Abnormal cell migration protein 10	21.0	4.5
317	TRINITY_DN6178_c0_g1_i1	No hit	21.0	5.8
318	TRINITY_DN50123_c0_g1_i1	No hit	20.3	5.7
319	TRINITY_DN315_c1_g1_i2	SRSF protein kinase 3-like	20.0	5.2
320	TRINITY_DN147_c1_g1_i1	No hit	20.0	5.9
321	TRINITY_DN879_c1_g1_i6	Dihydropyrimidine dehydrogenase [NADP(+)]	19.9	2.1
322	TRINITY_DN687_c0_g1_i1	No hit	19.7	5.6
323	TRINITY_DN755_c0_g1_i6	No hit	19.6	0.0
324	TRINITY_DN8352_c0_g1_i4	No hit	19.6	4.6
325	TRINITY_DN405_c0_g1_i1	No hit	19.3	3.0
326	TRINITY_DN5_c4_g1_i1	No hit	19.1	4.4
327	TRINITY_DN3237_c0_g1_i4	No hit	18.7	3.6
328	TRINITY_DN11373_c0_g1_i1	No hit	18.5	4.8
329	TRINITY_DN2514_c0_g3_i2	No hit	18.2	5.0
330	TRINITY_DN132_c0_g1_i1	Uncharacterized protein LOC106682480	17.6	3.7
331	TRINITY_DN837_c0_g2_i1	No hit	17.5	3.4
332	TRINITY_DN3249_c1_g1_i1	Uncharacterized protein LOC106681816	17.2	4.3
333	TRINITY_DN3692_c0_g1_i22	No hit	16.9	5.0
334	TRINITY_DN1477_c0_g1_i1	No hit	16.5	2.6
335	TRINITY_DN9473_c0_g1_i2	No hit	16.1	4.8
336	TRINITY_DN53690_c0_g1_i1	No hit	16.0	4.3
337	TRINITY_DN33454_c0_g1_i1	No hit	16.0	0.4
338	TRINITY_DN20_c0_g1_i2	No hit	16.0	3.2
339	TRINITY_DN716_c0_g3_i3	Succinate-semialdehyde dehydrogenase, mitochondrial isoform X1	15.9	4.6
340	TRINITY_DN298_c0_g1_i1	No hit	15.8	4.2
341	TRINITY_DN3637_c0_g1_i1	No hit	15.4	1.8
342	TRINITY_DN22_c0_g2_i2	Uncharacterized protein LOC106681106	15.4	1.1
343	TRINITY_DN12082_c1_g1_i1	No hit	15.4	3.9
344	TRINITY_DN565_c0_g1_i7	No hit	15.2	4.5
345	TRINITY_DN2235_c11_g1_i1	No hit	15.1	4.0
346	TRINITY_DN1229_c3_g2_i3	Excitatory amino acid transporter 2 isoform X1	15.0	4.2
347	TRINITY_DN2346_c0_g1_i5	Solute carrier family 26 member 10-like isoform X1	14.8	2.4
348	TRINITY_DN2584_c0_g2_i3	Tensin-1, partial	14.2	2.2
349	TRINITY_DN42901_c0_g1_i1	No hit	14.1	0.2
350	TRINITY_DN8_c0_g1_i35	Secretin receptor-like	14.1	2.2
351	TRINITY_DN11444_c0_g1_i1	1-acyl-sn-glycerol-3-phosphate acyltransferase beta-like isoform X1	14.0	2.8
352	TRINITY_DN2_c0_g1_i67	Chloride channel protein 2 isoform X5	13.9	2.9
353	TRINITY_DN1176_c1_g4_i2	No hit	13.9	3.4
354	TRINITY_DN443_c0_g1_i2	Tubulointerstitial nephritis antigen-like	13.8	3.7
355	TRINITY_DN1177_c0_g1_i17	Serine/threonine-protein kinase OSR1-like isoform X3	13.8	4.1
356	TRINITY_DN385_c0_g1_i4	No hit	13.7	2.5
357	TRINITY_DN6652_c0_g1_i1	No hit	13.4	3.6

358	TRINITY_DN45_c0_g1_i6	Monocarboxylate transporter 10	13.4	3.2
359	TRINITY_DN4965_c0_g1_i1	No hit	13.4	3.0
360	TRINITY_DN1513_c0_g1_i11	Ankyrin repeat domain-containing protein 11 isoform X1	13.3	3.1
361	TRINITY_DN2586_c0_g1_i2	No hit	13.3	3.2
362	TRINITY_DN1798_c0_g1_i1	Amidophosphoribosyltransferase-like	13.3	3.9
363	TRINITY_DN3945_c0_g1_i6	Nostrin isoform X2	13.1	2.6
364	TRINITY_DN3552_c0_g1_i4	1,2-dihydroxy-3-keto-5-methylthiopentene dioxygenase	13.0	3.5
365	TRINITY_DN3105_c0_g1_i8	No hit	13.0	3.6
366	TRINITY_DN1202_c0_g1_i10	Protein sprint isoform X5	13.0	3.7
367	TRINITY_DN11085_c0_g1_i1	No hit	12.9	2.4
368	TRINITY_DN1467_c0_g1_i1	Solute carrier family 2, facilitated glucose transporter member 1 isoform X3	12.8	2.5
369	TRINITY_DN1582_c0_g1_i3	Acid phosphatase type 7-like	12.8	3.5
370	TRINITY_DN4703_c2_g1_i5	No hit	12.3	2.3
371	TRINITY_DN3104_c0_g1_i1	Uncharacterized protein LOC106680856, partial	12.1	0.2
372	TRINITY_DN781_c0_g1_i2	Sodium/potassium/calcium exchanger 4-like isoform X2	12.1	2.3
373	TRINITY_DN23_c0_g2_i4	No hit	12.1	3.7
374	TRINITY_DN1196_c0_g1_i3	Uncharacterized protein LOC106678897	12.0	3.1
375	TRINITY_DN5227_c0_g1_i1	Uncharacterized protein LOC106691212	12.0	2.5
376	TRINITY_DN1065_c1_g1_i1	No hit	12.0	2.5
377	TRINITY_DN2091_c0_g1_i4	Uncharacterized protein LOC106682324	11.9	2.9
378	TRINITY_DN786_c2_g2_i1	No hit	11.8	2.5
379	TRINITY_DN393_c0_g1_i3	Uncharacterized protein LOC106686172, partial	11.7	2.9
380	TRINITY_DN1833_c1_g1_i1	No hit	11.5	3.1
381	TRINITY_DN1622_c0_g1_i2	No hit	11.4	2.1
382	TRINITY_DN4262_c1_g1_i2	No hit	11.2	2.2
383	TRINITY_DN21494_c0_g1_i1	Uncharacterized protein LOC106686466	10.9	3.0
384	TRINITY_DN566_c3_g3_i1	No hit	10.8	1.8
385	TRINITY_DN5518_c0_g2_i8	Solute carrier family 25 member 44-like	10.6	0.2
386	TRINITY_DN502_c0_g4_i2	No hit	10.6	2.9
387	TRINITY_DN3752_c2_g1_i1	No hit	10.5	1.1
388	TRINITY_DN1810_c0_g1_i11	Putative cystathionine gamma-lyase 2	10.4	2.1
389	TRINITY_DN1994_c3_g1_i1	No hit	10.2	2.8
390	TRINITY_DN2026_c0_g1_i12	No hit	10.1	1.4
391	TRINITY_DN3916_c0_g1_i8	No hit	10.0	2.1
392	TRINITY_DN393_c0_g1_i7	Uncharacterized protein LOC106686172, partial	10.0	2.6
393	TRINITY_DN355_c1_g2_i2	No hit	9.9	1.3
394	TRINITY_DN8728_c0_g2_i1	Uncharacterized protein LOC106678977	9.9	2.6
395	TRINITY_DN2193_c0_g1_i1	No hit	9.8	1.6
396	TRINITY_DN1873_c0_g2_i1	Protein atonal homolog 8 isoform X1	9.8	1.8
397	TRINITY_DN3606_c0_g1_i4	No hit	9.8	2.2
398	TRINITY_DN6972_c0_g1_i5	Uncharacterized protein LOC106683901	9.6	0.0
399	TRINITY_DN8601_c0_g1_i8	Selenium-binding protein 1 isoform X2	9.4	1.3
400	TRINITY_DN15386_c0_g2_i1	No hit	9.3	1.9
401	TRINITY_DN7997_c0_g1_i1	No hit	9.3	1.1
402	TRINITY_DN9444_c0_g1_i1	No hit	9.3	1.0
403	TRINITY_DN3753_c1_g2_i1	No hit	9.2	1.9
404	TRINITY_DN1982_c0_g1_i1	Esterase E4-like	9.2	2.0
405	TRINITY_DN1431_c0_g2_i12	Tropomodulin-1 isoform X5	9.2	0.0
406	TRINITY_DN4316_c0_g1_i2	Lipase 1	9.0	0.9
407	TRINITY_DN124_c0_g1_i42	Uncharacterized protein LOC106677625	9.0	1.7
408	TRINITY_DN52_c6_g1_i1	No hit	8.8	1.6
409	TRINITY_DN2175_c1_g1_i13	Protein cycle isoform X3	8.7	1.4
410	TRINITY_DN4316_c0_g1_i6	Lipase 1	8.7	0.2
411	TRINITY_DN9364_c0_g1_i2	Zinc transporter ZIP8	8.5	2.1
412	TRINITY_DN6178_c0_g3_i16	No hit	8.5	2.2
413	TRINITY_DN1351_c0_g1_i20	No hit	8.4	0.0
414	TRINITY_DN587_c2_g1_i3	No hit	8.2	1.5
415	TRINITY_DN14885_c0_g1_i1	No hit	8.2	0.5
416	TRINITY_DN11919_c0_g3_i1	No hit	8.2	1.1
417	TRINITY_DN26167_c0_g1_i1	No hit	8.1	1.9
418	TRINITY_DN19512_c0_g1_i1	No hit	8.1	0.7
419	TRINITY_DN34944_c0_g1_i1	Oxysterol-binding protein-related protein 9-like	8.1	1.6
420	TRINITY_DN2019_c0_g1_i4	cium/calmodulin-dependent 3',5'-cyclic nucleotide phosphodiesterase 1-like isoform	8.0	1.6
421	TRINITY_DN11322_c0_g1_i6	No hit	7.8	0.7
422	TRINITY_DN2004_c0_g1_i4	Sex peptide receptor	7.7	1.5
423	TRINITY_DN5342_c0_g1_i1	Putative inorganic phosphate cotransporter isoform X1	7.7	2.0
424	TRINITY_DN2847_c1_g1_i7	Alpha,alpha-trehalose-phosphate synthase [UDP-forming] isoform X1	7.7	1.2
425	TRINITY_DN3237_c0_g1_i3	No hit	7.7	2.0
426	TRINITY_DN1732_c4_g1_i1	No hit	7.6	1.5
427	TRINITY_DN43482_c0_g1_i1	No hit	7.6	1.2
428	TRINITY_DN2702_c0_g2_i4	Putative cysteine proteinase CG12163 isoform X1	7.5	1.4
429	TRINITY_DN207_c0_g1_i32	Cytochrome b5-related protein isoform X2	7.5	1.8

430	TRINITY_DN17898_c2_g1_i1	No hit	7.3	1.4
431	TRINITY_DN10110_c0_g1_i2	No hit	7.2	1.9
432	TRINITY_DN2423_c0_g1_i1	No hit	7.1	1.8
433	TRINITY_DN41579_c0_g1_i1	No hit	7.1	0.5
434	TRINITY_DN20756_c0_g3_i8	Uncharacterized protein LOC106690952	7.1	1.6
435	TRINITY_DN2396_c4_g1_i1	No hit	7.0	0.9
436	TRINITY_DN144_c0_g1_i3	Uncharacterized protein LOC106687460 isoform X2	6.9	1.8
437	TRINITY_DN2298_c1_g1_i1	No hit	6.8	1.0
438	TRINITY_DN876_c0_g1_i11	Facilitated trehalose transporter Tret1	6.7	1.2
439	TRINITY_DN2962_c0_g2_i22	No hit	6.6	1.4
440	TRINITY_DN13446_c0_g2_i1	No hit	6.6	1.5
441	TRINITY_DN1545_c1_g1_i25	No hit	6.6	0.3
442	TRINITY_DN11322_c0_g1_i4	Uncharacterized protein LOC106688805	6.5	1.3
443	TRINITY_DN1229_c3_g2_i7	Excitatory amino acid transporter 2 isoform X1	6.5	1.9
444	TRINITY_DN231_c0_g1_i5	Balbiani ring protein 3	6.5	1.4
445	TRINITY_DN207_c0_g1_i20	Putative inorganic phosphate cotransporter isoform X2	6.4	1.5
446	TRINITY_DN4055_c0_g1_i3	Ribosomal protein S6 kinase alpha-5	6.4	1.7
447	TRINITY_DN49_c0_g1_i10	Facilitated trehalose transporter Tret1-like	6.4	0.0
448	TRINITY_DN237_c0_g1_i19	No hit	6.3	1.3
449	TRINITY_DN1038_c0_g2_i1	No hit	6.2	1.4
450	TRINITY_DN3597_c0_g1_i5	Phospholipase B1, membrane-associated	6.1	1.2
451	TRINITY_DN1893_c0_g2_i1	No hit	6.1	1.0
452	TRINITY_DN4204_c0_g1_i1	Protein timeless	6.0	1.6
453	TRINITY_DN56793_c0_g1_i1	No hit	6.0	0.5
454	TRINITY_DN20756_c0_g3_i5	Uncharacterized protein LOC106690952	5.9	1.3
455	TRINITY_DN1177_c0_g1_i12	No hit	5.8	1.0
456	TRINITY_DN4048_c0_g1_i4	PQ-loop repeat-containing protein 1 isoform X3	5.8	0.2
457	TRINITY_DN909_c0_g1_i1	Protein spire	5.7	0.2
458	TRINITY_DN50934_c0_g1_i1	No hit	5.6	0.6
459	TRINITY_DN3653_c0_g2_i4	No hit	5.5	0.6
460	TRINITY_DN7380_c1_g1_i3	No hit	5.5	1.2
461	TRINITY_DN7474_c0_g4_i4	Epidermal retinol dehydrogenase 2-like isoform X1	5.5	1.0
462	TRINITY_DN177_c0_g2_i1	No hit	5.5	0.5
463	TRINITY_DN23073_c0_g1_i1	No hit	5.4	0.7
464	TRINITY_DN706_c2_g2_i3	No hit	5.4	0.6
465	TRINITY_DN3565_c0_g1_i11	Putative phospholipase B-like lamina ancestor	5.3	1.2
466	TRINITY_DN8393_c0_g1_i1	No hit	5.3	0.7
467	TRINITY_DN3167_c1_g2_i1	No hit	5.2	1.0
468	TRINITY_DN1431_c0_g2_i17	Tropomodulin-1 isoform X6	5.1	0.0
469	TRINITY_DN10630_c0_g1_i27	Fatty-acid amide hydrolase 2-A-like	5.1	1.3
470	TRINITY_DN76_c0_g2_i2	No hit	5.1	0.6
471	TRINITY_DN15076_c0_g2_i1	No hit	5.0	0.0
472	TRINITY_DN194_c0_g1_i52	Glutamate synthase [NADH], amyloplastic isoform X1	5.0	1.0
473	TRINITY_DN2381_c0_g1_i13	Transportin-3	5.0	0.0
474	TRINITY_DN7891_c0_g2_i3	No hit	4.9	0.9
475	TRINITY_DN3692_c0_g1_i36	Lysosomal aspartic protease	4.9	0.3
476	TRINITY_DN4671_c0_g1_i5	No hit	4.8	0.8
477	TRINITY_DN7187_c1_g1_i1	No hit	4.7	1.2
478	TRINITY_DN11124_c0_g1_i1	Protein artichoke	4.7	1.0
479	TRINITY_DN4808_c3_g1_i1	No hit	4.7	0.4
480	TRINITY_DN2896_c0_g1_i1	No hit	4.5	1.1
481	TRINITY_DN691_c1_g1_i1	No hit	4.5	1.1
482	TRINITY_DN1560_c0_g1_i3	No hit	4.4	0.5
483	TRINITY_DN4291_c0_g1_i4	Neuropeptides capa receptor-like	4.4	1.1
484	TRINITY_DN53944_c0_g1_i1	No hit	4.3	0.6
485	TRINITY_DN4143_c0_g1_i10	ATP-dependent RNA helicase DDX42	4.3	0.8
486	TRINITY_DN1967_c1_g1_i7	ne/threonine-protein phosphatase 2A regulatory subunit B'' subunit beta-like isoforn	4.3	0.8
487	TRINITY_DN622_c0_g1_i13	No hit	4.3	0.6
488	TRINITY_DN4192_c0_g1_i1	No hit	4.2	0.9
489	TRINITY_DN2623_c0_g1_i10	Tyrosine-protein kinase CSK	4.1	0.0
490	TRINITY_DN8398_c0_g2_i3	No hit	4.1	0.9
491	TRINITY_DN3063_c0_g1_i9	Cathepsin B-like	4.0	0.9
492	TRINITY_DN4030_c3_g1_i1	No hit	4.0	0.9
493	TRINITY_DN2906_c0_g1_i4	CAMP-dependent protein kinase type I regulatory subunit	4.0	0.8
494	TRINITY_DN12174_c0_g1_i2	No hit	4.0	0.9
495	TRINITY_DN26052_c0_g1_i1	No hit	4.0	0.6
496	TRINITY_DN1448_c1_g4_i3	Cathepsin B-like isoform X2	3.9	0.7
497	TRINITY_DN242_c0_g2_i1	Nose resistant to fluoxetine protein 6-like	3.9	0.9
498	TRINITY_DN26974_c0_g1_i2	No hit	3.9	0.6
499	TRINITY_DN315_c1_g1_i4	SRSF protein kinase 3-like	3.9	0.8
500	TRINITY_DN3287_c1_g1_i3	Probable uridine nucleosidase 1	3.9	0.6
501	TRINITY_DN2314_c1_g1_i1	No hit	3.8	0.5

502	TRINITY_DN8398_c0_g2_i4	Protein FAM210A	3.7	0.5
503	TRINITY_DN2146_c0_g1_i4	General vesicular transport factor p115 isoform X1	3.6	0.0
504	TRINITY_DN629_c0_g1_i2	LOW QUALITY PROTEIN: calpain-1 catalytic subunit-like	3.6	0.8
505	TRINITY_DN3844_c0_g1_i6	Uncharacterized protein LOC106677424	3.5	0.0
506	TRINITY_DN2175_c1_g1_i16	Protein cycle isoform X5	3.5	0.1
507	TRINITY_DN1737_c0_g1_i1	No hit	3.5	0.8
508	TRINITY_DN393_c0_g1_i51	Uncharacterized protein LOC106686172, partial	3.3	0.7
509	TRINITY_DN1483_c0_g1_i3	Protein FAM214A isoform X1	3.3	0.0
510	TRINITY_DN764_c0_g1_i3	Glutathione hydrolase 1 proenzyme isoform X1	3.2	0.6
511	TRINITY_DN22_c2_g1_i1	No hit	3.2	0.5
512	TRINITY_DN3043_c0_g1_i5	No hit	3.2	0.6
513	TRINITY_DN37818_c0_g1_i1	No hit	3.0	0.4
514	TRINITY_DN3008_c0_g1_i58	Protein fuzzy homolog isoform X1	2.9	0.0
515	TRINITY_DN1189_c0_g1_i28	No hit	2.8	0.6

[1] In the order of average expression levels in the crypts.

[2] Average expression levels of four individuals.

[3] Significantly higher expression in the crypts than in the main tract (FDR q < 0.01).

Table S6. Expression levels of differentially expressed insect genes upregulated in the adult main tract of *P. stali*.

Rank [1]	Contig ID	Description	TPM [2]	
			Crypts	Main tract [3]
1	TRINITY_DN341_c0_g1_i4	No hit	2496.1	43250.9
2	TRINITY_DN441_c1_g2_i11	No hit	1090.3	42070.3
3	TRINITY_DN2568_c0_g1_i1	No hit	1121.5	39508.5
4	TRINITY_DN2374_c0_g1_i3	Uncharacterized protein LOC106678109	2359.6	21728.4
5	TRINITY_DN441_c1_g2_i3	No hit	218.8	17401.4
6	TRINITY_DN4566_c0_g1_i1	Uncharacterized protein LOC106692511	185.9	6365.2
7	TRINITY_DN179_c1_g1_i1	Uncharacterized protein LOC106680650	229.8	5827.7
8	TRINITY_DN3693_c0_g1_i3	No hit	362.5	5557.7
9	TRINITY_DN2125_c0_g1_i1	Cathepsin L1-like isoform X3	156.3	5400.7
10	TRINITY_DN5750_c0_g1_i1	No hit	103.5	4910.2
11	TRINITY_DN6261_c0_g1_i2	Uncharacterized protein LOC106692576	63.8	3967.6
12	TRINITY_DN14573_c0_g1_i1	Cathepsin B-like	75.6	3910.1
13	TRINITY_DN6818_c0_g1_i1	Uncharacterized protein LOC106685942	307.3	3155.0
14	TRINITY_DN441_c0_g1_i1	No hit	571.2	3052.0
15	TRINITY_DN2734_c0_g1_i1	No hit	54.7	2743.3
16	TRINITY_DN32999_c0_g1_i1	Uncharacterized protein LOC106685944	60.2	2731.2
17	TRINITY_DN1345_c0_g1_i6	Uncharacterized protein LOC106690248	75.8	2685.6
18	TRINITY_DN46788_c0_g1_i1	Acyl-CoA-binding protein homolog	125.4	2661.5
19	TRINITY_DN36108_c0_g1_i1	No hit	34.5	2581.2
20	TRINITY_DN3347_c0_g1_i2	Uncharacterized protein LOC112210696	70.3	2122.3
21	TRINITY_DN3260_c0_g1_i1	Uncharacterized protein LOC106689981	310.8	2083.0
22	TRINITY_DN441_c1_g1_i1	No hit	41.1	2029.0
23	TRINITY_DN35973_c0_g1_i1	No hit	27.4	1681.2
24	TRINITY_DN7389_c0_g1_i2	No hit	228.7	1536.2
25	TRINITY_DN786_c3_g1_i1	Cathepsin L1 isoform X1	145.7	1459.1
26	TRINITY_DN7917_c0_g1_i2	No hit	57.3	1400.8
27	TRINITY_DN30417_c0_g1_i1	No hit	20.4	1167.5
28	TRINITY_DN1556_c0_g1_i1	No hit	192.1	1146.8
29	TRINITY_DN44596_c0_g1_i1	Probable salivary secreted peptide	21.1	1111.0
30	TRINITY_DN36827_c0_g1_i1	No hit	13.1	1087.9
31	TRINITY_DN14573_c0_g2_i1	LOW QUALITY PROTEIN: uncharacterized protein LOC106688843	30.7	1064.4
32	TRINITY_DN1554_c1_g1_i1	Cathepsin L1	53.7	943.6
33	TRINITY_DN55185_c0_g1_i1	Uncharacterized protein LOC106685943	42.9	930.5
34	TRINITY_DN9923_c0_g1_i2	Multicystatin-like	20.7	922.7
35	TRINITY_DN14988_c0_g1_i1	No hit	47.4	918.4
36	TRINITY_DN15747_c0_g1_i1	Aspartic proteinase A3-like	19.6	861.7
37	TRINITY_DN8710_c0_g2_i2	Laccase-4	17.4	859.5
38	TRINITY_DN789_c0_g2_i4	Acyl-CoA-binding domain-containing protein 7-like	74.4	793.1
39	TRINITY_DN50111_c0_g1_i1	Probable salivary secreted peptide	11.0	735.9
40	TRINITY_DN9176_c1_g1_i1	No hit	3.3	701.2
41	TRINITY_DN2992_c0_g1_i4	Uncharacterized protein LOC106686776	20.6	700.7
42	TRINITY_DN9863_c0_g1_i1	Natterin-4 isoform X1	47.5	648.5
43	TRINITY_DN10486_c0_g1_i1	Uncharacterized protein LOC106687672	31.8	641.1
44	TRINITY_DN3736_c0_g3_i1	Thymosin beta isoform X2	40.3	628.6
45	TRINITY_DN25545_c0_g1_i1	Uncharacterized protein LOC106689449 isoform X1	13.6	615.4
46	TRINITY_DN36659_c0_g1_i1	Uncharacterized protein LOC106685533	77.1	601.9
47	TRINITY_DN46418_c0_g1_i1	Probable salivary secreted peptide	14.7	595.0
48	TRINITY_DN1345_c0_g1_i3	Uncharacterized protein LOC106687435	12.4	565.6
49	TRINITY_DN3330_c0_g1_i2	Trypsin-1-like	20.6	563.2
50	TRINITY_DN7901_c0_g1_i1	No hit	22.1	560.3
51	TRINITY_DN1462_c0_g1_i1	No hit	8.9	556.7
52	TRINITY_DN5968_c0_g1_i1	Uncharacterized protein LOC106682699 isoform X1	11.9	527.4
53	TRINITY_DN6707_c0_g3_i4	Aminopeptidase N-like	14.8	495.3
54	TRINITY_DN55140_c0_g1_i1	No hit	11.7	485.1
55	TRINITY_DN46371_c0_g1_i1	No hit	13.1	471.6
56	TRINITY_DN1283_c0_g1_i2	Probable citrate synthase 2, mitochondrial isoform X2	93.7	461.6
57	TRINITY_DN5257_c0_g2_i1	Uncharacterized protein LOC106685509	11.4	448.5
58	TRINITY_DN50908_c0_g1_i1	Uncharacterized protein LOC112210405	9.0	446.6
59	TRINITY_DN34_c0_g1_i3	L-rhamnose-binding lectin CSL3	64.9	434.4
60	TRINITY_DN24582_c0_g1_i1	No hit	9.6	423.5
61	TRINITY_DN2879_c0_g1_i1	Uncharacterized protein LOC106691321	26.1	403.9
62	TRINITY_DN5257_c0_g1_i1	Uncharacterized protein LOC106685509	9.3	396.8
63	TRINITY_DN10039_c0_g1_i1	Uncharacterized protein LOC106686307	6.3	394.7
64	TRINITY_DN7955_c0_g1_i1	No hit	6.3	380.9
65	TRINITY_DN5112_c0_g1_i5	GATA type zinc finger protein asd-4-like	9.0	369.3
66	TRINITY_DN38775_c0_g1_i1	Putative serine protease K12H4.7 isoform X1	10.3	366.4
67	TRINITY_DN3695_c0_g1_i1	No hit	9.7	365.0
68	TRINITY_DN42841_c0_g1_i1	Cathepsin L1-like	8.7	363.8
69	TRINITY_DN1310_c0_g1_i1	No hit	30.4	357.9
70	TRINITY_DN3926_c0_g1_i1	Cytochrome c oxidase subunit 6B-like	16.2	356.5
71	TRINITY_DN12917_c0_g1_i1	No hit	9.6	349.3
72	TRINITY_DN9081_c0_g1_i1	NPC intracellular cholesterol transporter 2 homolog a-like	14.0	335.2
73	TRINITY_DN833_c0_g1_i4	Tubulin alpha-1 chain-like	50.2	327.6

74	TRINITY_DN1051_c2_g6_i1	No hit	9.9	327.0
75	TRINITY_DN4917_c0_g1_i1	No hit	10.0	322.4
76	TRINITY_DN10075_c0_g1_i1	Fibrinolytic enzyme, isozyme C	50.0	320.3
77	TRINITY_DN27124_c0_g1_i1	No hit	21.1	291.6
78	TRINITY_DN1345_c0_g1_i4	Uncharacterized protein LOC106687435	3.6	283.4
79	TRINITY_DN2917_c0_g1_i1	No hit	15.1	276.2
80	TRINITY_DN13246_c0_g1_i2	GTP cyclohydrolase 1 isoform X1	27.9	276.0
81	TRINITY_DN2832_c1_g1_i1	No hit	24.6	273.3
82	TRINITY_DN882_c0_g1_i1	No hit	5.8	267.7
83	TRINITY_DN9852_c0_g3_i3	Cardioactive peptide	8.5	263.2
84	TRINITY_DN3605_c0_g1_i1	No hit	9.2	257.1
85	TRINITY_DN2131_c2_g1_i1	No hit	4.0	256.1
86	TRINITY_DN2329_c0_g1_i6	Procardosin-A	7.5	254.6
87	TRINITY_DN49824_c0_g1_i1	Legumain-like	6.2	246.7
88	TRINITY_DN13964_c0_g1_i1	No hit	6.9	240.0
89	TRINITY_DN4023_c0_g1_i7	No hit	4.2	237.4
90	TRINITY_DN42448_c0_g1_i1	Venom carboxylesterase-6-like	6.6	236.1
91	TRINITY_DN19854_c0_g1_i1	No hit	3.7	235.4
92	TRINITY_DN15137_c0_g1_i1	Uncharacterized protein LOC106692736	8.4	231.7
93	TRINITY_DN40877_c0_g1_i1	Short neuropeptide F-like	17.8	230.2
94	TRINITY_DN349_c0_g2_i1	No hit	32.9	227.9
95	TRINITY_DN6767_c0_g2_i1	No hit	31.1	220.9
96	TRINITY_DN441_c0_g2_i2	No hit	34.4	219.1
97	TRINITY_DN47339_c0_g1_i1	No hit	6.5	217.8
98	TRINITY_DN6767_c0_g1_i1	No hit	16.0	210.1
99	TRINITY_DN1636_c0_g1_i1	Clavesin-1	23.1	208.6
100	TRINITY_DN46237_c0_g1_i1	No hit	3.9	206.2
101	TRINITY_DN4224_c0_g1_i4	No hit	7.2	205.2
102	TRINITY_DN2556_c0_g1_i3	Reticulon-4-like isoform X1	5.6	200.8
103	TRINITY_DN8184_c0_g1_i3	Arylsulfatase B-like	7.1	193.4
104	TRINITY_DN8792_c1_g1_i1	No hit	3.9	192.1
105	TRINITY_DN38070_c0_g1_i1	No hit	21.8	191.3
106	TRINITY_DN46657_c0_g1_i1	No hit	3.7	188.9
107	TRINITY_DN6104_c0_g1_i1	No hit	11.7	188.5
108	TRINITY_DN896_c0_g1_i4	Uncharacterized protein LOC106693016	7.1	188.0
109	TRINITY_DN18226_c0_g1_i1	WSC domain-containing protein ARB 07867 isoform X1	2.5	187.3
110	TRINITY_DN29861_c0_g1_i1	Uncharacterized protein LOC106682528	19.6	184.6
111	TRINITY_DN1072_c1_g1_i1	Aldose reductase-related protein 2-like isoform X1	12.7	184.5
112	TRINITY_DN7341_c0_g2_i1	No hit	10.2	181.9
113	TRINITY_DN2737_c0_g1_i1	No hit	13.5	181.7
114	TRINITY_DN16965_c0_g1_i3	No hit	29.1	175.8
115	TRINITY_DN7823_c0_g1_i1	No hit	3.0	174.1
116	TRINITY_DN2574_c0_g2_i9	Repressed by EFG1 protein 1	9.8	173.8
117	TRINITY_DN12803_c2_g1_i3	UDP-glucuronosyltransferase 2B7-like	30.1	169.6
118	TRINITY_DN1011_c0_g1_i2	No hit	22.2	169.5
119	TRINITY_DN2915_c0_g1_i3	Chitinase-like protein EN03	21.1	162.6
120	TRINITY_DN7106_c2_g1_i2	Probable cytochrome P450 6a13	4.3	160.1
121	TRINITY_DN7106_c2_g1_i1	No hit	2.0	159.0
122	TRINITY_DN7066_c1_g1_i1	No hit	3.5	157.9
123	TRINITY_DN8792_c1_g2_i1	No hit	2.9	157.8
124	TRINITY_DN789_c0_g2_i6	Acyl-CoA-binding domain-containing protein 7-like	9.3	154.5
125	TRINITY_DN3049_c0_g1_i1	Mid1-interacting protein 1-B	25.5	152.9
126	TRINITY_DN3533_c0_g1_i1	Translocator protein	17.4	151.5
127	TRINITY_DN54058_c0_g1_i1	Dihydrofolate reductase	7.1	149.8
128	TRINITY_DN933_c0_g1_i1	Prestin-like	6.7	149.1
129	TRINITY_DN8108_c0_g1_i5	No hit	5.7	148.7
130	TRINITY_DN242_c2_g2_i1	Homeobox protein Nkx-2.2a-like	4.8	148.1
131	TRINITY_DN663_c0_g1_i15	No hit	15.1	147.5
132	TRINITY_DN5203_c0_g1_i1	No hit	3.0	146.9
133	TRINITY_DN2122_c0_g1_i1	2-acylglycerol O-acyltransferase 1-like	5.9	146.8
134	TRINITY_DN717_c0_g1_i2	Regucalcin-like	8.3	146.5
135	TRINITY_DN12314_c1_g1_i1	Titin isoform X4	5.7	144.5
136	TRINITY_DN4459_c0_g1_i1	Uncharacterized protein LOC106690010 isoform X4	3.2	142.2
137	TRINITY_DN28747_c0_g1_i1	Uncharacterized protein LOC106682797	2.0	140.8
138	TRINITY_DN17385_c0_g1_i1	No hit	2.8	137.1
139	TRINITY_DN40691_c0_g1_i1	No hit	3.7	136.3
140	TRINITY_DN34110_c0_g1_i1	No hit	2.5	135.4
141	TRINITY_DN46874_c0_g1_i1	No hit	3.5	134.2
142	TRINITY_DN29807_c0_g1_i1	No hit	0.4	133.9
143	TRINITY_DN3032_c0_g1_i1	No hit	4.0	132.3
144	TRINITY_DN49990_c0_g1_i1	Uncharacterized protein LOC106689931	4.0	130.6
145	TRINITY_DN3168_c0_g1_i1	No hit	12.4	129.6
146	TRINITY_DN8137_c0_g1_i1	Uncharacterized protein LOC106679388	1.1	127.6
147	TRINITY_DN21431_c1_g1_i1	No hit	17.1	127.3
148	TRINITY_DN7230_c0_g1_i50	Extended synaptotagmin-2-B isoform X2	15.4	126.2
149	TRINITY_DN31274_c0_g1_i1	No hit	6.2	125.3
150	TRINITY_DN10544_c0_g1_i1	No hit	1.4	124.4

151	TRINITY_DN355_c6_g1_i1	1,5-anhydro-D-fructose reductase-like	18.7	123.8
152	TRINITY_DN7028_c0_g1_i1	Protein lifeguard 1	19.3	123.7
153	TRINITY_DN46760_c0_g1_i1	No hit	2.1	122.9
154	TRINITY_DN2748_c0_g1_i1	No hit	1.9	122.1
155	TRINITY_DN3773_c0_g1_i2	No hit	4.5	121.5
156	TRINITY_DN349_c0_g1_i5	Pancreatic lipase-related protein 2-like	19.7	119.7
157	TRINITY_DN44453_c0_g1_i1	No hit	2.2	118.8
158	TRINITY_DN8792_c0_g1_i3	No hit	2.7	117.5
159	TRINITY_DN42401_c0_g1_i1	Myosin light chain alkali	22.0	116.2
160	TRINITY_DN2908_c3_g4_i1	Probable cytochrome P450 6d5	4.0	115.7
161	TRINITY_DN8710_c0_g1_i1	No hit	1.9	113.9
162	TRINITY_DN56278_c0_g1_i1	No hit	3.5	113.9
163	TRINITY_DN42414_c0_g1_i1	Maltase A1-like	2.4	112.0
164	TRINITY_DN2484_c0_g1_i1	No hit	6.3	111.5
165	TRINITY_DN4572_c0_g1_i2	No hit	4.7	110.3
166	TRINITY_DN42940_c0_g1_i1	No hit	1.8	109.6
167	TRINITY_DN875_c1_g1_i1	No hit	19.4	108.9
168	TRINITY_DN2106_c0_g1_i8	Aspartic proteinase A3 isoform X2	2.3	106.4
169	TRINITY_DN1686_c2_g4_i7	Tubulin-specific chaperone D	2.5	106.4
170	TRINITY_DN287_c0_g1_i1	Alpha-glucosidase-like	3.1	104.3
171	TRINITY_DN1710_c0_g1_i32	Tropomyosin isoform X11	18.8	103.4
172	TRINITY_DN38013_c0_g1_i1	No hit	0.0	102.7
173	TRINITY_DN35284_c0_g1_i1	No hit	2.8	102.4
174	TRINITY_DN12711_c0_g1_i1	Uncharacterized protein LOC106688696	6.6	101.3
175	TRINITY_DN3044_c1_g1_i1	Insulin-like growth factor-binding protein complex acid labile subunit	3.2	99.6
176	TRINITY_DN2015_c0_g2_i2	No hit	13.8	99.4
177	TRINITY_DN6727_c0_g1_i2	No hit	11.2	97.1
178	TRINITY_DN8108_c0_g1_i1	No hit	3.4	96.7
179	TRINITY_DN5841_c0_g1_i1	Cytochrome P450 6a2-like	3.5	95.5
180	TRINITY_DN4284_c0_g1_i1	No hit	6.6	94.6
181	TRINITY_DN4366_c0_g1_i1	Myophilin	17.2	94.4
182	TRINITY_DN465_c0_g1_i1	No hit	7.5	94.2
183	TRINITY_DN2234_c0_g1_i1	Serine/threonine-protein phosphatase 6 regulatory ankyrin repeat subunit B-like	1.6	94.1
184	TRINITY_DN6850_c0_g1_i1	No hit	7.9	93.4
185	TRINITY_DN2026_c0_g1_i16	No hit	5.4	91.8
186	TRINITY_DN4493_c0_g1_i1	No hit	3.8	91.2
187	TRINITY_DN1872_c0_g1_i1	No hit	5.5	88.8
188	TRINITY_DN2329_c0_g1_i8	No hit	1.4	88.7
189	TRINITY_DN4224_c0_g1_i3	No hit	6.8	88.2
190	TRINITY_DN3695_c0_g1_i2	No hit	0.3	88.1
191	TRINITY_DN1121_c0_g1_i2	No hit	5.2	87.1
192	TRINITY_DN51219_c0_g1_i1	Uncharacterized protein LOC106687433	9.8	87.0
193	TRINITY_DN48441_c0_g1_i1	No hit	1.8	86.1
194	TRINITY_DN1015_c0_g3_i1	Uncharacterized protein LOC106677904	4.0	83.9
195	TRINITY_DN25496_c0_g1_i1	Spondin-1	2.9	83.7
196	TRINITY_DN35239_c0_g1_i1	Beta-mannosidase	4.3	83.7
197	TRINITY_DN4224_c0_g1_i7	No hit	2.6	82.9
198	TRINITY_DN4703_c0_g3_i1	No hit	0.5	82.5
199	TRINITY_DN35067_c0_g1_i1	No hit	12.4	82.0
200	TRINITY_DN905_c0_g1_i3	Cathepsin L1	7.5	81.9
201	TRINITY_DN441_c1_g2_i1	No hit	1.2	81.9
202	TRINITY_DN6154_c0_g2_i1	1,5-anhydro-D-fructose reductase-like isoform X2	3.1	81.6
203	TRINITY_DN23250_c0_g1_i1	No hit	0.9	81.5
204	TRINITY_DN26236_c0_g1_i2	Uncharacterized protein LOC106679595	1.8	80.4
205	TRINITY_DN50_c0_g2_i1	Venom carboxylesterase-6	2.8	79.3
206	TRINITY_DN540_c0_g1_i1	Actin-binding Rho-activating protein-like	6.0	77.5
207	TRINITY_DN9448_c0_g1_i1	No hit	9.2	77.3
208	TRINITY_DN31730_c0_g1_i1	No hit	1.0	76.9
209	TRINITY_DN4224_c0_g1_i1	No hit	3.6	76.9
210	TRINITY_DN12314_c0_g1_i1	Titin isoform X5	2.6	76.8
211	TRINITY_DN16965_c0_g1_i1	No hit	2.2	76.5
212	TRINITY_DN4023_c0_g1_i5	No hit	2.9	75.4
213	TRINITY_DN14640_c0_g1_i1	Uricase	1.2	75.2
214	TRINITY_DN16571_c0_g1_i1	No hit	0.0	74.8
215	TRINITY_DN905_c0_g1_i15	Cathepsin L1	2.8	74.3
216	TRINITY_DN12807_c0_g1_i1	No hit	3.0	73.7
217	TRINITY_DN56378_c0_g1_i1	No hit	6.8	73.4
218	TRINITY_DN13228_c0_g1_i1	No hit	1.9	72.9
219	TRINITY_DN626_c0_g3_i1	4-aminobutyrate aminotransferase, mitochondrial	4.2	72.0
220	TRINITY_DN11243_c1_g3_i2	UDP-glucuronosyltransferase 2B23-like isoform X1	4.7	71.7
221	TRINITY_DN4058_c0_g1_i1	No hit	1.1	71.5
222	TRINITY_DN2015_c0_g1_i1	Uncharacterized protein LOC106677816	10.1	71.4
223	TRINITY_DN6265_c0_g1_i2	Uncharacterized protein LOC106692099	5.0	71.3
224	TRINITY_DN40556_c0_g1_i1	No hit	3.4	71.0
225	TRINITY_DN33044_c0_g1_i1	No hit	1.5	67.3
226	TRINITY_DN3828_c0_g1_i1	No hit	0.7	67.0
227	TRINITY_DN29959_c0_g1_i1	No hit	1.0	66.7

228	TRINITY_DN1903_c1_g1_i4	Uncharacterized protein LOC106684378 isoform X1	10.5	66.5
229	TRINITY_DN16571_c0_g2_i1	No hit	3.4	66.4
230	TRINITY_DN9380_c5_g1_i1	No hit	1.2	65.9
231	TRINITY_DN38754_c0_g1_i1	No hit	2.7	65.8
232	TRINITY_DN33677_c0_g1_i1	Protein singed	6.5	64.7
233	TRINITY_DN1903_c1_g1_i6	No hit	1.9	63.8
234	TRINITY_DN46250_c0_g1_i1	Uncharacterized protein LOC106691510	1.9	63.3
235	TRINITY_DN2689_c0_g2_i2	Non-lysosomal glucosylceramidase isoform X2	13.7	63.3
236	TRINITY_DN3330_c0_g1_i4	Trypsin-1-like	1.8	62.7
237	TRINITY_DN1071_c0_g1_i28	No hit	5.9	62.6
238	TRINITY_DN9159_c0_g1_i2	Uncharacterized protein LOC106681158	6.7	62.4
239	TRINITY_DN6412_c3_g1_i1	Uncharacterized protein LOC106684271	1.6	62.4
240	TRINITY_DN1072_c0_g1_i9	1,5-anhydro-D-fructose reductase	4.4	61.9
241	TRINITY_DN40218_c0_g1_i1	No hit	4.8	61.7
242	TRINITY_DN9855_c0_g1_i1	No hit	1.4	61.4
243	TRINITY_DN182_c0_g1_i2	Uncharacterized protein LOC106689817	7.0	61.0
244	TRINITY_DN5553_c0_g1_i1	Ras-like GTP-binding protein Rho1	3.5	60.5
245	TRINITY_DN13655_c0_g1_i1	No hit	4.0	60.3
246	TRINITY_DN51710_c0_g1_i1	No hit	2.5	60.0
247	TRINITY_DN11152_c0_g2_i1	No hit	0.4	59.9
248	TRINITY_DN2084_c0_g1_i4	MFS-type transporter SLC18B1 isoform X1	2.9	59.7
249	TRINITY_DN1906_c0_g1_i2	Transient receptor potential channel pyrexia-like	1.8	59.6
250	TRINITY_DN2333_c3_g1_i1	No hit	0.6	59.6
251	TRINITY_DN4715_c0_g1_i8	Tubulin alpha-1A chain-like	4.6	59.5
252	TRINITY_DN26344_c0_g3_i1	Uncharacterized protein LOC106684534 isoform X2	4.7	59.3
253	TRINITY_DN36580_c0_g1_i1	No hit	0.8	59.3
254	TRINITY_DN22697_c0_g1_i1	No hit	1.4	59.2
255	TRINITY_DN2868_c0_g1_i2	Elongation of very long chain fatty acids protein 6	8.6	59.2
256	TRINITY_DN2889_c0_g1_i9	No hit	1.4	58.9
257	TRINITY_DN22163_c0_g1_i5	Uncharacterized protein LOC106681469	10.5	58.7
258	TRINITY_DN3701_c0_g1_i2	Sphingolipid delta(4)-desaturase DES1 isoform X1	12.8	58.4
259	TRINITY_DN24312_c1_g1_i1	No hit	0.6	58.1
260	TRINITY_DN50_c0_g1_i4	Uncharacterized protein LOC106680431	6.0	58.0
261	TRINITY_DN9772_c0_g1_i1	No hit	2.8	57.9
262	TRINITY_DN6300_c0_g1_i1	No hit	1.1	57.9
263	TRINITY_DN50178_c0_g1_i1	Sulfotransferase 4A1-like	2.2	57.2
264	TRINITY_DN5736_c0_g1_i2	Alpha-tocopherol transfer protein-like	2.2	57.2
265	TRINITY_DN53234_c0_g1_i1	No hit	0.0	56.9
266	TRINITY_DN9125_c0_g1_i3	2-acylglycerol O-acyltransferase 1-like	11.9	56.7
267	TRINITY_DN1289_c2_g1_i1	No hit	2.3	56.5
268	TRINITY_DN3018_c0_g3_i1	Trehalase	2.7	56.5
269	TRINITY_DN1227_c0_g1_i1	Serine/threonine-protein kinase tricorner isoform X1	5.6	55.6
270	TRINITY_DN12783_c0_g1_i4	No hit	1.2	55.6
271	TRINITY_DN15322_c0_g1_i1	Cathepsin L1 isoform X1	1.2	55.5
272	TRINITY_DN39347_c0_g1_i1	Uncharacterized protein LOC106678401	1.6	55.4
273	TRINITY_DN47660_c0_g1_i1	Uncharacterized protein LOC106683416	1.9	55.2
274	TRINITY_DN51030_c0_g1_i1	No hit	1.0	54.9
275	TRINITY_DN5291_c0_g1_i3	Probable cation-transporting ATPase 13A3	10.7	54.7
276	TRINITY_DN19249_c0_g1_i1	Legumain-like	3.5	54.4
277	TRINITY_DN16062_c0_g1_i3	Long-chain fatty acid transport protein 4	7.8	54.4
278	TRINITY_DN54966_c0_g1_i1	Lysosomal acid phosphatase-like	0.8	54.4
279	TRINITY_DN3140_c0_g1_i1	Very-long-chain 3-oxoacyl-CoA reductase-B-like	9.3	53.9
280	TRINITY_DN9078_c0_g1_i3	No hit	1.3	53.8
281	TRINITY_DN23049_c0_g1_i1	No hit	4.6	53.6
282	TRINITY_DN8946_c0_g1_i1	No hit	0.9	53.6
283	TRINITY_DN8792_c0_g1_i4	No hit	1.5	53.5
284	TRINITY_DN5708_c1_g1_i1	Alpha/beta hydrolase domain-containing protein 17B	11.7	53.2
285	TRINITY_DN50471_c0_g1_i1	Cathepsin D	0.6	52.8
286	TRINITY_DN3347_c0_g1_i1	Uncharacterized protein LOC112210696	2.2	52.0
287	TRINITY_DN5569_c0_g1_i1	No hit	2.4	51.5
288	TRINITY_DN9923_c0_g1_i1	No hit	0.0	50.8
289	TRINITY_DN10214_c0_g1_i2	No hit	1.1	50.7
290	TRINITY_DN1236_c0_g1_i12	No hit	1.0	50.5
291	TRINITY_DN18724_c0_g1_i1	No hit	0.7	50.1
292	TRINITY_DN18602_c0_g1_i1	No hit	4.7	49.9
293	TRINITY_DN11871_c0_g1_i10	No hit	0.6	49.5
294	TRINITY_DN38566_c0_g1_i1	UDP-glucuronosyltransferase 2B17-like	1.8	48.8
295	TRINITY_DN6535_c0_g1_i1	No hit	1.2	48.7
296	TRINITY_DN3783_c0_g1_i1	15-hydroxyprostaglandin dehydrogenase [NAD(+)]	3.0	48.4
297	TRINITY_DN25252_c0_g2_i2	Uncharacterized protein LOC112210943	3.4	48.4
298	TRINITY_DN5160_c0_g1_i2	No hit	1.3	48.2
299	TRINITY_DN7230_c0_g1_i39	No hit	4.0	47.8
300	TRINITY_DN1134_c0_g1_i4	Androgen-dependent TFPI-regulating protein-like	5.5	46.9
301	TRINITY_DN3555_c0_g1_i2	N(4)-(Beta-N-acetylglucosaminyl)-L-asparaginase-like	7.5	46.8
302	TRINITY_DN23385_c0_g1_i1	No hit	2.2	46.6
303	TRINITY_DN1431_c0_g2_i18	Tropomodulin-1 isoform X8	7.3	45.6
304	TRINITY_DN23981_c0_g1_i1	No hit	0.9	45.5

305	TRINITY_DN2753_c0_g1_i14	No hit	3.1	45.5
306	TRINITY_DN786_c3_g1_i2	Cathepsin L1 isoform X1	3.9	45.2
307	TRINITY_DN2138_c27_g1_i1	No hit	1.5	44.8
308	TRINITY_DN4023_c0_g1_i1	No hit	0.0	44.7
309	TRINITY_DN1236_c0_g1_i13	No hit	2.6	44.3
310	TRINITY_DN613_c0_g1_i2	Protein ref(2)P-like	6.8	44.2
311	TRINITY_DN58_c0_g1_i13	No hit	5.7	44.0
312	TRINITY_DN16547_c0_g1_i1	Protein croquemort-like isoform X1	1.3	43.6
313	TRINITY_DN22986_c0_g1_i1	No hit	0.5	43.4
314	TRINITY_DN1611_c0_g1_i3	CTL-like protein 2 isoform X2	5.7	43.4
315	TRINITY_DN441_c1_g2_i6	No hit	0.4	43.2
316	TRINITY_DN37273_c0_g1_i1	No hit	0.0	43.0
317	TRINITY_DN33421_c0_g1_i1	Protein CREG1-like	4.3	43.0
318	TRINITY_DN14859_c0_g3_i1	No hit	2.4	42.9
319	TRINITY_DN267_c0_g1_i71	No hit	2.1	42.7
320	TRINITY_DN3330_c0_g1_i3	Trypsin-1-like	1.5	42.5
321	TRINITY_DN37813_c0_g1_i1	No hit	0.3	41.9
322	TRINITY_DN53615_c0_g1_i1	Uncharacterized protein LOC106685890	1.0	41.7
323	TRINITY_DN3716_c0_g1_i1	No hit	0.6	41.6
324	TRINITY_DN2691_c0_g1_i4	Uncharacterized protein LOC106679890	5.7	41.5
325	TRINITY_DN43075_c0_g1_i1	Uncharacterized protein LOC106691924	1.0	41.4
326	TRINITY_DN30800_c0_g1_i1	No hit	0.5	40.6
327	TRINITY_DN1793_c1_g1_i1	No hit	5.4	40.0
328	TRINITY_DN8017_c0_g2_i1	ATP-binding cassette sub-family A member 3-like isoform X1	5.1	39.8
329	TRINITY_DN2753_c0_g1_i5	RCC1 and BTB domain-containing protein 1-like isoform X2	7.9	39.4
330	TRINITY_DN1654_c0_g1_i3	Endochitinase At2g43620	2.7	39.2
331	TRINITY_DN885_c0_g1_i2	Transcriptional protein SWT1 isoform X1	1.5	39.0
332	TRINITY_DN41067_c0_g1_i1	No hit	0.6	38.7
333	TRINITY_DN33871_c0_g1_i1	No hit	0.9	38.7
334	TRINITY_DN1903_c1_g1_i1	Uncharacterized protein LOC106684378 isoform X2	2.0	38.6
335	TRINITY_DN5621_c1_g2_i1	No hit	4.1	38.5
336	TRINITY_DN1793_c1_g1_i5	No hit	3.4	38.3
337	TRINITY_DN991_c0_g1_i2	No hit	2.3	38.3
338	TRINITY_DN14760_c0_g1_i1	No hit	4.7	38.1
339	TRINITY_DN16628_c0_g1_i1	No hit	1.4	38.1
340	TRINITY_DN1755_c0_g1_i1	Proton-coupled amino acid transporter 1-like	7.2	38.1
341	TRINITY_DN9812_c1_g2_i1	No hit	1.7	38.1
342	TRINITY_DN4990_c0_g1_i1	Mitochondrial 2-oxoglutarate/malate carrier protein-like	7.9	38.0
343	TRINITY_DN1761_c0_g1_i18	No hit	0.9	37.6
344	TRINITY_DN4219_c0_g1_i1	ETS homologous factor	4.2	37.6
345	TRINITY_DN9826_c0_g1_i4	Palmitoyl-protein thioesterase 1	5.6	37.6
346	TRINITY_DN612_c1_g1_i1	Monocarboxylate transporter 13	6.0	37.5
347	TRINITY_DN2206_c0_g1_i41	No hit	5.9	37.0
348	TRINITY_DN1493_c0_g1_i1	No hit	1.9	37.0
349	TRINITY_DN27382_c0_g1_i1	No hit	2.5	36.9
350	TRINITY_DN25137_c0_g1_i1	Hydroxyacyl-coenzyme A dehydrogenase, mitochondrial-like	3.0	36.9
351	TRINITY_DN18837_c0_g1_i1	UDP-glucuronosyltransferase 2B17-like	2.9	36.9
352	TRINITY_DN49162_c0_g1_i1	No hit	3.2	36.4
353	TRINITY_DN10952_c0_g1_i2	No hit	5.4	36.2
354	TRINITY_DN39093_c0_g1_i1	Uncharacterized protein LOC106685890	1.4	36.2
355	TRINITY_DN2556_c0_g1_i2	Reticulon-4-like isoform X1	6.4	36.1
356	TRINITY_DN21274_c0_g1_i1	No hit	2.7	36.1
357	TRINITY_DN1500_c0_g1_i3	Integrin alpha-PS2 isoform X3	2.0	36.0
358	TRINITY_DN513_c0_g2_i2	Esterase FE4-like	1.1	35.9
359	TRINITY_DN1497_c0_g1_i3	No hit	3.3	35.8
360	TRINITY_DN1387_c0_g2_i1	Bumetanide-sensitive sodium-(potassium)-chloride cotransporter, partial	6.3	35.6
361	TRINITY_DN44233_c0_g1_i1	No hit	0.0	35.6
362	TRINITY_DN5150_c0_g1_i4	No hit	4.9	35.5
363	TRINITY_DN4891_c0_g1_i1	Esterase FE4-like	3.9	35.5
364	TRINITY_DN5577_c0_g1_i4	ETS-like protein pointed	3.2	35.5
365	TRINITY_DN1671_c0_g1_i7	Uncharacterized protein LOC106681588 isoform X1	3.9	35.3
366	TRINITY_DN58_c5_g1_i2	No hit	1.7	35.1
367	TRINITY_DN6261_c0_g1_i1	Uncharacterized protein LOC106692576	0.6	35.0
368	TRINITY_DN8108_c0_g1_i6	No hit	1.1	34.9
369	TRINITY_DN341_c0_g1_i6	No hit	1.5	34.9
370	TRINITY_DN54035_c0_g1_i1	Cathepsin L1-like	1.4	34.6
371	TRINITY_DN50950_c0_g1_i1	No hit	0.8	34.6
372	TRINITY_DN27205_c0_g1_i1	ATP-binding cassette sub-family G member 1 isoform X1	0.7	34.5
373	TRINITY_DN1387_c0_g1_i1	No hit	3.6	34.4
374	TRINITY_DN30165_c0_g1_i1	No hit	1.6	33.6
375	TRINITY_DN8792_c0_g1_i6	No hit	0.8	33.4
376	TRINITY_DN1832_c2_g1_i6	No hit	2.7	33.4
377	TRINITY_DN584_c0_g1_i2	No hit	6.4	33.4
378	TRINITY_DN148_c1_g1_i1	No hit	2.1	33.4
379	TRINITY_DN13205_c1_g1_i1	No hit	1.6	33.2
380	TRINITY_DN1776_c2_g1_i2	No hit	2.2	33.2
381	TRINITY_DN4572_c0_g1_i1	No hit	0.6	33.1

382	TRINITY_DN3020_c0_g1_i4	No hit	2.1	33.0
383	TRINITY_DN52781_c0_g1_i1	No hit	1.9	33.0
384	TRINITY_DN15728_c0_g1_i1	No hit	0.1	32.9
385	TRINITY_DN32874_c0_g1_i1	Uncharacterized protein LOC106682399	4.6	32.9
386	TRINITY_DN15489_c0_g1_i1	No hit	1.0	32.8
387	TRINITY_DN10373_c1_g1_i1	No hit	0.6	32.5
388	TRINITY_DN38928_c0_g1_i1	Uncharacterized protein LOC106689064	0.9	32.4
389	TRINITY_DN33965_c0_g1_i1	Venom carboxylesterase-6-like	1.0	32.3
390	TRINITY_DN1431_c0_g2_i9	Tropomodulin-1 isoform X5	0.6	32.3
391	TRINITY_DN2556_c0_g1_i4	Reticulon-1-like isoform X2	3.4	32.2
392	TRINITY_DN27740_c0_g1_i1	No hit	1.9	32.2
393	TRINITY_DN51091_c0_g1_i1	No hit	1.3	32.1
394	TRINITY_DN3131_c0_g2_i1	No hit	2.5	32.1
395	TRINITY_DN13611_c0_g1_i1	No hit	0.0	31.8
396	TRINITY_DN1129_c0_g1_i1	Uncharacterized protein LOC106679633	6.6	31.7
397	TRINITY_DN473_c0_g1_i4	Elongation of very long chain fatty acids protein AAEL008004 isoform X1	5.1	31.5
398	TRINITY_DN13136_c0_g2_i1	No hit	1.3	31.4
399	TRINITY_DN7765_c0_g1_i1	No hit	0.7	31.1
400	TRINITY_DN23096_c0_g1_i1	Uncharacterized protein LOC106681858	3.1	30.8
401	TRINITY_DN39231_c0_g1_i1	No hit	0.0	30.7
402	TRINITY_DN1113_c0_g1_i1	No hit	4.0	30.6
403	TRINITY_DN8184_c0_g1_i5	No hit	0.8	30.4
404	TRINITY_DN17858_c0_g1_i1	No hit	1.1	30.2
405	TRINITY_DN10410_c0_g1_i1	No hit	1.4	30.2
406	TRINITY_DN36615_c0_g1_i1	No hit	1.9	30.2
407	TRINITY_DN33695_c0_g1_i1	No hit	0.6	29.9
408	TRINITY_DN5297_c0_g1_i1	No hit	1.3	29.9
409	TRINITY_DN3084_c0_g1_i1	No hit	0.4	29.9
410	TRINITY_DN58_c1_g1_i2	No hit	1.9	29.8
411	TRINITY_DN8304_c0_g1_i1	No hit	0.7	29.7
412	TRINITY_DN30614_c0_g1_i2	5-hydroxyisourate hydrolase	0.6	29.7
413	TRINITY_DN43218_c0_g1_i1	No hit	0.5	29.7
414	TRINITY_DN2329_c0_g1_i12	Procardosin-A	0.6	29.7
415	TRINITY_DN53732_c0_g1_i1	No hit	0.6	29.6
416	TRINITY_DN53746_c0_g1_i1	No hit	0.9	29.6
417	TRINITY_DN7840_c0_g1_i1	No hit	1.6	29.5
418	TRINITY_DN48208_c0_g1_i1	No hit	0.0	29.4
419	TRINITY_DN15939_c0_g1_i1	No hit	2.5	29.3
420	TRINITY_DN11243_c1_g2_i1	UDP-glucuronosyltransferase 2C1	1.4	29.2
421	TRINITY_DN1500_c0_g1_i2	Integrin alpha-PS2 isoform X2	2.4	28.9
422	TRINITY_DN14859_c0_g1_i2	Uncharacterized protein LOC106683312	0.1	28.8
423	TRINITY_DN1201_c1_g1_i3	No hit	4.4	28.8
424	TRINITY_DN52335_c0_g1_i1	No hit	1.6	28.8
425	TRINITY_DN32400_c0_g1_i1	Cathepsin L1-like	0.7	28.7
426	TRINITY_DN4047_c3_g1_i1	No hit	0.2	28.7
427	TRINITY_DN12096_c0_g1_i6	Uncharacterized protein LOC106684378 isoform X2	1.1	28.6
428	TRINITY_DN44666_c0_g1_i1	No hit	2.1	28.6
429	TRINITY_DN22939_c0_g1_i1	Protein giant-lens	1.3	28.5
430	TRINITY_DN1138_c0_g1_i3	UNC93-like protein isoform X1	4.0	28.5
431	TRINITY_DN2992_c0_g1_i7	No hit	1.1	28.4
432	TRINITY_DN12314_c2_g1_i2	Titin isoform X2	2.0	28.4
433	TRINITY_DN1776_c2_g1_i1	No hit	1.8	28.3
434	TRINITY_DN3843_c0_g1_i2	No hit	1.6	28.3
435	TRINITY_DN20400_c0_g1_i1	No hit	0.0	28.3
436	TRINITY_DN30267_c0_g1_i1	No hit	0.0	28.2
437	TRINITY_DN3604_c1_g1_i1	No hit	1.4	28.0
438	TRINITY_DN5839_c4_g1_i1	No hit	0.4	28.0
439	TRINITY_DN24682_c0_g1_i1	No hit	2.6	28.0
440	TRINITY_DN30432_c0_g1_i1	No hit	1.8	27.9
441	TRINITY_DN10952_c0_g1_i5	No hit	0.7	27.9
442	TRINITY_DN26344_c0_g1_i1	No hit	4.8	27.8
443	TRINITY_DN9310_c0_g1_i1	No hit	1.2	27.8
444	TRINITY_DN35037_c0_g1_i1	Uncharacterized protein LOC106678734	4.1	27.6
445	TRINITY_DN10968_c0_g1_i1	No hit	2.5	27.5
446	TRINITY_DN1430_c1_g1_i2	No hit	4.9	27.4
447	TRINITY_DN194_c0_g1_i61	Lipase member N-like	3.6	27.3
448	TRINITY_DN37204_c0_g1_i1	No hit	1.3	27.3
449	TRINITY_DN1569_c0_g1_i6	Cytochrome b561	3.1	27.3
450	TRINITY_DN4586_c0_g1_i1	5'-AMP-activated protein kinase subunit beta-1 isoform X2	3.7	27.2
451	TRINITY_DN19249_c0_g2_i1	Legumain-like	1.4	27.1
452	TRINITY_DN268_c0_g1_i1	Uncharacterized protein LOC106688406 isoform X1	4.1	27.0
453	TRINITY_DN23096_c0_g1_i2	Uncharacterized protein LOC106681858	1.5	27.0
454	TRINITY_DN52220_c0_g1_i1	No hit	1.8	26.9
455	TRINITY_DN12863_c0_g1_i1	Acylphosphatase-2	0.8	26.8
456	TRINITY_DN7106_c2_g1_i4	Probable cytochrome P450 6a13	0.0	26.8
457	TRINITY_DN39051_c0_g1_i1	Venom serine carboxypeptidase	0.8	26.8
458	TRINITY_DN14265_c0_g2_i1	Uncharacterized protein LOC106680707	1.6	26.7

459	TRINITY_DN11152_c0_g1_i2	No hit	0.5	26.7
460	TRINITY_DN18596_c0_g1_i1	No hit	0.5	26.6
461	TRINITY_DN17867_c0_g1_i1	Titin-like	2.7	26.3
462	TRINITY_DN17115_c3_g1_i1	No hit	0.0	26.3
463	TRINITY_DN7752_c3_g1_i1	Uncharacterized protein LOC106686593 isoform X1	2.6	26.3
464	TRINITY_DN32653_c0_g1_i1	No hit	1.7	26.2
465	TRINITY_DN46651_c0_g1_i1	Uncharacterized protein LOC106689355	0.7	26.1
466	TRINITY_DN1776_c5_g1_i1	No hit	0.7	26.1
467	TRINITY_DN43372_c0_g1_i1	No hit	0.4	26.0
468	TRINITY_DN513_c0_g1_i6	Esterase FE4-like	4.3	25.9
469	TRINITY_DN3591_c0_g1_i1	Uncharacterized protein LOC106687111 isoform X1	2.3	25.9
470	TRINITY_DN1500_c0_g2_i1	Integrin alpha-PS2 isoform X3	2.4	25.9
471	TRINITY_DN1081_c4_g1_i1	No hit	1.5	25.8
472	TRINITY_DN609_c1_g1_i1	No hit	0.5	25.8
473	TRINITY_DN7589_c0_g1_i4	Hexokinase type 2 isoform X2	1.5	25.7
474	TRINITY_DN997_c5_g1_i1	No hit	1.3	25.7
475	TRINITY_DN3030_c0_g1_i1	Aminopeptidase M1	1.0	25.6
476	TRINITY_DN46025_c0_g1_i1	Uncharacterized protein LOC106683703	0.3	25.5
477	TRINITY_DN25542_c0_g1_i1	No hit	0.5	25.4
478	TRINITY_DN6502_c0_g1_i1	Pancreatic lipase-related protein 2-like	0.6	25.4
479	TRINITY_DN58_c1_g1_i1	No hit	0.9	25.1
480	TRINITY_DN20400_c0_g2_i1	No hit	1.0	24.9
481	TRINITY_DN5338_c0_g2_i1	Venom carboxylesterase-6-like	0.9	24.9
482	TRINITY_DN40145_c0_g1_i1	No hit	0.7	24.9
483	TRINITY_DN17254_c0_g1_i1	No hit	1.1	24.9
484	TRINITY_DN50626_c0_g1_i1	Alkaline phosphatase	2.2	24.7
485	TRINITY_DN381_c0_g1_i1	No hit	3.2	24.7
486	TRINITY_DN14859_c0_g1_i1	Uncharacterized protein LOC106683312	0.9	24.7
487	TRINITY_DN14186_c0_g1_i1	No hit	0.6	24.6
488	TRINITY_DN640_c0_g1_i2	No hit	1.6	24.5
489	TRINITY_DN11562_c0_g1_i4	No hit	0.5	24.3
490	TRINITY_DN4540_c0_g1_i2	Uncharacterized protein LOC106679890	4.4	24.3
491	TRINITY_DN38010_c0_g1_i1	No hit	1.7	24.2
492	TRINITY_DN55594_c0_g1_i1	Alpha-tocopherol transfer protein-like	2.6	24.2
493	TRINITY_DN10968_c0_g3_i1	No hit	1.8	24.0
494	TRINITY_DN381_c0_g2_i1	No hit	4.2	23.9
495	TRINITY_DN2515_c1_g1_i1	No hit	1.5	23.8
496	TRINITY_DN38562_c0_g1_i1	No hit	2.9	23.7
497	TRINITY_DN17044_c0_g1_i1	Facilitated trehalose transporter Tret1-like isoform X2	2.7	23.7
498	TRINITY_DN16215_c0_g2_i1	No hit	1.4	23.7
499	TRINITY_DN27190_c0_g1_i1	Voltage-dependent calcium channel type A subunit alpha-1	0.5	23.6
500	TRINITY_DN3020_c0_g2_i1	Krueppel-like factor 3	2.0	23.6
501	TRINITY_DN35389_c0_g1_i1	No hit	0.8	23.5
502	TRINITY_DN15746_c0_g1_i1	No hit	0.6	23.5
503	TRINITY_DN6722_c0_g1_i9	Protein UBASH3A homolog	3.7	23.4
504	TRINITY_DN27227_c0_g1_i1	Monocarboxylate transporter 14 isoform X2	0.8	23.4
505	TRINITY_DN25502_c1_g1_i2	No hit	0.3	23.3
506	TRINITY_DN19003_c0_g2_i1	No hit	1.3	23.1
507	TRINITY_DN14862_c0_g1_i1	Myrosinase 1	4.3	23.1
508	TRINITY_DN1071_c0_g1_i12	Nesprin-1	5.0	23.1
509	TRINITY_DN695_c0_g2_i4	Transient receptor potential cation channel subfamily A member 1	4.9	22.9
510	TRINITY_DN212_c0_g1_i7	Secretory carrier-associated membrane protein 1 isoform X3	4.9	22.7
511	TRINITY_DN55538_c0_g1_i1	No hit	1.0	22.5
512	TRINITY_DN30583_c0_g1_i1	No hit	0.7	22.5
513	TRINITY_DN37566_c0_g1_i1	No hit	0.3	22.4
514	TRINITY_DN513_c0_g1_i3	Esterase FE4-like	0.9	22.3
515	TRINITY_DN51931_c0_g1_i1	No hit	0.8	22.3
516	TRINITY_DN28402_c0_g1_i1	No hit	1.3	22.2
517	TRINITY_DN31752_c0_g3_i1	Kinesin-like protein KIF12	0.1	22.2
518	TRINITY_DN13570_c0_g1_i1	Uncharacterized protein LOC106684371	0.8	22.2
519	TRINITY_DN8538_c1_g2_i1	No hit	0.4	22.1
520	TRINITY_DN10554_c0_g2_i1	No hit	0.2	22.1
521	TRINITY_DN8120_c0_g1_i2	DNA damage-regulated autophagy modulator protein 2-like	3.3	22.1
522	TRINITY_DN11086_c0_g1_i1	No hit	0.9	21.9
523	TRINITY_DN2717_c0_g1_i1	Facilitated trehalose transporter Tret1	1.5	21.8
524	TRINITY_DN1362_c0_g1_i12	TLD domain-containing protein 2-like isoform X2	0.3	21.8
525	TRINITY_DN40835_c0_g1_i1	Flightin isoform X2	0.5	21.8
526	TRINITY_DN26236_c0_g1_i1	No hit	0.7	21.7
527	TRINITY_DN40696_c0_g1_i1	No hit	0.4	21.5
528	TRINITY_DN50015_c0_g1_i1	No hit	0.4	21.4
529	TRINITY_DN2908_c3_g1_i3	Probable cytochrome P450 6d5 isoform X1	4.2	21.2
530	TRINITY_DN54021_c0_g1_i1	No hit	0.2	21.1
531	TRINITY_DN10952_c0_g1_i1	No hit	1.9	21.1
532	TRINITY_DN243_c0_g1_i97	Uncharacterized protein LOC106687821 isoform X12	0.0	21.0
533	TRINITY_DN5785_c0_g1_i5	SEC14-like protein 2 isoform X1	0.5	20.9
534	TRINITY_DN4285_c1_g1_i20	No hit	2.0	20.9
535	TRINITY_DN2602_c0_g1_i1	Uncharacterized protein LOC106682564	1.8	20.9

536	TRINITY_DN57117_c0_g1_i1	No hit	0.0	20.8
537	TRINITY_DN24312_c0_g1_i1	Aggrecan core protein-like	1.0	20.7
538	TRINITY_DN17677_c0_g2_i1	Titin isoform X3	0.9	20.7
539	TRINITY_DN43021_c0_g1_i1	No hit	0.2	20.7
540	TRINITY_DN3080_c0_g1_i3	Phosphoglycerate kinase isoform X2	3.3	20.7
541	TRINITY_DN5577_c0_g1_i2	ETS-like protein pointed	1.4	20.6
542	TRINITY_DN14123_c1_g1_i1	No hit	3.0	20.6
543	TRINITY_DN45155_c0_g1_i1	No hit	0.4	20.6
544	TRINITY_DN25502_c1_g1_i1	No hit	0.9	20.6
545	TRINITY_DN30800_c0_g2_i1	No hit	1.0	20.5
546	TRINITY_DN39153_c0_g1_i1	No hit	1.0	20.5
547	TRINITY_DN7046_c0_g1_i5	Inward rectifier potassium channel 2-like isoform X1	1.9	20.5
548	TRINITY_DN3736_c0_g3_i2	Thymosin beta isoform X4	3.2	20.4
549	TRINITY_DN3131_c0_g1_i1	Ets DNA-binding protein pokkuri	2.1	20.4
550	TRINITY_DN18969_c0_g1_i1	No hit	2.1	20.4
551	TRINITY_DN41536_c0_g1_i1	No hit	0.4	20.3
552	TRINITY_DN1793_c1_g1_i7	No hit	3.1	20.2
553	TRINITY_DN358_c0_g1_i3	SH2 domain-containing protein 4B isoform X1	1.6	20.2
554	TRINITY_DN243_c0_g2_i1	No hit	0.5	20.1
555	TRINITY_DN13109_c0_g1_i2	Aromatic-L-amino-acid decarboxylase	2.3	20.1
556	TRINITY_DN3623_c0_g1_i2	No hit	0.5	20.1
557	TRINITY_DN863_c0_g2_i2	No hit	0.3	20.1
558	TRINITY_DN194_c2_g1_i1	No hit	3.2	20.1
559	TRINITY_DN8634_c0_g2_i1	Sodium/potassium/calcium exchanger 4-like isoform X1	1.8	20.0
560	TRINITY_DN16394_c0_g1_i1	Uncharacterized protein LOC106684989	1.2	19.9
561	TRINITY_DN10688_c0_g1_i1	No hit	0.5	19.9
562	TRINITY_DN4312_c0_g1_i12	No hit	0.7	19.8
563	TRINITY_DN3583_c0_g1_i11	No hit	0.9	19.8
564	TRINITY_DN39603_c0_g1_i1	Protein big brother isoform X2	2.6	19.7
565	TRINITY_DN18226_c0_g1_i2	WSC domain-containing protein ARB 07867-like	0.4	19.6
566	TRINITY_DN10671_c0_g1_i1	No hit	0.7	19.6
567	TRINITY_DN50_c0_g1_i20	Uncharacterized protein LOC106680431	2.5	19.5
568	TRINITY_DN54494_c0_g1_i1	No hit	0.5	19.5
569	TRINITY_DN23170_c0_g2_i1	Titin isoform X5	0.4	19.5
570	TRINITY_DN36502_c0_g1_i1	No hit	0.0	19.5
571	TRINITY_DN37710_c0_g1_i1	No hit	0.3	19.4
572	TRINITY_DN1793_c1_g2_i1	No hit	1.1	19.3
573	TRINITY_DN18066_c0_g1_i1	Uncharacterized protein LOC106681360	2.6	19.2
574	TRINITY_DN52081_c0_g1_i1	No hit	1.1	19.2
575	TRINITY_DN2889_c0_g1_i12	No hit	1.3	19.0
576	TRINITY_DN10373_c1_g2_i1	No hit	0.6	19.0
577	TRINITY_DN5163_c0_g1_i1	No hit	0.3	18.9
578	TRINITY_DN9109_c0_g1_i1	Uncharacterized protein LOC106689212	0.5	18.9
579	TRINITY_DN14573_c0_g1_i2	No hit	0.4	18.9
580	TRINITY_DN10227_c0_g1_i1	No hit	0.5	18.8
581	TRINITY_DN2144_c0_g1_i3	Phospholipid-transporting ATPase ID, partial	0.8	18.8
582	TRINITY_DN16062_c0_g1_i2	No hit	0.9	18.8
583	TRINITY_DN51114_c0_g1_i1	No hit	1.5	18.8
584	TRINITY_DN40955_c0_g1_i1	No hit	0.6	18.8
585	TRINITY_DN56461_c0_g1_i1	No hit	0.7	18.7
586	TRINITY_DN8069_c0_g1_i1	Discoidin domain-containing receptor 2 isoform X1	1.2	18.7
587	TRINITY_DN31431_c0_g1_i1	Adenylate cyclase type 8-like isoform X1	1.0	18.7
588	TRINITY_DN42729_c0_g1_i1	No hit	0.5	18.6
589	TRINITY_DN4224_c0_g1_i6	No hit	0.7	18.6
590	TRINITY_DN34942_c0_g1_i1	Uncharacterized protein LOC106691513	0.6	18.6
591	TRINITY_DN12158_c0_g1_i33	Acetyl-CoA carboxylase 1	0.2	18.6
592	TRINITY_DN2088_c0_g1_i12	Copper-transporting ATPase 1 isoform X2	0.3	18.6
593	TRINITY_DN53640_c0_g1_i1	Zinc finger protein Elbow	1.8	18.6
594	TRINITY_DN1771_c0_g1_i3	Adventurous-gliding motility protein Z-like isoform X2	4.0	18.5
595	TRINITY_DN6309_c0_g1_i2	Protein peste-like	0.8	18.5
596	TRINITY_DN3260_c1_g1_i1	No hit	0.4	18.5
597	TRINITY_DN9396_c0_g1_i1	No hit	0.6	18.5
598	TRINITY_DN45318_c0_g1_i1	No hit	1.7	18.5
599	TRINITY_DN2113_c0_g1_i2	No hit	0.5	18.4
600	TRINITY_DN1730_c0_g1_i1	Glycerol kinase	1.0	18.4
601	TRINITY_DN5338_c0_g1_i1	No hit	1.3	18.2
602	TRINITY_DN7124_c0_g1_i1	Protein lethal(2)essential for life-like isoform X1	2.8	18.2
603	TRINITY_DN2262_c0_g1_i6	No hit	0.0	18.1
604	TRINITY_DN50486_c0_g1_i1	Prothoracicostatic peptide-like	0.7	18.1
605	TRINITY_DN2206_c0_g1_i21	Microtubule-actin cross-linking factor 1 isoform X4	4.3	18.0
606	TRINITY_DN15650_c0_g1_i1	Uncharacterized protein LOC106683911	0.3	18.0
607	TRINITY_DN16105_c0_g4_i1	No hit	1.5	17.8
608	TRINITY_DN5042_c0_g1_i1	Uncharacterized protein LOC106685553	0.8	17.8
609	TRINITY_DN26773_c0_g2_i1	No hit	1.1	17.8
610	TRINITY_DN56319_c0_g1_i1	No hit	1.3	17.8
611	TRINITY_DN4853_c0_g2_i4	No hit	1.3	17.7
612	TRINITY_DN1071_c0_g1_i11	Nesprin-1	3.0	17.7

613	TRINITY_DN58_c0_g1_i11	No hit	2.3	17.6
614	TRINITY_DN33107_c0_g1_i1	No hit	0.0	17.6
615	TRINITY_DN17677_c0_g1_i1	Titin isoform X3	0.7	17.6
616	TRINITY_DN15100_c1_g1_i1	Uncharacterized protein LOC106678018	0.6	17.5
617	TRINITY_DN28420_c0_g1_i1	No hit	1.1	17.5
618	TRINITY_DN4037_c1_g1_i1	No hit	0.1	17.5
619	TRINITY_DN33262_c0_g1_i1	No hit	0.0	17.5
620	TRINITY_DN24622_c0_g1_i1	No hit	0.2	17.5
621	TRINITY_DN38882_c0_g1_i1	Facilitated trehalose transporter Tret1-like	0.7	17.5
622	TRINITY_DN12365_c0_g1_i1	No hit	0.0	17.4
623	TRINITY_DN7630_c0_g1_i1	No hit	1.2	17.4
624	TRINITY_DN56395_c0_g1_i1	No hit	0.7	17.4
625	TRINITY_DN39150_c0_g1_i1	Inositol monophosphatase 2	1.4	17.2
626	TRINITY_DN2125_c0_g1_i2	Cathepsin L1-like isoform X3	0.3	17.2
627	TRINITY_DN14383_c0_g1_i1	NADP-dependent malic enzyme isoform X1	3.6	17.2
628	TRINITY_DN9979_c0_g1_i3	Calcium/calmodulin-dependent protein kinase type II alpha chain isoform X20	2.0	16.9
629	TRINITY_DN2208_c0_g1_i7	Uncharacterized protein LOC106690964	1.0	16.9
630	TRINITY_DN53822_c0_g1_i1	No hit	0.8	16.9
631	TRINITY_DN3753_c1_g1_i7	No hit	0.4	16.9
632	TRINITY_DN43173_c0_g1_i1	Uncharacterized protein LOC106687787	1.8	16.8
633	TRINITY_DN22804_c0_g1_i1	No hit	0.7	16.8
634	TRINITY_DN44698_c0_g1_i1	No hit	0.4	16.7
635	TRINITY_DN48544_c0_g1_i1	No hit	0.4	16.7
636	TRINITY_DN2613_c0_g1_i16	Serpin B4-like	1.9	16.7
637	TRINITY_DN50_c0_g1_i2	Uncharacterized protein LOC106680431	3.0	16.7
638	TRINITY_DN6656_c0_g1_i1	No hit	0.7	16.7
639	TRINITY_DN53197_c0_g1_i1	No hit	0.7	16.7
640	TRINITY_DN10968_c0_g2_i1	No hit	1.9	16.6
641	TRINITY_DN14623_c0_g1_i1	GILT-like protein 1	1.6	16.6
642	TRINITY_DN24182_c0_g1_i1	No hit	0.6	16.6
643	TRINITY_DN2594_c0_g2_i1	No hit	1.0	16.6
644	TRINITY_DN38390_c0_g1_i1	No hit	0.7	16.6
645	TRINITY_DN36453_c0_g1_i1	No hit	0.7	16.6
646	TRINITY_DN53682_c0_g1_i1	No hit	0.3	16.6
647	TRINITY_DN8163_c0_g1_i5	Homeobox protein homothorax isoform X1	2.3	16.5
648	TRINITY_DN58_c2_g1_i2	No hit	2.6	16.5
649	TRINITY_DN317_c2_g1_i1	No hit	0.3	16.5
650	TRINITY_DN6818_c0_g2_i1	No hit	1.4	16.5
651	TRINITY_DN50757_c0_g1_i1	GAS2-like protein pickled eggs	1.9	16.4
652	TRINITY_DN5646_c1_g1_i1	No hit	0.0	16.3
653	TRINITY_DN22266_c0_g2_i1	No hit	1.3	16.3
654	TRINITY_DN32232_c0_g1_i1	No hit	0.5	16.3
655	TRINITY_DN4124_c0_g1_i3	No hit	1.6	16.2
656	TRINITY_DN44239_c0_g1_i1	No hit	0.0	16.1
657	TRINITY_DN27205_c0_g2_i1	ATP-binding cassette sub-family G member 4 isoform X2	0.3	16.0
658	TRINITY_DN48383_c0_g1_i1	Lipase 1-like isoform X2	1.1	16.0
659	TRINITY_DN15162_c0_g1_i5	Peroxidasin homolog	0.8	16.0
660	TRINITY_DN5614_c0_g2_i1	No hit	1.1	16.0
661	TRINITY_DN30888_c0_g1_i1	No hit	0.4	15.9
662	TRINITY_DN42548_c0_g1_i1	No hit	0.8	15.9
663	TRINITY_DN10627_c0_g1_i2	Tryptase-like	1.6	15.8
664	TRINITY_DN1263_c4_g1_i1	No hit	0.5	15.7
665	TRINITY_DN23170_c0_g1_i1	Titin isoform X5	0.6	15.7
666	TRINITY_DN663_c0_g1_i1	No hit	1.6	15.7
667	TRINITY_DN20089_c0_g1_i1	Solute carrier organic anion transporter family member 5A1	0.9	15.7
668	TRINITY_DN1131_c3_g1_i1	No hit	0.4	15.6
669	TRINITY_DN37243_c0_g1_i1	No hit	0.6	15.6
670	TRINITY_DN39452_c0_g1_i1	No hit	0.0	15.5
671	TRINITY_DN2757_c0_g1_i3	No hit	1.4	15.5
672	TRINITY_DN8973_c0_g1_i1	No hit	0.9	15.4
673	TRINITY_DN28728_c0_g1_i1	No hit	1.2	15.3
674	TRINITY_DN14573_c0_g3_i1	Cathepsin B-like	0.4	15.3
675	TRINITY_DN4215_c0_g1_i1	Uncharacterized protein LOC106685898	0.4	15.3
676	TRINITY_DN18332_c0_g1_i3	Axin isoform X6	2.6	15.3
677	TRINITY_DN325_c0_g1_i3	Gastricsin-like	1.4	15.3
678	TRINITY_DN57146_c0_g1_i1	No hit	0.3	15.3
679	TRINITY_DN1906_c0_g1_i3	Transient receptor potential channel pyrexia-like	0.0	15.3
680	TRINITY_DN580_c1_g1_i1	No hit	0.9	15.3
681	TRINITY_DN31982_c0_g1_i1	No hit	0.0	15.2
682	TRINITY_DN5807_c0_g1_i1	No hit	0.3	15.2
683	TRINITY_DN14933_c0_g1_i1	No hit	0.1	15.2
684	TRINITY_DN47557_c0_g1_i1	No hit	1.6	15.2
685	TRINITY_DN12438_c0_g1_i1	No hit	0.9	15.2
686	TRINITY_DN48646_c0_g1_i1	No hit	0.2	15.2
687	TRINITY_DN5058_c0_g1_i1	No hit	0.9	15.2
688	TRINITY_DN5025_c0_g1_i1	No hit	0.9	15.1
689	TRINITY_DN34252_c0_g2_i1	No hit	0.5	15.1

690	TRINITY_DN12448_c0_g1_i1	No hit	1.7	15.1
691	TRINITY_DN15162_c0_g1_i10	Peroxidasin homolog	1.2	15.1
692	TRINITY_DN17146_c0_g1_i1	No hit	0.3	15.0
693	TRINITY_DN2834_c0_g3_i1	No hit	2.1	15.0
694	TRINITY_DN3589_c1_g1_i1	No hit	0.6	15.0
695	TRINITY_DN2329_c0_g2_i1	No hit	0.4	15.0
696	TRINITY_DN2730_c0_g1_i1	No hit	0.6	14.9
697	TRINITY_DN13013_c0_g1_i1	No hit	1.2	14.9
698	TRINITY_DN8244_c1_g1_i2	No hit	1.1	14.9
699	TRINITY_DN51686_c0_g1_i1	No hit	0.7	14.8
700	TRINITY_DN9979_c0_g3_i1	No hit	2.0	14.8
701	TRINITY_DN30273_c0_g1_i1	No hit	0.2	14.7
702	TRINITY_DN1771_c0_g1_i4	Protein MLP1 homolog isoform X4	2.1	14.7
703	TRINITY_DN33859_c0_g1_i1	No hit	0.3	14.6
704	TRINITY_DN4945_c0_g1_i7	Coronin-2B isoform X2	2.2	14.6
705	TRINITY_DN40059_c0_g1_i1	No hit	0.2	14.6
706	TRINITY_DN341_c0_g1_i7	No hit	1.4	14.6
707	TRINITY_DN27121_c1_g1_i1	No hit	2.8	14.6
708	TRINITY_DN243_c0_g1_i8	Uncharacterized protein LOC106687821 isoform X12	0.1	14.6
709	TRINITY_DN6093_c0_g1_i4	Ephrin type-B receptor 1-B	0.8	14.6
710	TRINITY_DN2300_c0_g1_i1	Uncharacterized protein LOC106677170, partial	0.5	14.6
711	TRINITY_DN1695_c0_g4_i1	No hit	1.1	14.6
712	TRINITY_DN32478_c0_g1_i1	No hit	0.8	14.5
713	TRINITY_DN27204_c0_g1_i1	No hit	2.1	14.5
714	TRINITY_DN11787_c0_g1_i1	No hit	0.1	14.5
715	TRINITY_DN48340_c0_g1_i1	No hit	0.0	14.5
716	TRINITY_DN5537_c0_g1_i9	No hit	0.7	14.5
717	TRINITY_DN105_c0_g1_i10	Serine/threonine-protein phosphatase 6 regulatory ankyrin repeat subunit A-like	0.6	14.4
718	TRINITY_DN21375_c0_g1_i1	No hit	0.4	14.4
719	TRINITY_DN9546_c0_g1_i1	No hit	0.4	14.4
720	TRINITY_DN13460_c0_g3_i1	Uncharacterized protein LOC106683213	0.9	14.4
721	TRINITY_DN26470_c0_g1_i1	Uncharacterized protein LOC106686162, partial	0.7	14.4
722	TRINITY_DN55943_c1_g1_i1	No hit	1.1	14.3
723	TRINITY_DN2613_c0_g2_i1	No hit	0.3	14.3
724	TRINITY_DN33717_c0_g1_i1	No hit	0.7	14.2
725	TRINITY_DN47216_c0_g1_i1	No hit	0.6	14.2
726	TRINITY_DN8163_c0_g1_i2	Homeobox protein homothorax isoform X1	1.6	14.2
727	TRINITY_DN38711_c0_g1_i1	Uncharacterized protein LOC106678591	0.6	14.1
728	TRINITY_DN35278_c0_g1_i1	Uncharacterized protein LOC106678591	0.5	14.1
729	TRINITY_DN3658_c0_g1_i36	Trypsin-2-like isoform X1	3.0	14.1
730	TRINITY_DN2329_c0_g1_i9	No hit	0.2	14.1
731	TRINITY_DN833_c0_g2_i1	No hit	1.0	14.1
732	TRINITY_DN6707_c0_g5_i1	No hit	0.2	14.0
733	TRINITY_DN14795_c0_g1_i26	Katanin p60 ATPase-containing subunit A-like 1 isoform X1	1.4	14.0
734	TRINITY_DN1771_c0_g1_i1	Protein MLP1 homolog isoform X3	0.6	14.0
735	TRINITY_DN2138_c0_g1_i1	No hit	0.7	14.0
736	TRINITY_DN41417_c0_g1_i1	No hit	0.4	14.0
737	TRINITY_DN18275_c0_g1_i1	No hit	0.3	14.0
738	TRINITY_DN47426_c0_g1_i1	No hit	0.6	13.9
739	TRINITY_DN885_c0_g1_i1	Transcriptional protein SWT1 isoform X1	1.1	13.9
740	TRINITY_DN6991_c1_g1_i1	No hit	1.1	13.9
741	TRINITY_DN32707_c0_g1_i1	No hit	0.5	13.9
742	TRINITY_DN509_c0_g1_i2	Uncharacterized protein LOC106686849	1.8	13.9
743	TRINITY_DN40157_c0_g1_i1	No hit	1.3	13.8
744	TRINITY_DN1351_c0_g1_i6	No hit	1.9	13.8
745	TRINITY_DN933_c0_g1_i2	Prestin-like	0.6	13.8
746	TRINITY_DN43650_c0_g1_i1	No hit	0.0	13.8
747	TRINITY_DN37468_c0_g1_i1	No hit	0.0	13.8
748	TRINITY_DN23145_c0_g1_i1	No hit	1.5	13.8
749	TRINITY_DN4231_c0_g1_i1	Innexin inx1	1.1	13.8
750	TRINITY_DN57070_c0_g1_i1	No hit	0.5	13.8
751	TRINITY_DN6707_c0_g4_i1	No hit	0.3	13.7
752	TRINITY_DN56655_c0_g1_i1	No hit	0.6	13.7
753	TRINITY_DN147_c2_g1_i1	Proton-coupled amino acid transporter-like protein CG1139	0.0	13.7
754	TRINITY_DN1666_c0_g1_i1	Heparan sulfate 2-O-sulfotransferase pipe	0.4	13.6
755	TRINITY_DN31926_c0_g1_i1	No hit	0.8	13.5
756	TRINITY_DN4224_c0_g2_i3	Uncharacterized protein LOC106686688	0.0	13.5
757	TRINITY_DN31242_c0_g1_i1	No hit	0.8	13.5
758	TRINITY_DN15489_c0_g2_i1	No hit	0.2	13.3
759	TRINITY_DN4757_c0_g3_i4	No hit	1.8	13.3
760	TRINITY_DN38823_c0_g1_i1	No hit	0.7	13.3
761	TRINITY_DN18275_c0_g1_i2	No hit	0.2	13.3
762	TRINITY_DN1686_c2_g4_i1	Tubulin-specific chaperone D	0.7	13.3
763	TRINITY_DN28828_c0_g1_i1	No hit	0.0	13.3
764	TRINITY_DN19302_c0_g1_i1	No hit	0.3	13.3
765	TRINITY_DN14573_c0_g1_i3	Cathepsin B-like	0.4	13.3
766	TRINITY_DN10561_c0_g1_i1	No hit	0.7	13.2

767	TRINITY_DN26348_c0_g1_i1	No hit	0.4	13.2
768	TRINITY_DN2472_c0_g1_i1	Protein FAM43A	1.3	13.1
769	TRINITY_DN105_c0_g1_i11	Serine/threonine-protein phosphatase 6 regulatory ankyrin repeat subunit A-like	0.2	13.1
770	TRINITY_DN53642_c0_g1_i1	Transient receptor potential cation channel subfamily A member 1 isoform X3	0.2	13.1
771	TRINITY_DN46959_c0_g1_i1	Uncharacterized protein LOC106692761 isoform X2	2.3	13.1
772	TRINITY_DN19009_c0_g1_i1	No hit	0.5	13.1
773	TRINITY_DN14086_c1_g2_i1	No hit	0.2	13.0
774	TRINITY_DN30625_c1_g1_i1	No hit	0.0	13.0
775	TRINITY_DN9313_c0_g3_i1	Fringe glycosyltransferase	2.4	13.0
776	TRINITY_DN6885_c0_g1_i1	No hit	0.4	13.0
777	TRINITY_DN1727_c0_g1_i1	No hit	2.0	13.0
778	TRINITY_DN6707_c0_g1_i1	No hit	0.0	13.0
779	TRINITY_DN243_c0_g1_i40	Uncharacterized protein LOC106687821 isoform X7	0.3	13.0
780	TRINITY_DN28895_c0_g1_i1	No hit	0.0	13.0
781	TRINITY_DN9792_c0_g1_i1	No hit	1.0	12.9
782	TRINITY_DN918_c0_g1_i1	No hit	0.7	12.9
783	TRINITY_DN35882_c0_g1_i1	No hit	0.6	12.8
784	TRINITY_DN11824_c0_g2_i1	No hit	0.6	12.8
785	TRINITY_DN10945_c1_g1_i1	No hit	0.4	12.8
786	TRINITY_DN45714_c0_g1_i1	No hit	0.5	12.7
787	TRINITY_DN5785_c0_g1_i7	SEC14-like protein 2 isoform X1	0.5	12.7
788	TRINITY_DN41056_c0_g1_i1	No hit	0.0	12.7
789	TRINITY_DN16916_c0_g1_i1	No hit	0.0	12.7
790	TRINITY_DN56112_c0_g1_i1	No hit	0.1	12.6
791	TRINITY_DN14830_c0_g1_i1	No hit	1.4	12.6
792	TRINITY_DN1306_c0_g1_i1	Uncharacterized protein LOC106686186 isoform X3	0.9	12.5
793	TRINITY_DN6980_c0_g1_i2	Nose resistant to fluoxetine protein 6-like isoform X1	0.4	12.5
794	TRINITY_DN1362_c0_g1_i2	No hit	0.4	12.4
795	TRINITY_DN3773_c0_g1_i9	No hit	1.2	12.4
796	TRINITY_DN11734_c0_g1_i1	Uncharacterized protein LOC106682289 isoform X2	0.6	12.4
797	TRINITY_DN2201_c0_g1_i1	Alpha-tocopherol transfer protein-like	0.3	12.4
798	TRINITY_DN3781_c0_g2_i1	No hit	0.2	12.3
799	TRINITY_DN8180_c0_g1_i1	No hit	0.9	12.3
800	TRINITY_DN3481_c0_g1_i3	Uncharacterized protein LOC106683278 isoform X2	1.1	12.3
801	TRINITY_DN58_c0_g1_i9	No hit	0.2	12.3
802	TRINITY_DN6323_c0_g1_i1	Multiple inositol polyphosphate phosphatase 1 isoform X1	0.6	12.3
803	TRINITY_DN890_c0_g1_i2	Uncharacterized protein LOC106692969	0.3	12.3
804	TRINITY_DN17115_c2_g1_i2	No hit	0.2	12.2
805	TRINITY_DN16556_c0_g2_i1	No hit	0.5	12.2
806	TRINITY_DN4808_c0_g1_i2	Onchocystatin	2.4	12.2
807	TRINITY_DN3623_c0_g1_i1	No hit	0.4	12.2
808	TRINITY_DN36325_c0_g1_i1	No hit	0.0	12.2
809	TRINITY_DN15872_c0_g1_i1	No hit	1.0	12.1
810	TRINITY_DN5024_c1_g3_i1	No hit	0.2	12.1
811	TRINITY_DN116_c1_g1_i1	Proteoglycan Cow	1.8	12.1
812	TRINITY_DN631_c0_g1_i1	PDZ and LIM domain protein Zasp isoform X2	0.9	12.1
813	TRINITY_DN49137_c0_g1_i1	No hit	0.1	12.0
814	TRINITY_DN16828_c0_g1_i1	No hit	0.9	12.0
815	TRINITY_DN20756_c0_g3_i3	Uncharacterized protein LOC106690952	2.0	12.0
816	TRINITY_DN651_c0_g1_i3	NADP-dependent malic enzyme isoform X1	1.0	12.0
817	TRINITY_DN18724_c0_g2_i1	No hit	0.2	11.9
818	TRINITY_DN40202_c0_g1_i1	No hit	0.1	11.9
819	TRINITY_DN1497_c0_g1_i1	Protein eva-1-like isoform X1	2.2	11.9
820	TRINITY_DN14388_c0_g1_i1	Laminin subunit gamma-1 isoform X2	1.8	11.9
821	TRINITY_DN2381_c0_g1_i6	NADP-dependent malic enzyme-like isoform X1	0.0	11.9
822	TRINITY_DN3491_c0_g1_i1	Alpha-tocopherol transfer protein-like	0.3	11.8
823	TRINITY_DN20826_c0_g1_i1	No hit	1.1	11.8
824	TRINITY_DN1695_c0_g1_i31	Golgin subfamily A member 5	0.9	11.8
825	TRINITY_DN53103_c0_g1_i1	No hit	0.3	11.8
826	TRINITY_DN25732_c0_g1_i1	No hit	0.3	11.7
827	TRINITY_DN1259_c0_g1_i12	Protein phosphatase PP2A 55 kDa regulatory subunit isoform X1	0.2	11.7
828	TRINITY_DN14264_c0_g2_i1	Titin isoform X5	0.5	11.7
829	TRINITY_DN48718_c0_g1_i1	No hit	0.9	11.7
830	TRINITY_DN26795_c0_g1_i1	No hit	0.4	11.7
831	TRINITY_DN38473_c0_g1_i1	No hit	0.8	11.6
832	TRINITY_DN22387_c0_g1_i1	No hit	0.7	11.6
833	TRINITY_DN5978_c0_g2_i1	No hit	0.5	11.6
834	TRINITY_DN44112_c0_g1_i1	No hit	0.6	11.6
835	TRINITY_DN15203_c0_g1_i1	No hit	0.6	11.6
836	TRINITY_DN22416_c0_g1_i1	No hit	0.2	11.5
837	TRINITY_DN426_c0_g1_i4	Huntingtin-interacting protein 1 isoform X1	0.2	11.5
838	TRINITY_DN2352_c0_g1_i11	Uncharacterized protein LOC106687459 isoform X1	1.8	11.4
839	TRINITY_DN980_c3_g1_i1	No hit	0.5	11.4
840	TRINITY_DN16337_c0_g3_i1	No hit	1.1	11.4
841	TRINITY_DN14264_c0_g1_i1	Titin isoform X5	0.6	11.4
842	TRINITY_DN36607_c0_g1_i1	No hit	0.3	11.4
843	TRINITY_DN14859_c0_g2_i1	No hit	0.1	11.4

844	TRINITY_DN10007_c0_g1_i1	No hit	0.6	11.4
845	TRINITY_DN10450_c0_g1_i1	No hit	0.2	11.3
846	TRINITY_DN7523_c0_g1_i7	No hit	0.5	11.3
847	TRINITY_DN25732_c0_g2_i1	No hit	0.4	11.3
848	TRINITY_DN3826_c1_g1_i1	No hit	1.6	11.3
849	TRINITY_DN6014_c0_g1_i3	Ankyrin-3-like isoform X1	1.1	11.3
850	TRINITY_DN3176_c0_g1_i6	Thioredoxin, mitochondrial	1.6	11.2
851	TRINITY_DN38329_c0_g1_i1	No hit	0.0	11.2
852	TRINITY_DN41877_c0_g1_i1	No hit	0.5	11.2
853	TRINITY_DN29350_c0_g1_i1	Uncharacterized protein LOC106681750	0.7	11.2
854	TRINITY_DN5746_c0_g1_i1	Zinc transporter ZIP1-like	1.1	11.2
855	TRINITY_DN9878_c0_g2_i1	No hit	1.7	11.2
856	TRINITY_DN1793_c1_g1_i2	No hit	0.5	11.1
857	TRINITY_DN57159_c0_g1_i1	No hit	0.7	11.1
858	TRINITY_DN14077_c0_g1_i1	Uncharacterized protein LOC106682594	0.4	11.1
859	TRINITY_DN15514_c0_g2_i1	No hit	0.7	11.1
860	TRINITY_DN2472_c0_g2_i1	No hit	1.6	11.1
861	TRINITY_DN54231_c0_g1_i1	Angiotensin-converting enzyme-like	1.0	11.1
862	TRINITY_DN14264_c0_g1_i4	Titin isoform X5	0.4	11.0
863	TRINITY_DN6592_c0_g2_i1	No hit	0.9	11.0
864	TRINITY_DN737_c0_g1_i1	N-acetylgalactosaminyltransferase 7	2.2	11.0
865	TRINITY_DN29995_c0_g1_i1	No hit	0.3	11.0
866	TRINITY_DN35358_c0_g1_i1	No hit	0.5	11.0
867	TRINITY_DN1431_c0_g2_i8	Tropomodulin-1 isoform X6	0.0	11.0
868	TRINITY_DN10593_c0_g1_i2	Aldose reductase	1.8	10.9
869	TRINITY_DN58_c0_g1_i2	No hit	0.8	10.9
870	TRINITY_DN15955_c0_g1_i1	No hit	0.8	10.9
871	TRINITY_DN9361_c0_g1_i1	No hit	0.3	10.9
872	TRINITY_DN24289_c0_g1_i1	No hit	0.1	10.9
873	TRINITY_DN12803_c2_g3_i1	No hit	0.4	10.9
874	TRINITY_DN33231_c0_g1_i1	Solute carrier family 2, facilitated glucose transporter member 6	0.1	10.8
875	TRINITY_DN7917_c0_g1_i3	No hit	0.9	10.8
876	TRINITY_DN16991_c0_g2_i1	Extracellular sulfatase SULF-1 homolog isoform X2	0.6	10.8
877	TRINITY_DN23031_c0_g1_i6	Four and a half LIM domains protein 3 isoform X2	1.4	10.7
878	TRINITY_DN34994_c0_g1_i1	Uncharacterized protein LOC106677279	0.8	10.7
879	TRINITY_DN31431_c0_g2_i1	No hit	0.0	10.7
880	TRINITY_DN25101_c0_g1_i1	No hit	0.6	10.7
881	TRINITY_DN5050_c0_g1_i2	Uncharacterized protein LOC106683235 isoform X2	0.7	10.7
882	TRINITY_DN50560_c0_g1_i1	No hit	0.6	10.7
883	TRINITY_DN48135_c0_g1_i1	No hit	0.5	10.6
884	TRINITY_DN19052_c3_g1_i1	No hit	0.0	10.6
885	TRINITY_DN3885_c0_g1_i30	Group XV phospholipase A2-like	1.5	10.6
886	TRINITY_DN31388_c0_g1_i1	No hit	0.0	10.6
887	TRINITY_DN35722_c0_g1_i1	No hit	0.7	10.6
888	TRINITY_DN6707_c0_g2_i1	No hit	0.2	10.6
889	TRINITY_DN10897_c0_g1_i1	No hit	0.4	10.6
890	TRINITY_DN8437_c0_g1_i1	No hit	0.3	10.5
891	TRINITY_DN179_c1_g1_i2	No hit	2.2	10.5
892	TRINITY_DN43180_c0_g1_i1	No hit	0.2	10.4
893	TRINITY_DN17921_c0_g1_i1	No hit	0.7	10.4
894	TRINITY_DN4011_c1_g1_i1	No hit	0.4	10.4
895	TRINITY_DN44094_c0_g1_i1	No hit	0.2	10.4
896	TRINITY_DN37530_c0_g1_i1	No hit	0.2	10.4
897	TRINITY_DN51533_c0_g1_i1	No hit	0.8	10.4
898	TRINITY_DN35111_c0_g1_i1	No hit	0.6	10.4
899	TRINITY_DN11742_c0_g1_i1	Uncharacterized protein LOC106678613	0.5	10.4
900	TRINITY_DN19034_c0_g1_i1	No hit	0.4	10.4
901	TRINITY_DN4072_c0_g2_i1	Cytochrome P450 6a2-like isoform X3	1.2	10.4
902	TRINITY_DN19016_c1_g1_i1	No hit	0.6	10.4
903	TRINITY_DN3072_c2_g1_i2	Neuroendocrine protein 7B2 isoform X2	1.0	10.4
904	TRINITY_DN41_c1_g2_i1	Proton-coupled amino acid transporter-like protein pathetic isoform X4	1.6	10.3
905	TRINITY_DN11377_c0_g1_i1	No hit	0.3	10.3
906	TRINITY_DN719_c0_g1_i2	Sphingosine-1-phosphate phosphatase 2	0.4	10.3
907	TRINITY_DN1269_c1_g2_i1	No hit	1.9	10.3
908	TRINITY_DN1345_c0_g1_i7	Uncharacterized protein LOC106690248	0.3	10.3
909	TRINITY_DN11674_c0_g1_i1	No hit	1.2	10.3
910	TRINITY_DN3855_c0_g1_i4	Myosin-1B	1.5	10.3
911	TRINITY_DN40547_c0_g1_i1	No hit	0.5	10.2
912	TRINITY_DN54301_c0_g1_i1	No hit	0.8	10.2
913	TRINITY_DN50383_c0_g1_i1	No hit	0.5	10.2
914	TRINITY_DN20400_c0_g1_i3	No hit	0.0	10.1
915	TRINITY_DN9425_c0_g1_i2	No hit	1.0	10.1
916	TRINITY_DN955_c1_g2_i1	No hit	1.4	10.1
917	TRINITY_DN10637_c0_g1_i1	No hit	0.9	10.1
918	TRINITY_DN38981_c0_g1_i1	No hit	0.0	10.1
919	TRINITY_DN32772_c0_g1_i1	No hit	0.2	10.1
920	TRINITY_DN49935_c0_g1_i1	Digestive cysteine proteinase 1	1.2	10.0

921	TRINITY_DN48758_c0_g1_i1	No hit	0.4	10.0
922	TRINITY_DN37393_c0_g1_i1	No hit	0.3	10.0
923	TRINITY_DN50380_c0_g1_i1	No hit	0.7	10.0
924	TRINITY_DN22573_c0_g1_i1	No hit	1.1	10.0
925	TRINITY_DN2574_c0_g2_i2	No hit	0.6	9.9
926	TRINITY_DN3213_c0_g1_i1	No hit	1.6	9.9
927	TRINITY_DN8546_c0_g1_i1	No hit	0.4	9.9
928	TRINITY_DN2201_c0_g1_i5	Alpha-tocopherol transfer protein-like	0.1	9.9
929	TRINITY_DN34869_c0_g1_i1	Glutathione hydrolase 1 proenzyme isoform X2	0.4	9.8
930	TRINITY_DN14623_c0_g1_i3	No hit	0.1	9.8
931	TRINITY_DN33850_c1_g1_i1	No hit	0.0	9.8
932	TRINITY_DN20079_c0_g1_i1	UDP-glucuronosyltransferase 2B17-like	0.1	9.8
933	TRINITY_DN873_c0_g2_i1	Semaphorin-1A-like	0.5	9.8
934	TRINITY_DN8317_c1_g1_i1	Ankyrin repeat domain-containing protein 1-like	1.0	9.8
935	TRINITY_DN23246_c0_g1_i1	No hit	0.0	9.8
936	TRINITY_DN12974_c0_g1_i1	No hit	0.6	9.8
937	TRINITY_DN6093_c0_g1_i3	Ephrin type-B receptor 1-B	0.6	9.7
938	TRINITY_DN7207_c0_g1_i1	No hit	1.2	9.7
939	TRINITY_DN975_c0_g1_i4	No hit	0.1	9.7
940	TRINITY_DN9109_c0_g2_i1	No hit	0.1	9.7
941	TRINITY_DN37115_c0_g1_i1	Uncharacterized protein LOC106690329	0.2	9.7
942	TRINITY_DN7358_c0_g1_i2	Ras-responsive element-binding protein 1-like	1.0	9.6
943	TRINITY_DN15523_c0_g1_i1	No hit	0.6	9.6
944	TRINITY_DN27288_c0_g1_i1	No hit	0.7	9.6
945	TRINITY_DN975_c0_g1_i3	No hit	0.4	9.6
946	TRINITY_DN50393_c0_g1_i1	No hit	0.3	9.6
947	TRINITY_DN28297_c0_g1_i1	No hit	0.3	9.6
948	TRINITY_DN22266_c0_g1_i1	No hit	0.0	9.5
949	TRINITY_DN22741_c0_g1_i1	No hit	0.2	9.5
950	TRINITY_DN29404_c0_g1_i1	No hit	0.0	9.5
951	TRINITY_DN2329_c0_g1_i5	Procardosin-A	0.1	9.5
952	TRINITY_DN15951_c0_g2_i3	No hit	1.0	9.5
953	TRINITY_DN44526_c0_g1_i1	No hit	0.4	9.5
954	TRINITY_DN2889_c0_g1_i7	Uncharacterized protein LOC106684374	0.6	9.4
955	TRINITY_DN13_c0_g1_i1	Glutathione S-transferase isoform X1	0.7	9.4
956	TRINITY_DN32151_c0_g1_i1	No hit	0.5	9.4
957	TRINITY_DN35730_c0_g1_i1	No hit	0.4	9.3
958	TRINITY_DN4285_c3_g1_i1	No hit	1.6	9.3
959	TRINITY_DN3736_c0_g3_i3	Thymosin beta isoform X1	1.4	9.3
960	TRINITY_DN2206_c0_g1_i16	Microtubule-actin cross-linking factor 1 isoform X8	1.8	9.3
961	TRINITY_DN6612_c0_g1_i1	No hit	0.3	9.2
962	TRINITY_DN833_c0_g1_i2	No hit	0.9	9.2
963	TRINITY_DN37101_c0_g1_i1	No hit	0.6	9.2
964	TRINITY_DN52119_c0_g1_i1	No hit	0.2	9.2
965	TRINITY_DN9104_c0_g1_i1	No hit	1.3	9.2
966	TRINITY_DN9154_c0_g1_i1	Monocarboxylate transporter 9 isoform X4	1.4	9.2
967	TRINITY_DN25197_c0_g1_i1	No hit	0.3	9.2
968	TRINITY_DN23031_c0_g1_i1	Four and a half LIM domains protein 3 isoform X2	0.2	9.1
969	TRINITY_DN9593_c0_g1_i6	No hit	0.4	9.1
970	TRINITY_DN7970_c0_g1_i4	Ribonuclease Oy-like	1.0	9.1
971	TRINITY_DN38073_c0_g1_i1	No hit	0.2	9.1
972	TRINITY_DN32994_c0_g1_i1	No hit	0.0	9.1
973	TRINITY_DN30770_c0_g1_i1	No hit	0.4	9.1
974	TRINITY_DN613_c0_g1_i3	Protein ref(2)P-like	0.6	9.0
975	TRINITY_DN37214_c0_g1_i1	No hit	0.0	9.0
976	TRINITY_DN29491_c0_g1_i1	No hit	0.1	9.0
977	TRINITY_DN6265_c0_g1_i1	Uncharacterized protein LOC106692099	0.2	9.0
978	TRINITY_DN320_c1_g1_i1	Putative mediator of RNA polymerase II transcription subunit 26 isoform X1	1.4	9.0
979	TRINITY_DN14547_c0_g1_i1	Acyl-CoA synthetase family member 2, mitochondrial	0.8	9.0
980	TRINITY_DN24515_c0_g1_i1	No hit	0.0	8.9
981	TRINITY_DN3034_c0_g1_i1	No hit	0.6	8.9
982	TRINITY_DN4737_c0_g1_i1	Uncharacterized protein LOC106679752 isoform X1	0.3	8.9
983	TRINITY_DN3053_c0_g1_i9	Uncharacterized protein LOC106682645 isoform X1	1.5	8.8
984	TRINITY_DN116_c0_g1_i19	No hit	0.0	8.8
985	TRINITY_DN396_c0_g2_i2	No hit	0.0	8.8
986	TRINITY_DN14293_c0_g1_i1	No hit	1.1	8.8
987	TRINITY_DN76_c0_g1_i8	Phospholipid scramblase family member 5-like	1.5	8.8
988	TRINITY_DN53884_c0_g1_i1	No hit	0.2	8.8
989	TRINITY_DN11621_c1_g1_i1	No hit	1.3	8.7
990	TRINITY_DN1727_c0_g2_i1	No hit	1.8	8.7
991	TRINITY_DN11824_c0_g1_i1	No hit	0.8	8.7
992	TRINITY_DN23186_c0_g1_i1	No hit	0.4	8.7
993	TRINITY_DN40199_c0_g1_i1	No hit	0.5	8.7
994	TRINITY_DN28073_c0_g1_i1	No hit	0.7	8.6
995	TRINITY_DN19240_c0_g1_i3	Uncharacterized protein LOC106681209, partial	0.4	8.6
996	TRINITY_DN29727_c0_g2_i1	No hit	0.0	8.6
997	TRINITY_DN1474_c1_g1_i1	No hit	0.5	8.6

998	TRINITY_DN12321_c0_g1_i1	No hit	0.9	8.6
999	TRINITY_DN30444_c0_g1_i1	No hit	1.4	8.6
1000	TRINITY_DN1844_c0_g1_i1	Sonic hedgehog protein A	0.5	8.6
1001	TRINITY_DN3885_c0_g1_i1	Group XV phospholipase A2-like	0.0	8.5
1002	TRINITY_DN8066_c0_g1_i4	Tyrosine-protein kinase Fer isoform X2	1.3	8.5
1003	TRINITY_DN5358_c0_g1_i1	No hit	0.2	8.5
1004	TRINITY_DN37531_c0_g1_i1	No hit	0.3	8.5
1005	TRINITY_DN28995_c0_g1_i1	Protein FAM151B isoform X3	0.0	8.5
1006	TRINITY_DN14760_c0_g2_i1	UDP-glucuronosyltransferase	1.3	8.4
1007	TRINITY_DN25099_c0_g1_i1	Cyclin-dependent kinase 14 isoform X1	1.2	8.4
1008	TRINITY_DN9871_c0_g1_i3	Protein yellow-like	0.6	8.4
1009	TRINITY_DN3736_c0_g4_i1	No hit	0.5	8.3
1010	TRINITY_DN11180_c0_g1_i3	Supervillin-like isoform X4	0.0	8.3
1011	TRINITY_DN19118_c0_g1_i1	No hit	0.0	8.3
1012	TRINITY_DN50984_c0_g1_i1	No hit	0.4	8.3
1013	TRINITY_DN41964_c0_g1_i1	No hit	0.0	8.3
1014	TRINITY_DN2262_c0_g1_i13	No hit	0.2	8.3
1015	TRINITY_DN14759_c0_g1_i1	No hit	0.6	8.3
1016	TRINITY_DN56972_c0_g1_i1	No hit	0.0	8.3
1017	TRINITY_DN20807_c0_g1_i1	No hit	0.1	8.3
1018	TRINITY_DN6598_c0_g2_i1	No hit	0.2	8.3
1019	TRINITY_DN3762_c1_g1_i1	No hit	1.4	8.3
1020	TRINITY_DN10306_c0_g1_i1	No hit	0.1	8.3
1021	TRINITY_DN29658_c0_g1_i1	No hit	0.0	8.3
1022	TRINITY_DN7847_c0_g1_i1	Insulin-like growth factor-binding protein complex acid labile subunit	0.6	8.2
1023	TRINITY_DN473_c0_g1_i6	Elongation of very long chain fatty acids protein AAEL008004 isoform X2	0.0	8.2
1024	TRINITY_DN6081_c2_g1_i1	No hit	0.6	8.2
1025	TRINITY_DN17898_c3_g1_i1	No hit	0.2	8.2
1026	TRINITY_DN54568_c0_g1_i1	Facilitated trehalose transporter Tret1-like isoform X2	0.9	8.2
1027	TRINITY_DN44868_c0_g1_i1	No hit	0.0	8.2
1028	TRINITY_DN34546_c0_g1_i1	Uncharacterized protein LOC106685450	0.0	8.2
1029	TRINITY_DN1906_c0_g1_i4	Transient receptor potential channel pyrexia-like	0.1	8.2
1030	TRINITY_DN44942_c0_g1_i1	No hit	0.2	8.2
1031	TRINITY_DN2329_c0_g1_i4	No hit	0.1	8.2
1032	TRINITY_DN27734_c0_g1_i1	No hit	0.7	8.2
1033	TRINITY_DN3736_c0_g2_i1	No hit	0.1	8.1
1034	TRINITY_DN10662_c0_g1_i3	No hit	0.9	8.1
1035	TRINITY_DN25089_c0_g1_i1	General odorant-binding protein 70	1.1	8.1
1036	TRINITY_DN48210_c0_g1_i1	Uncharacterized protein LOC106679017	0.4	8.1
1037	TRINITY_DN42440_c0_g1_i1	No hit	0.1	8.1
1038	TRINITY_DN2499_c0_g1_i3	No hit	0.5	8.1
1039	TRINITY_DN14050_c0_g1_i1	No hit	0.6	8.1
1040	TRINITY_DN23769_c0_g1_i1	No hit	0.0	8.1
1041	TRINITY_DN51385_c0_g1_i1	No hit	0.2	8.1
1042	TRINITY_DN116_c0_g1_i12	Probable E3 ubiquitin-protein ligase MGRN1 isoform X2	0.0	8.1
1043	TRINITY_DN5173_c0_g1_i2	Alpha-aminoadipic semialdehyde dehydrogenase	1.3	8.1
1044	TRINITY_DN1362_c0_g1_i6	Oxidation resistance protein 1 isoform X8	0.0	8.0
1045	TRINITY_DN25840_c0_g3_i1	No hit	0.2	8.0
1046	TRINITY_DN45949_c0_g1_i1	No hit	0.3	8.0
1047	TRINITY_DN6004_c0_g1_i1	Pancreatic triacylglycerol lipase-like	0.4	8.0
1048	TRINITY_DN1841_c0_g1_i3	Headcase protein-like, partial	1.3	8.0
1049	TRINITY_DN12803_c2_g2_i1	No hit	1.2	8.0
1050	TRINITY_DN3028_c1_g1_i1	No hit	0.5	8.0
1051	TRINITY_DN45145_c0_g1_i1	No hit	0.4	8.0
1052	TRINITY_DN34832_c0_g1_i1	No hit	0.7	8.0
1053	TRINITY_DN50354_c0_g1_i1	Esterase FE4-like	0.2	8.0
1054	TRINITY_DN2598_c0_g2_i2	No hit	0.5	7.9
1055	TRINITY_DN26773_c0_g1_i1	No hit	0.0	7.9
1056	TRINITY_DN13991_c0_g1_i1	No hit	0.0	7.9
1057	TRINITY_DN12708_c0_g1_i1	No hit	0.5	7.9
1058	TRINITY_DN4431_c0_g1_i1	No hit	0.6	7.9
1059	TRINITY_DN35068_c0_g1_i1	Protein kinase DC2	0.7	7.9
1060	TRINITY_DN41610_c0_g1_i1	No hit	0.3	7.9
1061	TRINITY_DN51949_c0_g1_i1	No hit	0.2	7.9
1062	TRINITY_DN44119_c0_g1_i1	No hit	0.2	7.8
1063	TRINITY_DN27416_c0_g1_i1	No hit	0.0	7.8
1064	TRINITY_DN4516_c0_g3_i1	No hit	0.8	7.8
1065	TRINITY_DN32211_c0_g2_i1	No hit	0.5	7.8
1066	TRINITY_DN1236_c0_g1_i5	No hit	0.1	7.8
1067	TRINITY_DN5241_c0_g1_i1	Very low-density lipoprotein receptor-like isoform X1	1.3	7.7
1068	TRINITY_DN51911_c0_g1_i1	No hit	0.0	7.7
1069	TRINITY_DN21968_c0_g1_i1	No hit	0.3	7.7
1070	TRINITY_DN7358_c0_g1_i3	Ras-responsive element-binding protein 1-like	0.7	7.7
1071	TRINITY_DN22331_c0_g1_i1	No hit	0.3	7.7
1072	TRINITY_DN1227_c0_g1_i5	Serine/threonine-protein kinase tricorner isoform X1	0.5	7.6
1073	TRINITY_DN20113_c0_g1_i1	No hit	0.6	7.6
1074	TRINITY_DN13609_c0_g1_i1	No hit	0.5	7.6

1075	TRINITY_DN8599_c0_g1_i2	No hit	1.5	7.6
1076	TRINITY_DN26103_c0_g1_i1	No hit	0.1	7.6
1077	TRINITY_DN21445_c0_g2_i1	Angiotensin-converting enzyme-like	0.6	7.6
1078	TRINITY_DN2259_c0_g1_i3	No hit	0.0	7.6
1079	TRINITY_DN49923_c0_g1_i1	No hit	0.9	7.6
1080	TRINITY_DN19367_c0_g1_i2	No hit	0.8	7.6
1081	TRINITY_DN3049_c0_g1_i2	Mid1-interacting protein 1-B	1.1	7.6
1082	TRINITY_DN2206_c0_g1_i28	No hit	1.4	7.6
1083	TRINITY_DN43589_c0_g1_i1	No hit	0.0	7.6
1084	TRINITY_DN1229_c2_g1_i1	No hit	0.6	7.5
1085	TRINITY_DN23397_c1_g1_i1	No hit	0.6	7.5
1086	TRINITY_DN42870_c0_g1_i1	No hit	0.3	7.5
1087	TRINITY_DN11006_c0_g1_i1	Uncharacterized protein LOC106689204	0.5	7.5
1088	TRINITY_DN419_c2_g1_i1	No hit	0.0	7.5
1089	TRINITY_DN23449_c0_g1_i1	No hit	0.0	7.4
1090	TRINITY_DN2259_c0_g1_i1	Cytochrome P450 6a2-like isoform X1	0.7	7.4
1091	TRINITY_DN27587_c0_g1_i1	No hit	0.0	7.4
1092	TRINITY_DN5577_c0_g2_i1	No hit	0.1	7.4
1093	TRINITY_DN9037_c0_g1_i1	No hit	0.4	7.4
1094	TRINITY_DN9593_c0_g1_i4	No hit	0.3	7.4
1095	TRINITY_DN44357_c0_g1_i1	No hit	0.5	7.3
1096	TRINITY_DN6707_c0_g3_i6	No hit	0.0	7.3
1097	TRINITY_DN624_c0_g1_i4	Uncharacterized protein LOC106688735 isoform X1	1.3	7.3
1098	TRINITY_DN3084_c0_g2_i2	No hit	0.4	7.3
1099	TRINITY_DN11787_c0_g2_i1	No hit	0.3	7.3
1100	TRINITY_DN1142_c0_g1_i1	No hit	1.3	7.3
1101	TRINITY_DN49588_c0_g1_i1	No hit	0.4	7.3
1102	TRINITY_DN38382_c0_g1_i1	No hit	0.0	7.3
1103	TRINITY_DN4523_c0_g1_i1	Uncharacterized protein LOC106690537 isoform X1	1.0	7.3
1104	TRINITY_DN3131_c0_g1_i2	Ets DNA-binding protein pokkuri	0.0	7.2
1105	TRINITY_DN42083_c0_g1_i1	No hit	0.1	7.2
1106	TRINITY_DN48557_c0_g1_i1	No hit	0.3	7.2
1107	TRINITY_DN16801_c0_g1_i1	No hit	0.1	7.2
1108	TRINITY_DN3530_c0_g3_i1	No hit	1.1	7.2
1109	TRINITY_DN1360_c0_g1_i1	Transforming growth factor-beta-induced protein ig-h3 isoform X1	0.9	7.2
1110	TRINITY_DN1227_c0_g1_i4	Serine/threonine-protein kinase tricorner isoform X1	0.6	7.2
1111	TRINITY_DN509_c0_g2_i1	No hit	0.5	7.2
1112	TRINITY_DN2872_c1_g1_i5	Uncharacterized protein LOC106682438 isoform X2	0.3	7.2
1113	TRINITY_DN3736_c0_g1_i1	No hit	0.2	7.2
1114	TRINITY_DN2613_c0_g1_i4	Serpin B4-like	0.3	7.2
1115	TRINITY_DN2858_c0_g1_i1	No hit	1.2	7.1
1116	TRINITY_DN14391_c0_g1_i1	No hit	0.1	7.1
1117	TRINITY_DN20152_c0_g1_i1	No hit	0.2	7.1
1118	TRINITY_DN42171_c0_g1_i1	No hit	0.4	7.1
1119	TRINITY_DN5649_c0_g1_i3	Structural maintenance of chromosomes protein 3-like	0.0	7.1
1120	TRINITY_DN46115_c0_g1_i1	No hit	0.4	7.1
1121	TRINITY_DN35801_c0_g1_i1	No hit	0.4	7.1
1122	TRINITY_DN10796_c0_g2_i1	No hit	0.8	7.0
1123	TRINITY_DN10671_c0_g2_i1	Androgen-dependent TFPI-regulating protein-like	0.4	7.0
1124	TRINITY_DN194_c0_g2_i2	No hit	0.2	7.0
1125	TRINITY_DN16991_c0_g3_i1	Extracellular sulfatase SULF-1 homolog isoform X1	0.9	7.0
1126	TRINITY_DN21890_c0_g1_i1	No hit	0.5	7.0
1127	TRINITY_DN16991_c0_g1_i1	Extracellular sulfatase SULF-1 homolog isoform X2	0.5	7.0
1128	TRINITY_DN50418_c0_g1_i1	No hit	0.0	7.0
1129	TRINITY_DN1481_c0_g2_i3	Adenylyl cyclase-associated protein 1 isoform X1	0.2	7.0
1130	TRINITY_DN16303_c0_g1_i1	No hit	0.3	6.9
1131	TRINITY_DN53008_c0_g1_i1	No hit	0.0	6.9
1132	TRINITY_DN7059_c0_g3_i1	No hit	0.6	6.9
1133	TRINITY_DN411_c0_g1_i1	Uncharacterized protein LOC106680845 isoform X2	0.7	6.9
1134	TRINITY_DN22327_c0_g1_i1	No hit	0.4	6.8
1135	TRINITY_DN39886_c0_g1_i1	No hit	0.0	6.8
1136	TRINITY_DN4516_c0_g4_i1	No hit	1.1	6.8
1137	TRINITY_DN2208_c0_g1_i1	Uncharacterized protein LOC106690964	0.2	6.8
1138	TRINITY_DN11741_c1_g1_i1	No hit	0.4	6.8
1139	TRINITY_DN44824_c0_g1_i1	No hit	0.3	6.8
1140	TRINITY_DN29848_c0_g1_i1	No hit	0.0	6.8
1141	TRINITY_DN17249_c0_g1_i1	No hit	0.0	6.8
1142	TRINITY_DN2492_c0_g1_i6	Uncharacterized protein LOC106690988 isoform X3	0.1	6.8
1143	TRINITY_DN4694_c0_g1_i1	No hit	1.1	6.8
1144	TRINITY_DN22663_c0_g1_i1	No hit	0.0	6.7
1145	TRINITY_DN51812_c0_g1_i1	No hit	0.2	6.7
1146	TRINITY_DN6127_c0_g1_i1	Protein Malvolio-like isoform X2	0.2	6.7
1147	TRINITY_DN4047_c0_g4_i1	No hit	0.4	6.7
1148	TRINITY_DN54909_c0_g1_i1	No hit	0.4	6.7
1149	TRINITY_DN25089_c0_g2_i1	No hit	0.6	6.7
1150	TRINITY_DN3945_c0_g1_i5	Nostrin isoform X3	0.1	6.7
1151	TRINITY_DN3885_c0_g1_i20	Group XV phospholipase A2-like	0.1	6.6

1152	TRINITY_DN35_c0_g3_i1	Uncharacterized protein LOC106689029	0.7	6.6
1153	TRINITY_DN10831_c0_g1_i7	CD2-associated protein-like	1.3	6.6
1154	TRINITY_DN3151_c0_g1_i1	Vanin-like protein 2	0.2	6.6
1155	TRINITY_DN25252_c0_g2_i1	No hit	0.7	6.6
1156	TRINITY_DN4612_c0_g1_i2	PDZ and LIM domain protein 3 isoform X5	0.9	6.6
1157	TRINITY_DN55512_c0_g1_i1	Uncharacterized protein LOC106680487 isoform X1	0.2	6.6
1158	TRINITY_DN54420_c0_g1_i1	No hit	0.3	6.6
1159	TRINITY_DN27110_c0_g2_i1	Cytosolic carboxypeptidase 2-like	0.3	6.5
1160	TRINITY_DN14623_c1_g1_i4	No hit	0.3	6.5
1161	TRINITY_DN31300_c0_g1_i1	No hit	0.4	6.5
1162	TRINITY_DN14264_c0_g1_i2	Titin isoform X4	0.1	6.5
1163	TRINITY_DN51000_c0_g1_i1	No hit	0.0	6.5
1164	TRINITY_DN9920_c0_g1_i1	ATP-binding cassette sub-family G member 1-like	1.0	6.5
1165	TRINITY_DN2084_c0_g1_i2	MFS-type transporter SLC18B1 isoform X1	0.0	6.5
1166	TRINITY_DN20763_c0_g1_i1	No hit	0.0	6.5
1167	TRINITY_DN9920_c0_g2_i1	No hit	0.0	6.5
1168	TRINITY_DN2074_c0_g2_i1	No hit	0.1	6.4
1169	TRINITY_DN1497_c0_g2_i1	No hit	0.0	6.4
1170	TRINITY_DN9706_c0_g1_i1	No hit	0.4	6.4
1171	TRINITY_DN5160_c0_g1_i1	No hit	0.0	6.4
1172	TRINITY_DN38524_c0_g1_i1	Protein slit, partial	1.0	6.4
1173	TRINITY_DN16854_c0_g1_i1	No hit	0.2	6.4
1174	TRINITY_DN40126_c0_g1_i1	Beta-mannosidase	0.1	6.4
1175	TRINITY_DN613_c0_g1_i1	Protein ref(2)P-like	0.4	6.4
1176	TRINITY_DN4839_c0_g1_i1	Protein FAM92B isoform X1	0.5	6.4
1177	TRINITY_DN882_c0_g1_i2	No hit	0.0	6.3
1178	TRINITY_DN56162_c0_g1_i1	No hit	0.2	6.3
1179	TRINITY_DN8599_c0_g5_i1	No hit	1.0	6.3
1180	TRINITY_DN6487_c0_g1_i1	No hit	0.4	6.3
1181	TRINITY_DN9863_c0_g1_i2	No hit	0.4	6.3
1182	TRINITY_DN358_c0_g1_i2	SH2 domain-containing protein 4B isoform X1	0.6	6.3
1183	TRINITY_DN45573_c0_g1_i1	No hit	0.6	6.3
1184	TRINITY_DN47540_c0_g1_i1	No hit	0.1	6.3
1185	TRINITY_DN1653_c0_g2_i1	No hit	0.9	6.3
1186	TRINITY_DN56362_c0_g1_i1	No hit	0.6	6.3
1187	TRINITY_DN21507_c0_g1_i1	No hit	0.3	6.3
1188	TRINITY_DN3094_c0_g1_i1	No hit	1.0	6.3
1189	TRINITY_DN25616_c0_g1_i1	Multidrug resistance protein 1A-like	0.4	6.2
1190	TRINITY_DN8071_c0_g1_i1	No hit	0.9	6.2
1191	TRINITY_DN53999_c0_g1_i1	No hit	0.3	6.2
1192	TRINITY_DN32389_c0_g1_i1	No hit	0.4	6.2
1193	TRINITY_DN5014_c0_g1_i1	Protein rhomboid-like isoform X2	0.7	6.2
1194	TRINITY_DN29610_c0_g1_i1	No hit	0.4	6.2
1195	TRINITY_DN5066_c0_g1_i1	No hit	0.4	6.2
1196	TRINITY_DN4740_c0_g1_i4	Uncharacterized protein LOC112211488	0.1	6.2
1197	TRINITY_DN49096_c0_g1_i1	No hit	0.0	6.2
1198	TRINITY_DN53506_c0_g1_i1	No hit	0.1	6.1
1199	TRINITY_DN1497_c0_g1_i4	Protein eva-1-like isoform X1	0.7	6.1
1200	TRINITY_DN40948_c0_g1_i1	No hit	0.5	6.1
1201	TRINITY_DN55114_c0_g1_i1	No hit	0.2	6.1
1202	TRINITY_DN41_c1_g3_i1	Proton-coupled amino acid transporter-like protein pathetic isoform X4	0.2	6.1
1203	TRINITY_DN7289_c0_g1_i1	No hit	0.7	6.1
1204	TRINITY_DN13246_c0_g1_i3	No hit	0.5	6.1
1205	TRINITY_DN17463_c0_g1_i1	No hit	0.8	6.1
1206	TRINITY_DN1841_c0_g1_i1	Headcase protein	0.1	6.1
1207	TRINITY_DN105_c0_g1_i7	Serine/threonine-protein phosphatase 6 regulatory ankyrin repeat subunit A-like	0.3	6.1
1208	TRINITY_DN1889_c0_g1_i1	No hit	0.5	6.1
1209	TRINITY_DN7173_c0_g1_i2	Uncharacterized protein LOC106685703 isoform X3	0.4	6.1
1210	TRINITY_DN14521_c0_g1_i1	Ascorbate-specific transmembrane electron transporter 1-like	0.1	6.1
1211	TRINITY_DN13380_c0_g1_i1	No hit	0.4	6.1
1212	TRINITY_DN10554_c0_g1_i1	No hit	0.3	6.1
1213	TRINITY_DN37362_c0_g1_i1	No hit	0.2	6.1
1214	TRINITY_DN28917_c0_g1_i1	No hit	0.0	6.0
1215	TRINITY_DN789_c0_g2_i3	No hit	0.8	6.0
1216	TRINITY_DN9824_c0_g1_i1	No hit	0.1	6.0
1217	TRINITY_DN12104_c0_g1_i1	No hit	0.4	6.0
1218	TRINITY_DN1229_c0_g2_i1	No hit	0.0	6.0
1219	TRINITY_DN19282_c3_g1_i1	No hit	0.7	6.0
1220	TRINITY_DN11243_c1_g3_i1	UDP-glucuronosyltransferase 2B23-like	0.6	6.0
1221	TRINITY_DN243_c0_g1_i28	Uncharacterized protein LOC106687821 isoform X12	0.0	6.0
1222	TRINITY_DN51556_c0_g1_i1	No hit	0.5	6.0
1223	TRINITY_DN42589_c0_g1_i1	No hit	0.0	5.9
1224	TRINITY_DN1182_c0_g1_i2	Uncharacterized protein LOC106686615 isoform X10	0.4	5.9
1225	TRINITY_DN34575_c0_g1_i1	No hit	0.2	5.9
1226	TRINITY_DN1214_c1_g1_i2	Multidrug resistance-associated protein 1-like	0.6	5.9
1227	TRINITY_DN32252_c0_g1_i1	No hit	0.1	5.9
1228	TRINITY_DN15221_c0_g1_i1	Scavenger receptor class B member 1 isoform X3	0.2	5.9

1229	TRINITY_DN49850_c0_g1_i1	Facilitated trehalose transporter Tret1-like	0.1	5.9
1230	TRINITY_DN29956_c0_g1_i1	No hit	0.0	5.9
1231	TRINITY_DN54492_c0_g1_i1	No hit	0.9	5.9
1232	TRINITY_DN13511_c0_g1_i2	No hit	0.3	5.9
1233	TRINITY_DN12232_c1_g2_i1	No hit	0.8	5.9
1234	TRINITY_DN50891_c0_g1_i1	No hit	0.3	5.8
1235	TRINITY_DN5121_c0_g1_i1	Latrophilin Cirl-like isoform X2	0.5	5.8
1236	TRINITY_DN1071_c1_g1_i1	No hit	0.3	5.8
1237	TRINITY_DN3532_c0_g1_i7	Ras-related protein Rab-5B	0.4	5.8
1238	TRINITY_DN6657_c0_g4_i1	No hit	0.0	5.8
1239	TRINITY_DN48963_c0_g1_i1	No hit	0.3	5.8
1240	TRINITY_DN34636_c0_g1_i1	No hit	0.1	5.8
1241	TRINITY_DN1_c0_g1_i3	Twitchin	0.7	5.8
1242	TRINITY_DN31127_c0_g1_i1	No hit	0.0	5.8
1243	TRINITY_DN18963_c0_g1_i1	No hit	0.1	5.7
1244	TRINITY_DN17881_c0_g1_i1	Multidrug resistance-associated protein 1	0.8	5.7
1245	TRINITY_DN41077_c0_g1_i1	No hit	0.4	5.7
1246	TRINITY_DN47267_c0_g1_i1	No hit	0.5	5.7
1247	TRINITY_DN2262_c0_g1_i10	Band 3 anion transport protein isoform X3	0.2	5.6
1248	TRINITY_DN17856_c0_g1_i1	No hit	0.2	5.6
1249	TRINITY_DN23459_c0_g1_i1	No hit	0.2	5.6
1250	TRINITY_DN1214_c1_g1_i1	Multidrug resistance-associated protein 1-like, partial	0.4	5.6
1251	TRINITY_DN9183_c2_g1_i1	No hit	0.2	5.6
1252	TRINITY_DN2992_c0_g1_i6	Uncharacterized protein LOC106686776	0.1	5.6
1253	TRINITY_DN12945_c0_g1_i2	No hit	0.3	5.6
1254	TRINITY_DN18838_c0_g1_i1	Uncharacterized protein LOC106691533	0.2	5.6
1255	TRINITY_DN5736_c0_g1_i1	No hit	0.0	5.6
1256	TRINITY_DN15007_c0_g1_i1	Uncharacterized protein LOC106691898	0.4	5.6
1257	TRINITY_DN48324_c0_g1_i1	No hit	0.3	5.6
1258	TRINITY_DN3670_c0_g1_i1	Myosin-10-like isoform X6	0.5	5.6
1259	TRINITY_DN9037_c0_g1_i2	RNA-binding protein MEX3B	0.8	5.6
1260	TRINITY_DN2195_c0_g1_i1	Prolow-density lipoprotein receptor-related protein 1 isoform X1	0.8	5.6
1261	TRINITY_DN1889_c0_g2_i1	No hit	0.4	5.6
1262	TRINITY_DN28158_c0_g1_i1	No hit	0.5	5.6
1263	TRINITY_DN6707_c0_g3_i2	No hit	0.1	5.5
1264	TRINITY_DN12085_c0_g1_i1	Juvenile hormone epoxide hydrolase 1-like	0.2	5.5
1265	TRINITY_DN6567_c0_g1_i3	Glycoprotein 3-alpha-L-fucosyltransferase A isoform X1	0.3	5.5
1266	TRINITY_DN19177_c0_g1_i1	No hit	0.3	5.5
1267	TRINITY_DN3885_c0_g1_i13	Group XV phospholipase A2-like	0.1	5.5
1268	TRINITY_DN16761_c0_g1_i1	Uncharacterized protein LOC106681156	0.2	5.5
1269	TRINITY_DN105_c0_g1_i2	Serine/threonine-protein phosphatase 6 regulatory ankyrin repeat subunit A-like	0.3	5.5
1270	TRINITY_DN35066_c0_g1_i1	No hit	0.6	5.5
1271	TRINITY_DN5799_c0_g1_i5	Protein cueball isoform X1	0.5	5.5
1272	TRINITY_DN28547_c0_g1_i1	No hit	0.1	5.5
1273	TRINITY_DN8066_c0_g1_i2	Tyrosine-protein kinase Fer isoform X2	0.6	5.4
1274	TRINITY_DN4734_c0_g1_i5	No hit	0.2	5.4
1275	TRINITY_DN21145_c0_g1_i1	No hit	0.2	5.4
1276	TRINITY_DN7745_c3_g1_i1	No hit	0.7	5.4
1277	TRINITY_DN50696_c0_g1_i1	No hit	0.0	5.4
1278	TRINITY_DN14736_c0_g1_i1	Integrin beta-PS isoform X2	0.6	5.4
1279	TRINITY_DN925_c0_g1_i14	Uncharacterized protein LOC106687334 isoform X4	0.7	5.4
1280	TRINITY_DN4844_c0_g1_i2	Transmembrane protein 135-like	0.4	5.4
1281	TRINITY_DN833_c0_g1_i1	Tubulin alpha-1 chain-like	0.0	5.3
1282	TRINITY_DN513_c0_g1_i5	Esterase FE4-like	0.1	5.3
1283	TRINITY_DN37909_c0_g1_i1	No hit	0.5	5.3
1284	TRINITY_DN7106_c2_g1_i5	Probable cytochrome P450 6a13	0.2	5.3
1285	TRINITY_DN31442_c0_g1_i1	No hit	0.4	5.3
1286	TRINITY_DN10570_c0_g1_i3	No hit	0.2	5.3
1287	TRINITY_DN14859_c0_g1_i6	Uncharacterized protein LOC106683312	0.1	5.3
1288	TRINITY_DN9109_c0_g1_i2	No hit	0.0	5.3
1289	TRINITY_DN29970_c0_g1_i1	No hit	0.3	5.2
1290	TRINITY_DN2574_c0_g1_i1	CCR4-NOT transcription complex subunit 10-like isoform X1	0.4	5.2
1291	TRINITY_DN35685_c0_g1_i1	No hit	0.1	5.2
1292	TRINITY_DN873_c0_g2_i2	Semaphorin-1A-like	1.0	5.2
1293	TRINITY_DN9499_c0_g1_i1	No hit	0.1	5.2
1294	TRINITY_DN49938_c0_g1_i1	No hit	0.1	5.2
1295	TRINITY_DN2262_c0_g1_i1	Band 3 anion transport protein isoform X5	0.5	5.2
1296	TRINITY_DN9451_c0_g1_i1	No hit	0.5	5.1
1297	TRINITY_DN28941_c0_g1_i1	No hit	0.3	5.1
1298	TRINITY_DN20826_c0_g2_i1	No hit	0.6	5.1
1299	TRINITY_DN17856_c0_g2_i1	Uncharacterized protein LOC106686739	0.4	5.1
1300	TRINITY_DN7316_c0_g1_i1	Uncharacterized protein LOC106678444	0.6	5.1
1301	TRINITY_DN2164_c0_g1_i2	No hit	0.5	5.1
1302	TRINITY_DN41489_c0_g1_i1	No hit	0.4	5.1
1303	TRINITY_DN7839_c0_g1_i1	No hit	0.0	5.1
1304	TRINITY_DN3484_c0_g1_i1	Solute carrier family 41 member 1 isoform X2	0.6	5.0
1305	TRINITY_DN21655_c0_g1_i1	Solute carrier family 23 member 1	0.5	5.0

1306	TRINITY_DN50173_c0_g1_i1	No hit	0.1	5.0
1307	TRINITY_DN3020_c0_g1_i2	No hit	0.2	5.0
1308	TRINITY_DN24936_c0_g1_i1	No hit	0.2	4.9
1309	TRINITY_DN25971_c0_g1_i1	No hit	0.2	4.9
1310	TRINITY_DN9979_c0_g2_i1	No hit	0.7	4.9
1311	TRINITY_DN5072_c0_g2_i1	Adenylate cyclase type 8-like isoform X1	0.4	4.9
1312	TRINITY_DN2329_c0_g1_i7	Procardosin-A	0.2	4.9
1313	TRINITY_DN4146_c3_g1_i1	No hit	0.4	4.9
1314	TRINITY_DN532_c0_g3_i1	No hit	0.2	4.9
1315	TRINITY_DN46267_c0_g1_i1	No hit	0.0	4.9
1316	TRINITY_DN55108_c0_g1_i1	No hit	0.0	4.9
1317	TRINITY_DN7086_c0_g1_i47	Ras-related protein Rab-21	0.0	4.8
1318	TRINITY_DN26190_c0_g1_i1	Facilitated trehalose transporter Tret1-like	0.2	4.8
1319	TRINITY_DN5024_c1_g2_i1	No hit	0.2	4.8
1320	TRINITY_DN28069_c0_g1_i1	No hit	0.2	4.8
1321	TRINITY_DN38863_c0_g1_i1	No hit	0.3	4.8
1322	TRINITY_DN2194_c0_g1_i2	No hit	0.4	4.8
1323	TRINITY_DN50574_c0_g1_i1	No hit	0.5	4.8
1324	TRINITY_DN3778_c0_g2_i1	No hit	0.3	4.8
1325	TRINITY_DN31933_c0_g2_i1	No hit	0.0	4.8
1326	TRINITY_DN42133_c0_g1_i1	No hit	0.4	4.7
1327	TRINITY_DN41017_c0_g1_i1	No hit	0.3	4.7
1328	TRINITY_DN6925_c0_g1_i1	Protein MRVI1	0.5	4.7
1329	TRINITY_DN17215_c0_g1_i1	No hit	0.3	4.7
1330	TRINITY_DN6818_c0_g1_i2	No hit	0.0	4.7
1331	TRINITY_DN53023_c0_g1_i1	No hit	0.0	4.7
1332	TRINITY_DN612_c0_g1_i2	Rap1 GTPase-activating protein 2 isoform X3	0.5	4.6
1333	TRINITY_DN2868_c0_g1_i1	Elongation of very long chain fatty acids protein 6	0.3	4.6
1334	TRINITY_DN48203_c0_g1_i1	No hit	0.4	4.6
1335	TRINITY_DN12255_c0_g1_i1	No hit	0.4	4.6
1336	TRINITY_DN1227_c0_g1_i3	Serine/threonine-protein kinase tricorner isoform X1	0.5	4.6
1337	TRINITY_DN11890_c0_g2_i1	No hit	0.1	4.6
1338	TRINITY_DN338_c1_g1_i1	No hit	0.6	4.6
1339	TRINITY_DN29800_c0_g1_i1	No hit	0.2	4.6
1340	TRINITY_DN33850_c0_g1_i1	Sex peptide receptor	0.2	4.5
1341	TRINITY_DN297_c7_g1_i1	No hit	0.2	4.5
1342	TRINITY_DN9889_c0_g1_i3	No hit	0.4	4.5
1343	TRINITY_DN11720_c0_g1_i2	Adenosylhomocysteinase	0.3	4.5
1344	TRINITY_DN6425_c0_g2_i10	Plasma membrane calcium-transporting ATPase 2 isoform X2	0.0	4.5
1345	TRINITY_DN5383_c1_g1_i1	No hit	0.3	4.5
1346	TRINITY_DN22163_c0_g1_i1	Uncharacterized protein LOC106681469	0.1	4.5
1347	TRINITY_DN29350_c0_g3_i1	Uncharacterized protein LOC106681750	0.4	4.5
1348	TRINITY_DN4944_c0_g1_i37	Dual 3',5'-cyclic-AMP and -GMP phosphodiesterase 11 isoform X4	0.2	4.4
1349	TRINITY_DN3178_c1_g1_i5	1-acyl-sn-glycerol-3-phosphate acyltransferase alpha	0.2	4.4
1350	TRINITY_DN20219_c0_g1_i1	No hit	0.5	4.4
1351	TRINITY_DN1431_c0_g2_i11	Tropomodulin-1 isoform X7	0.0	4.4
1352	TRINITY_DN38537_c0_g1_i1	No hit	0.0	4.4
1353	TRINITY_DN14368_c0_g1_i1	No hit	0.1	4.4
1354	TRINITY_DN4658_c0_g1_i1	No hit	0.5	4.4
1355	TRINITY_DN879_c0_g1_i7	Pumilio homolog 2 isoform X5	0.0	4.4
1356	TRINITY_DN54425_c0_g1_i1	No hit	0.0	4.3
1357	TRINITY_DN50975_c0_g1_i1	No hit	0.1	4.3
1358	TRINITY_DN5072_c0_g1_i1	Adenylate cyclase type 8-like isoform X2	0.2	4.3
1359	TRINITY_DN18434_c0_g1_i1	No hit	0.6	4.3
1360	TRINITY_DN9820_c0_g1_i1	No hit	0.6	4.2
1361	TRINITY_DN35304_c0_g1_i1	No hit	0.4	4.2
1362	TRINITY_DN2937_c0_g1_i1	No hit	0.3	4.2
1363	TRINITY_DN2074_c0_g1_i78	Piezo-type mechanosensitive ion channel component isoform X5	0.7	4.2
1364	TRINITY_DN14521_c0_g1_i2	Ascorbate-specific transmembrane electron transporter 1-like	0.1	4.2
1365	TRINITY_DN748_c9_g1_i1	No hit	0.3	4.2
1366	TRINITY_DN43811_c0_g1_i1	No hit	0.3	4.2
1367	TRINITY_DN8574_c0_g1_i44	Trafficking kinesin-binding protein milt-like	0.1	4.2
1368	TRINITY_DN26205_c0_g1_i1	No hit	0.3	4.2
1369	TRINITY_DN22397_c0_g2_i1	No hit	0.5	4.2
1370	TRINITY_DN4162_c0_g1_i15	Hydroxysteroid dehydrogenase-like protein 2 isoform X2	0.0	4.2
1371	TRINITY_DN19450_c0_g2_i1	NPC intracellular cholesterol transporter 1 homolog 1b-like	0.7	4.1
1372	TRINITY_DN3530_c0_g1_i2	Solute carrier family 35 member C2	0.1	4.1
1373	TRINITY_DN42807_c0_g1_i1	Acylphosphatase-2	0.3	4.1
1374	TRINITY_DN19651_c0_g1_i1	No hit	0.0	4.1
1375	TRINITY_DN10586_c0_g2_i1	No hit	0.0	4.1
1376	TRINITY_DN18157_c0_g1_i1	No hit	0.7	4.1
1377	TRINITY_DN12007_c0_g4_i1	No hit	0.1	4.1
1378	TRINITY_DN14372_c1_g1_i1	No hit	0.2	4.1
1379	TRINITY_DN1710_c0_g1_i17	No hit	0.3	4.1
1380	TRINITY_DN48824_c0_g1_i1	No hit	0.1	4.1
1381	TRINITY_DN7483_c4_g1_i1	No hit	0.1	4.1
1382	TRINITY_DN12812_c0_g1_i1	No hit	0.0	4.1

1383	TRINITY_DN11568_c0_g1_i1	Myosin-I heavy chain isoform X1	0.8	4.0
1384	TRINITY_DN16489_c0_g1_i1	No hit	0.2	4.0
1385	TRINITY_DN4808_c0_g1_i1	Onchocystatin	0.4	4.0
1386	TRINITY_DN30348_c0_g1_i1	No hit	0.2	4.0
1387	TRINITY_DN29350_c0_g2_i1	Uncharacterized protein LOC106681750	0.5	4.0
1388	TRINITY_DN7467_c0_g1_i2	Uncharacterized protein LOC106687750 isoform X3	0.0	4.0
1389	TRINITY_DN22814_c0_g1_i1	No hit	0.0	4.0
1390	TRINITY_DN18266_c0_g1_i1	No hit	0.4	3.9
1391	TRINITY_DN20953_c0_g1_i1	No hit	0.2	3.9
1392	TRINITY_DN2594_c0_g1_i2	Follicle-stimulating hormone receptor-like	0.2	3.9
1393	TRINITY_DN14007_c0_g3_i1	Indole-3-acetaldehyde oxidase-like isoform X2	0.5	3.9
1394	TRINITY_DN2106_c0_g1_i11	No hit	0.2	3.9
1395	TRINITY_DN4162_c0_g1_i16	Hydroxysteroid dehydrogenase-like protein 2 isoform X2	0.0	3.9
1396	TRINITY_DN30660_c0_g1_i1	Uncharacterized protein LOC106678018	0.2	3.9
1397	TRINITY_DN6311_c0_g1_i29	Protein FAM13A, partial	0.7	3.9
1398	TRINITY_DN31240_c0_g1_i1	No hit	0.0	3.9
1399	TRINITY_DN1580_c0_g1_i6	Nucleolar and coiled-body phosphoprotein 1 isoform X1	0.2	3.8
1400	TRINITY_DN10_c0_g1_i6	Multidrug resistance-associated protein 1	0.6	3.8
1401	TRINITY_DN679_c0_g1_i4	Probable actin-related protein 2/3 complex subunit 2	0.6	3.8
1402	TRINITY_DN8349_c0_g1_i4	Cyclin-dependent kinase 17-like isoform X2	0.1	3.8
1403	TRINITY_DN842_c0_g1_i2	Putative inorganic phosphate cotransporter isoform X1	0.1	3.8
1404	TRINITY_DN41059_c0_g1_i1	No hit	0.3	3.8
1405	TRINITY_DN5551_c1_g1_i3	Inositol-trisphosphate 3-kinase B	0.2	3.8
1406	TRINITY_DN4258_c1_g1_i2	Brain-specific angiogenesis inhibitor 1-associated protein 2 isoform X2	0.3	3.8
1407	TRINITY_DN46654_c0_g1_i1	No hit	0.0	3.8
1408	TRINITY_DN17115_c2_g1_i1	No hit	0.2	3.7
1409	TRINITY_DN20845_c0_g1_i1	4-nitrophenylphosphatase-like	0.1	3.7
1410	TRINITY_DN15360_c0_g1_i1	No hit	0.0	3.7
1411	TRINITY_DN46686_c0_g1_i1	No hit	0.2	3.7
1412	TRINITY_DN3131_c0_g1_i3	Ets DNA-binding protein pokkuri	0.3	3.7
1413	TRINITY_DN3254_c0_g1_i1	Uncharacterized protein LOC112210029	0.2	3.7
1414	TRINITY_DN35388_c0_g1_i1	Uncharacterized protein LOC106677841	0.4	3.7
1415	TRINITY_DN4576_c1_g1_i3	Peroxisomal acyl-coenzyme A oxidase 3-like	0.8	3.7
1416	TRINITY_DN53715_c0_g1_i1	Uncharacterized protein LOC106685531 isoform X2	0.2	3.6
1417	TRINITY_DN35798_c0_g1_i1	No hit	0.4	3.6
1418	TRINITY_DN31721_c0_g1_i1	No hit	0.1	3.6
1419	TRINITY_DN8492_c0_g1_i1	No hit	0.4	3.6
1420	TRINITY_DN2470_c0_g1_i22	SMC5-SMC6 complex localization factor protein 1-like isoform X2	0.1	3.6
1421	TRINITY_DN4023_c0_g1_i6	No hit	0.0	3.6
1422	TRINITY_DN2185_c0_g1_i12	Triple functional domain protein isoform X5	0.3	3.6
1423	TRINITY_DN17130_c0_g1_i2	FH1/FH2 domain-containing protein 3 isoform X6	0.2	3.6
1424	TRINITY_DN2992_c0_g1_i3	No hit	0.2	3.5
1425	TRINITY_DN7273_c0_g1_i1	No hit	0.3	3.5
1426	TRINITY_DN10846_c0_g1_i1	No hit	0.3	3.5
1427	TRINITY_DN13246_c0_g2_i1	GTP cyclohydrolase 1 isoform X3	0.4	3.5
1428	TRINITY_DN18047_c0_g1_i1	No hit	0.3	3.5
1429	TRINITY_DN2753_c0_g1_i1	RCC1 and BTB domain-containing protein 1-like isoform X1	0.1	3.4
1430	TRINITY_DN25545_c0_g1_i2	No hit	0.1	3.4
1431	TRINITY_DN6517_c0_g1_i1	Uncharacterized protein LOC106686360	0.5	3.4
1432	TRINITY_DN5154_c0_g1_i2	Discoidin domain-containing receptor 2-like	0.2	3.4
1433	TRINITY_DN374_c0_g3_i1	No hit	0.2	3.4
1434	TRINITY_DN12158_c0_g4_i1	No hit	0.1	3.3
1435	TRINITY_DN38464_c0_g1_i1	No hit	0.3	3.3
1436	TRINITY_DN2780_c0_g1_i1	No hit	0.1	3.3
1437	TRINITY_DN1482_c1_g1_i1	No hit	0.2	3.3
1438	TRINITY_DN1483_c0_g1_i5	Protein FAM214A isoform X1	0.0	3.3
1439	TRINITY_DN540_c0_g2_i1	No hit	0.3	3.3
1440	TRINITY_DN9853_c0_g1_i1	No hit	0.4	3.3
1441	TRINITY_DN4699_c0_g1_i1	No hit	0.2	3.3
1442	TRINITY_DN15533_c0_g1_i1	No hit	0.3	3.3
1443	TRINITY_DN6487_c0_g2_i1	No hit	0.3	3.3
1444	TRINITY_DN44001_c0_g1_i1	No hit	0.0	3.3
1445	TRINITY_DN4612_c0_g1_i6	PDZ and LIM domain protein 3 isoform X5	0.2	3.2
1446	TRINITY_DN3677_c0_g1_i4	Multiple C2 and transmembrane domain-containing protein isoform X5	0.1	3.2
1447	TRINITY_DN5150_c0_g1_i6	Centrosomin isoform X4	0.4	3.2
1448	TRINITY_DN50765_c0_g1_i1	No hit	0.2	3.2
1449	TRINITY_DN6968_c0_g1_i1	No hit	0.4	3.2
1450	TRINITY_DN4523_c0_g1_i2	Uncharacterized protein LOC106690537 isoform X2	0.1	3.2
1451	TRINITY_DN1799_c0_g1_i1	No hit	0.4	3.2
1452	TRINITY_DN12158_c0_g1_i39	Acetyl-CoA carboxylase 1	0.2	3.2
1453	TRINITY_DN7410_c0_g1_i8	Protein lethal(2) giant larvae isoform X4	0.3	3.1
1454	TRINITY_DN30186_c0_g1_i1	No hit	0.0	3.1
1455	TRINITY_DN1676_c0_g1_i1	Protein virilizer isoform X3	0.5	3.1
1456	TRINITY_DN1912_c0_g1_i1	Beta-glucuronidase-like isoform X1	0.6	3.1
1457	TRINITY_DN4844_c0_g1_i3	Transmembrane protein 135-like	0.3	3.1
1458	TRINITY_DN2264_c0_g1_i2	Serine/threonine-protein phosphatase 6 regulatory ankyrin repeat subunit B-like	0.4	3.1
1459	TRINITY_DN5154_c0_g1_i1	Discoidin domain-containing receptor 2-like	0.1	3.1

1460	TRINITY_DN43817_c0_g1_i1	Facilitated trehalose transporter Tret1-like	0.1	3.1
1461	TRINITY_DN2915_c0_g1_i4	Chitinase-like protein EN03	0.3	3.1
1462	TRINITY_DN52572_c0_g1_i1	No hit	0.0	3.1
1463	TRINITY_DN3332_c0_g1_i2	Carboxypeptidase E	0.1	3.1
1464	TRINITY_DN3908_c0_g1_i1	No hit	0.3	3.0
1465	TRINITY_DN10075_c0_g1_i3	Fibrinolytic enzyme, isozyme C	0.3	3.0
1466	TRINITY_DN27_c0_g1_i10	Anoctamin-5-like isoform X8	0.1	3.0
1467	TRINITY_DN28277_c0_g1_i1	No hit	0.1	3.0
1468	TRINITY_DN22330_c0_g1_i1	No hit	0.3	3.0
1469	TRINITY_DN28888_c0_g1_i1	No hit	0.0	3.0
1470	TRINITY_DN12133_c0_g1_i1	Caspase-1	0.4	3.0
1471	TRINITY_DN18621_c0_g2_i1	No hit	0.1	3.0
1472	TRINITY_DN14858_c0_g1_i1	No hit	0.2	2.9
1473	TRINITY_DN2206_c0_g1_i47	Dystonin isoform X10	0.4	2.9
1474	TRINITY_DN4531_c2_g1_i1	No hit	0.2	2.9
1475	TRINITY_DN44738_c0_g1_i1	No hit	0.2	2.9
1476	TRINITY_DN3084_c0_g2_i1	No hit	0.5	2.9
1477	TRINITY_DN243_c0_g1_i54	Uncharacterized protein LOC106687821 isoform X12	0.0	2.9
1478	TRINITY_DN3511_c0_g1_i1	No hit	0.1	2.8
1479	TRINITY_DN3481_c0_g1_i5	Uncharacterized protein LOC106683278 isoform X2	0.1	2.8
1480	TRINITY_DN2131_c0_g1_i2	No hit	0.1	2.8
1481	TRINITY_DN2702_c1_g1_i2	Protein 4.1 homolog isoform X1	0.6	2.8
1482	TRINITY_DN89_c0_g1_i46	Methylcrotonoyl-CoA carboxylase beta chain, mitochondrial	0.0	2.7
1483	TRINITY_DN833_c0_g1_i3	Tubulin alpha-1 chain-like	0.1	2.7
1484	TRINITY_DN16275_c0_g2_i21	Protein-methionine sulfoxide oxidase mical2b isoform X1	0.3	2.7
1485	TRINITY_DN31868_c0_g1_i1	No hit	0.2	2.7
1486	TRINITY_DN1054_c0_g1_i10	Titin isoform X7	0.0	2.7
1487	TRINITY_DN3658_c0_g1_i31	Trypsin alpha-3-like isoform X2	0.2	2.6
1488	TRINITY_DN9194_c0_g1_i10	Myogenesis-regulating glycosidase isoform X2	0.1	2.5
1489	TRINITY_DN11578_c0_g1_i1	No hit	0.0	2.4

[1] In the order of average expression levels in the main tract.

[2] Average expression levels of four individuals.

[3] Significantly higher expression in the main tract than in the crypts (FDR $q < 0.01$).

Table S7. Differentially expressed insect genes upregulated in the crypts and the main tract of the adult symbiotic organ of *P. stali* .

Rank [1]	Contig ID	Description	TPM [2][3]	
			Crypts	Main tract
1	TRINITY_DN2568_c0_g1_i1	No hit	1121.5	39508.5
2	TRINITY_DN441_c1_g2_i11	No hit	1090.3	42070.3
3	TRINITY_DN179_c1_g1_i1	Uncharacterized protein	229.8	5827.7
4	TRINITY_DN2125_c0_g1_i1	Cathepsin L1-like	156.3	5400.7
5	TRINITY_DN14573_c0_g1_i1	Cathepsin B-like	75.6	3910.1
6	TRINITY_DN44596_c0_g1_i1	Probable salivary secreted peptide	21.1	1111.0
7	TRINITY_DN223_c0_g3_i2	Endochitinase	68.5	17.5
8	TRINITY_DN1345_c0_g1_i6	Uncharacterized protein	75.8	2685.6
9	TRINITY_DN7901_c0_g1_i1	No hit	22.1	560.3
10	TRINITY_DN5589_c0_g1_i4	Glutamine synthetase isoform X1	289.6	159.4
11	TRINITY_DN32999_c0_g1_i1	Uncharacterized protein	60.2	2731.2
12	TRINITY_DN5750_c0_g1_i1	No hit	103.5	4910.2
13	TRINITY_DN8710_c0_g2_i2	Laccase-4	17.4	859.5
14	TRINITY_DN14573_c0_g2_i1	Uncharacterized protein	30.7	1064.4
15	TRINITY_DN7917_c0_g1_i2	No hit	57.3	1400.8
16	TRINITY_DN36827_c0_g1_i1	No hit	13.1	1087.9
17	TRINITY_DN50111_c0_g1_i1	Probable salivary secreted peptide	11.0	735.9
18	TRINITY_DN38775_c0_g1_i1	Putative serine protease	10.3	366.4
19	TRINITY_DN4566_c0_g1_i1	Uncharacterized protein	185.9	6365.2
20	TRINITY_DN3330_c0_g1_i2	Trypsin-1-like	20.6	563.2
21	TRINITY_DN15747_c0_g1_i1	Aspartic proteinase A3-like	19.6	861.7
22	TRINITY_DN2734_c0_g1_i1	No hit	54.7	2743.3
23	TRINITY_DN42448_c0_g1_i1	Venom carboxylesterase-6-like	6.6	236.1
24	TRINITY_DN46418_c0_g1_i1	Probable salivary secreted peptide	14.7	595.0
25	TRINITY_DN3347_c0_g1_i2	Uncharacterized protein	70.3	2122.3
26	TRINITY_DN1554_c1_g1_i1	Cathepsin L1	53.7	943.6
27	TRINITY_DN55185_c0_g1_i1	Uncharacterized protein	42.9	930.5
28	TRINITY_DN42841_c0_g1_i1	Cathepsin L1-like	8.7	363.8
29	TRINITY_DN25545_c0_g1_i1	Uncharacterized protein	13.6	615.4
30	TRINITY_DN29861_c0_g1_i1	Uncharacterized protein	19.6	184.6
31	TRINITY_DN15137_c0_g1_i1	Uncharacterized protein	8.4	231.7
32	TRINITY_DN10486_c0_g1_i1	Uncharacterized protein	31.8	641.1
33	TRINITY_DN2329_c0_g1_i6	Procardosin-A	7.5	254.6
34	TRINITY_DN223_c0_g1_i6	Endochitinase	41.3	8.8
35	TRINITY_DN2122_c0_g1_i1	2-acylglycerol O-acyltransferase 1-like	5.9	146.8
36	TRINITY_DN287_c0_g1_i1	Alpha-glucosidase-like	3.1	104.3
37	TRINITY_DN2908_c3_g4_i1	Probable cytochrome P450	4.0	115.7
38	TRINITY_DN3347_c0_g1_i1	Uncharacterized protein	2.2	52.0
39	TRINITY_DN3695_c0_g1_i1	No hit	9.7	365.0
40	TRINITY_DN4459_c0_g1_i1	Uncharacterized protein	3.2	142.2
41	TRINITY_DN7823_c0_g1_i1	No hit	3.0	174.1
42	TRINITY_DN49824_c0_g1_i1	Legumain-like	6.2	246.7
43	TRINITY_DN223_c0_g1_i5	Endochitinase	4.1	0.4
44	TRINITY_DN5257_c0_g2_i1	Uncharacterized protein	11.4	448.5
45	TRINITY_DN13228_c0_g1_i1	No hit	1.9	72.9
46	TRINITY_DN362_c0_g1_i1	Uncharacterized protein	24.0	9.7
47	TRINITY_DN38752_c0_g1_i1	Uncharacterized protein	29.7	9.3
48	TRINITY_DN5203_c0_g1_i1	No hit	3.0	146.9
49	TRINITY_DN12648_c1_g2_i1	No hit	8.2	1.5
50	TRINITY_DN5968_c0_g1_i1	Uncharacterized protein	11.9	527.4
51	TRINITY_DN5727_c0_g1_i3	No hit	10.1	2.9
52	TRINITY_DN5736_c0_g1_i2	Alpha-tocopherol transfer protein-like	2.2	57.2
53	TRINITY_DN10039_c0_g1_i1	Uncharacterized protein	6.3	394.7

54	TRINITY_DN26236_c0_g1_i2	Uncharacterized protein	1.8	80.4
55	TRINITY_DN24582_c0_g1_i1	No hit	9.6	423.5
56	TRINITY_DN14751_c0_g1_i1	No hit	8.2	6.4
57	TRINITY_DN35973_c0_g1_i1	No hit	27.4	1681.2
58	TRINITY_DN4696_c0_g1_i1	Small nuclear ribonucleoprotein	11.5	13.3
59	TRINITY_DN47660_c0_g1_i1	Uncharacterized protein	1.9	55.2
60	TRINITY_DN9852_c0_g3_i3	Cardioactive peptide	8.5	263.2
61	TRINITY_DN5057_c0_g1_i1	No hit	10.0	19.2
62	TRINITY_DN50805_c0_g1_i1	Protein takeout-like	10.9	9.5
63	TRINITY_DN13149_c0_g1_i1	Phospholipase A1-like isoform X1	8.8	11.1
64	TRINITY_DN55140_c0_g1_i1	No hit	11.7	485.1
65	TRINITY_DN1496_c0_g1_i5	No hit	7.2	3.4
66	TRINITY_DN927_c0_g1_i1	Protein MEMO1	4.8	3.6
67	TRINITY_DN3330_c0_g1_i3	Trypsin-1-like	1.5	42.5
68	TRINITY_DN1599_c0_g1_i6	Acylamino-acid-releasing enzyme-like isoform X1	2.7	0.0
69	TRINITY_DN8946_c0_g1_i1	No hit	0.9	53.6
70	TRINITY_DN12680_c0_g1_i1	Trafficking protein particle complex subunit 2	0.2	1.9
71	TRINITY_DN1654_c0_g1_i1	Endochitinase	4.0	0.6
72	TRINITY_DN2106_c0_g1_i8	Aspartic proteinase A3 isoform X2	2.3	106.4
73	TRINITY_DN5460_c0_g1_i3	ADP-ribose pyrophosphatase, mitochondrial isoform X1	2.8	3.0
74	TRINITY_DN12648_c1_g1_i9	No hit	4.3	0.0
75	TRINITY_DN15934_c0_g1_i2	Alanine aminotransferase 1-like	6.1	4.7
76	TRINITY_DN11243_c1_g2_i1	UDP-glucuronosyltransferase	1.4	29.2
77	TRINITY_DN3332_c0_g1_i2	Carboxypeptidase E	0.1	3.1
78	TRINITY_DN42414_c0_g1_i1	Maltase A1-like	2.4	112.0
79	TRINITY_DN54035_c0_g1_i1	Cathepsin L1-like	1.4	34.6
80	TRINITY_DN8634_c0_g2_i2	Sodium/potassium/calcium exchanger 4-like isoform X2	3.2	1.2
81	TRINITY_DN2088_c0_g1_i12	Copper-transporting ATPase 1 isoform X2	0.3	18.6
82	TRINITY_DN15322_c0_g1_i1	Cathepsin L1 isoform X1	1.2	55.5
83	TRINITY_DN2160_c0_g1_i9	CAAX prenyl protease 1 homolog	1.1	2.3
84	TRINITY_DN32400_c0_g1_i1	Cathepsin L1-like	0.7	28.7
85	TRINITY_DN19240_c0_g1_i3	Uncharacterized protein	0.4	8.6
86	TRINITY_DN468_c1_g1_i14	Alanine aminotransferase 1-like	3.8	4.4
87	TRINITY_DN7379_c0_g1_i16	Serine hydroxymethyltransferase isoform X1	6.1	1.2
88	TRINITY_DN362_c0_g1_i2	Uncharacterized protein	5.6	2.4
89	TRINITY_DN4215_c0_g1_i1	Uncharacterized protein	0.4	15.3
90	TRINITY_DN46250_c0_g1_i1	Uncharacterized protein	1.9	63.3
91	TRINITY_DN39051_c0_g1_i1	Venom serine carboxypeptidase	0.8	26.8
92	TRINITY_DN2208_c0_g1_i7	Uncharacterized protein	1.0	16.9
93	TRINITY_DN5269_c0_g1_i12	Uncharacterized protein	3.3	3.6
94	TRINITY_DN3854_c0_g1_i6	No hit	8.1	3.7
95	TRINITY_DN1496_c0_g1_i25	Retinal dehydrogenase 1-like	2.4	0.7
96	TRINITY_DN25496_c0_g1_i1	Spondin-1	2.9	83.7
97	TRINITY_DN27227_c0_g1_i1	Monocarboxylate transporter 14 isoform X2	0.8	23.4
98	TRINITY_DN36239_c0_g1_i1	No hit	2.5	1.9
99	TRINITY_DN8614_c0_g1_i17	Monocarboxylate transporter 13	0.8	0.1
100	TRINITY_DN49863_c0_g1_i1	Elongation of very long chain fatty acids protein	2.6	1.3

[1] In the order of average expression levels in the whole adult symbiotic organ.

[2] Average expression levels of four individuals.

[3] Red and blue show significantly higher expression in the crypts and that in the main tract, respectively (FDR $q < 0.01$).

Table S8. Differentially expressed symbiont genes significantly upregulated in the nymphal symbiotic organ of *P. stali.*

Rank [1]	ID	Gene	Function	TPM [2]				
				4th instar	5th instar [3]	Adult	*E93* RNAi	*Kr-h1* RNAi
1	X08790	no hit	Uncharacterized	51.2	44.2	26.4	64.2	72.0
2	X13470	trpE	Anthranilate synthase component 1	23.1	29.7	25.4	19.9	11.0
3	X17450	cheW	Chemotaxis protein	24.5	24.9	18.7	16.6	21.1
4	X00380	virB4	TypeIV secretion system protein	10.0	10.5	8.1	9.1	13.4

[1] In the order of average expression levels in 5th instar nymphs.

[2] Average expression levels of four individuals.

[3] Significantly higher expression in 5th instar nymphs than in adults (FDR $q < 0.01$).

Table S9. Differentially expressed symbiont genes significantly upregulated in the adult symbiotic organ of *P. stali*.

Rank [1]	ID	Gene	Function	TPM [2]				
				4th instar	5th instar	Adult [3]	*E93* RNAi	*Kr-h1* RNAi
1	X30290	uspB	Universal Stress Protein B	75.8	64.3	358.4	156.7	184.7
2	X04040	yeiH	Inner Membrane Protein	6.1	6.4	314.6	26.1	11.5
3	X02740	cysK	Cysteine Synthase A	77.8	55.7	302.4	160.1	159.9
4	X32160	deoB	Phosphopentomutase	83.8	58.0	209.5	124.8	103.3
5	X07860	tauA	Taurine-Binding Periplasmic Protein	24.0	16.9	183.4	89.3	89.0
6	X07890	tauD	Alpha-Ketoglutarate-Dependent Taurine Dioxygenase	29.1	18.8	180.0	74.5	112.5
7	X17380	argD	Succinylornithine Transaminase	35.2	19.4	177.6	37.2	95.1
8	X17330	spy	Periplasmic Chaperone	54.2	29.8	160.9	102.8	85.4
9	X29510	cysD	Sulfate Adenylyltransferase Subunit 2	33.3	26.0	153.3	80.2	77.5
10	X02630	cysP	Thiosulfate-Binding Protein	23.7	21.9	138.8	104.7	64.8
11	X02660	cysA	Sulfate/Thiosulfate Import Atp-Binding Protein	16.8	17.3	111.7	59.0	69.5
12	X32150	deoD	Purine Nucleoside Phosphorylase	43.5	31.6	110.6	62.3	84.6
13	X29500	cysG	Siroheme Synthase	25.2	20.6	101.9	69.4	45.5
14	X11230	no hit	Uncharacterized	32.4	17.2	101.3	59.3	72.4
15	X17370	astA	Arginine N-Succinyltransferase	17.4	10.7	100.3	20.0	54.3
16	X29520	cysN	Sulfate Adenylyltransferase Subunit 1	22.8	18.2	94.3	49.4	54.2
17	X07870	tauB	Taurine Import Atp-Binding Protein	11.1	6.7	85.6	35.4	40.3
18	X07880	tauC	Taurine Transport System Permease Protein	10.1	6.3	75.0	36.2	39.4
19	X17340	astE	Succinylglutamate Desuccinylase	22.9	18.6	74.3	26.1	38.8
20	X17360	astD	N-Succinylglutamate 5-Semialdehyde Dehydrogenase	10.6	6.9	73.9	11.5	37.3
21	X40310	no hit	Uncharacterized	23.6	14.6	69.9	23.5	26.1
22	X40320	amiC	Amidase Expression-Regulating Protein	27.0	16.0	67.0	21.4	28.6
23	X16200	gpr	Glyceraldehyde 3-Phosphate Reductase	5.4	4.1	61.4	10.5	9.4
24	X17350	astB	N-Succinylarginine Dihydrolase	9.1	5.9	59.2	11.6	30.6
25	X02640	cysU	Sulfate Transport System Permease Protein	7.6	7.0	48.7	35.3	30.2
26	X23560	thiG	Thiazole Synthase	10.2	6.3	43.5	9.7	27.8
27	X02650	cysW	Sulfate Transport System Permease Protein	8.2	8.0	43.0	28.6	29.3
28	X23580	thiO	Putative Thiamine Biosynthesis Oxidoreductase	10.2	7.7	42.7	9.7	32.8
29	X40300	accC	Biotin Carboxylase	12.8	8.9	38.2	10.0	14.8
30	X23550	thiF	Thiazole Biosynthesis Adenylyltransferase	8.1	5.4	28.1	7.3	23.1
31	X37830	dicA	Hth-Type Transcriptional Regulator	5.3	4.8	27.6	6.8	8.8
32	X36160	no hit	Uncharacterized	2.2	1.6	26.8	12.5	10.5
33	X23540	no hit	Uncharacterized	8.3	5.3	25.3	11.5	17.1
34	X04390	tetR	Tetracycline Repressor Protein	4.3	2.8	21.7	11.5	13.9
35	X40290	atzF	Allophanate Hydrolase	8.4	5.2	20.4	6.8	7.7
36	X06410	cbl	Hth-Type Transcriptional Regulator	3.5	2.6	14.3	13.5	14.4
37	X36170	metQ	D-Methionine-Binding Lipoprotein	1.5	0.7	14.2	6.0	7.5
38	X37840	no hit	Uncharacterized	1.5	1.5	12.2	3.5	4.9
39	X37850	no hit	Uncharacterized	1.6	1.0	10.4	3.6	5.2
40	X16260	ubiD	3-Octaprenyl-4-Hydroxybenzoate Carboxy-Lyase	12.6	1.8	9.4	3.1	10.3
41	X13250	ubiX	Flavin Prenyltransferase	1.3	1.0	8.3	3.8	3.8
42	X03520	no hit	Uncharacterized	1.4	1.0	7.4	1.8	3.5
43	X05990	ydfG	Nadp-Dependent 3-Hydroxy Acid Dehydrogenase	2.3	1.4	6.9	3.0	4.3
44	X12430	ychM	Putative Sulfate Transporter	1.6	1.7	6.6	2.9	4.6
45	X33820	copA	Copper-Exporting P-Type Atpase	2.4	1.9	6.2	3.4	6.2

[1] In the order of average expression levels in adults.

[2] Average expression levels of four individuals.

[3] Significantly higher expression in adults than in 5th instar nymphs (FDR $q < 0.01$).

Table S10. RNA sequencing data generated in this study.

No.	Tissue	Developmental stage	Treatment	Sex	Individual	Date	No. of pairs	Sequence	Accession No.
1	Symbiotic organ	4th instar day 3	*bla* RNAi(Control)	Unknown	Ps1	2021/5/14	26,172,722	HiSeq 150 bp paired	DRR446133
2	Symbiotic organ	4th instar day 3	*bla* RNAi(Control)	Unknown	Ps2	2021/5/14	16,249,415	HiSeq 150 bp paired	DRR446134
3	Symbiotic organ	4th instar day 3	*bla* RNAi(Control)	Unknown	Ps3	2021/5/14	22,688,812	HiSeq 150 bp paired	DRR446135
4	Symbiotic organ	4th instar day 3	*bla* RNAi(Control)	Unknown	Ps4	2021/5/14	19,780,711	HiSeq 150 bp paired	DRR446136
5	Symbiotic organ	5th instar day 3	*bla* RNAi(Control)	Male	Ps5	2021/5/14	24,861,681	HiSeq 150 bp paired	DRR446137
6	Symbiotic organ	5th instar day 3	*bla* RNAi(Control)	Female	Ps6	2021/5/14	19,065,340	HiSeq 150 bp paired	DRR446138
7	Symbiotic organ	5th instar day 3	*bla* RNAi(Control)	Male	Ps7	2021/5/14	14,892,951	HiSeq 150 bp paired	DRR446139
8	Symbiotic organ	5th instar day 3	*bla* RNAi(Control)	Female	Ps8	2021/5/14	15,234,636	HiSeq 150 bp paired	DRR446140
9	Symbiotic organ	Adult day 3	*bla* RNAi(Control)	Female	Ps9	2021/5/14	26,518,643	HiSeq 150 bp paired	DRR446141
10	Symbiotic organ	Adult day 3	*bla* RNAi(Control)	Male	Ps10	2021/5/14	21,005,422	HiSeq 150 bp paired	DRR446142
11	Symbiotic organ	Adult day 3	*bla* RNAi(Control)	Female	Ps11	2021/5/14	20,389,626	HiSeq 150 bp paired	DRR446143
12	Symbiotic organ	Adult day 3	*bla* RNAi(Control)	Male	Ps12	2021/5/14	19,635,382	HiSeq 150 bp paired	DRR446144
13	Symbiotic organ	5th instar day 3	*Kr-h1* RNAi	Male	Ps13	2021/5/14	18,924,668	HiSeq 150 bp paired	DRR446145
14	Symbiotic organ	5th instar day 3	*Kr-h1* RNAi	Male	Ps14	2021/5/14	16,231,489	HiSeq 150 bp paired	DRR446146
15	Symbiotic organ	5th instar day 3	*Kr-h1* RNAi	Male	Ps15	2021/5/14	14,849,507	HiSeq 150 bp paired	DRR446147
16	Symbiotic organ	5th instar day 3	*Kr-h1* RNAi	Female	Ps16	2021/5/14	18,947,945	HiSeq 150 bp paired	DRR446148
17	Symbiotic organ	6th instar day 3	*E93* RNAi	Female	Ps17	2021/5/14	18,917,956	HiSeq 150 bp paired	DRR446149
18	Symbiotic organ	6th instar day 3	*E93* RNAi	Male	Ps18	2021/5/14	26,993,874	HiSeq 150 bp paired	DRR446150
19	Symbiotic organ	6th instar day 3	*E93* RNAi	Female	Ps19	2021/5/14	24,401,459	HiSeq 150 bp paired	DRR446151
20	Symbiotic organ	6th instar day 3	*E93* RNAi	Male	Ps20	2021/5/14	27,569,723	HiSeq 150 bp paired	DRR446152
21	Symbiotic organ	4th instar day 2	*bla* RNAi(Control)	Unknown	Ps21	2022/9/2	14,991,455	HiSeq 150 bp paired	DRR446153
22	Symbiotic organ	4th instar day 2	*bla* RNAi(Control)	Unknown	Ps22	2022/9/2	20,036,992	HiSeq 150 bp paired	DRR446154
23	Symbiotic organ	4th instar day 2	*bla* RNAi(Control)	Unknown	Ps23	2022/9/2	30,241,950	HiSeq 150 bp paired	DRR446155
24	Symbiotic organ	4th instar day 3	*bla* RNAi(Control)	Unknown	Ps24	2022/9/2	17,820,282	HiSeq 150 bp paired	DRR446156
25	Symbiotic organ	4th instar day 3	*bla* RNAi(Control)	Unknown	Ps25	2022/9/2	23,842,018	HiSeq 150 bp paired	DRR446157
26	Symbiotic organ	4th instar day 3	*bla* RNAi(Control)	Unknown	Ps26	2022/9/2	19,547,466	HiSeq 150 bp paired	DRR446158
27	Symbiotic organ	5th instar day 2	*bla* RNAi(Control)	Female	Ps27	2022/9/2	20,568,441	HiSeq 150 bp paired	DRR446159
28	Symbiotic organ	5th instar day 2	*bla* RNAi(Control)	Female	Ps28	2022/9/2	22,594,751	HiSeq 150 bp paired	DRR446160
29	Symbiotic organ	5th instar day 2	*bla* RNAi(Control)	Female	Ps29	2022/9/2	18,309,161	HiSeq 150 bp paired	DRR446161
30	Symbiotic organ	5th instar day 3	*bla* RNAi(Control)	Female	Ps30	2022/9/2	23,019,416	HiSeq 150 bp paired	DRR446162
31	Symbiotic organ	5th instar day 3	*bla* RNAi(Control)	Male	Ps31	2022/9/2	19,134,601	HiSeq 150 bp paired	DRR446163
32	Symbiotic organ	5th instar day 3	*bla* RNAi(Control)	Female	Ps32	2022/9/2	19,801,936	HiSeq 150 bp paired	DRR446164
33	Symbiotic organ	4th instar day 2	*Kr-h1* RNAi	Unknown	Ps33	2022/9/2	18,180,709	HiSeq 150 bp paired	DRR446165
34	Symbiotic organ	4th instar day 2	*Kr-h1* RNAi	Unknown	Ps34	2022/9/2	27,712,760	HiSeq 150 bp paired	DRR446166
35	Symbiotic organ	4th instar day 2	*Kr-h1* RNAi	Unknown	Ps35	2022/9/2	18,302,380	HiSeq 150 bp paired	DRR446167
36	Symbiotic organ	4th instar day 3	*Kr-h1* RNAi	Unknown	Ps36	2022/9/2	16,759,546	HiSeq 150 bp paired	DRR446168
37	Symbiotic organ	4th instar day 3	*Kr-h1* RNAi	Unknown	Ps37	2022/9/2	23,754,561	HiSeq 150 bp paired	DRR446169
38	Symbiotic organ	4th instar day 3	*Kr-h1* RNAi	Unknown	Ps38	2022/9/2	21,976,343	HiSeq 150 bp paired	DRR446170
39	Symbiotic organ	5th instar day 2	*E93* RNAi	Female	Ps39	2022/9/2	23,780,160	HiSeq 150 bp paired	DRR446171
40	Symbiotic organ	5th instar day 2	*E93* RNAi	Male	Ps40	2022/9/2	11,578,172	HiSeq 150 bp paired	DRR446172
41	Symbiotic organ	5th instar day 2	*E93* RNAi	Male	Ps41	2022/9/2	27,377,865	HiSeq 150 bp paired	DRR446173
42	Symbiotic organ	5th instar day 3	*E93* RNAi	Male	Ps42	2022/9/2	27,130,414	HiSeq 150 bp paired	DRR446174
43	Symbiotic organ	5th instar day 3	*E93* RNAi	Female	Ps43	2022/9/2	21,766,729	HiSeq 150 bp paired	DRR446175
44	Symbiotic organ	5th instar day 3	*E93* RNAi	Female	Ps44	2022/9/2	23,467,530	HiSeq 150 bp paired	DRR446176
45	Crypts	Adult 1 week	No treatment	Female	Ps45	2022/7/26	36,761,566	HiSeq 150 bp paired	DRR446177
46	Crypts	Adult 1 week	No treatment	Male	Ps46	2022/7/26	33,943,877	HiSeq 150 bp paired	DRR446178
47	Crypts	Adult 1 week	No treatment	Female	Ps47	2022/7/26	53,594,133	HiSeq 150 bp paired	DRR446179
48	Crypts	Adult 1 week	No treatment	Male	Ps48	2022/7/26	39,132,519	HiSeq 150 bp paired	DRR446180
49	Main tract	Adult 1 week	No treatment	Female	Ps45	2022/7/26	37,069,217	HiSeq 150 bp paired	DRR446181
50	Main tract	Adult 1 week	No treatment	Male	Ps46	2022/7/26	32,379,401	HiSeq 150 bp paired	DRR446182
51	Main tract	Adult 1 week	No treatment	Female	Ps47	2022/7/26	29,112,773	HiSeq 150 bp paired	DRR446183
52	Main tract	Adult 1 week	No treatment	Male	Ps48	2022/7/26	28,688,923	HiSeq 150 bp paired	DRR446184
